# HIV-1 Prevalence and Oral Pre-Exposure Prophylaxis Effectiveness and Prevalence of Use Among Key Populations in High-Income Economies (2017–2023): A Systematic Review and Meta-Analysis of Real-World Studies

**DOI:** 10.1093/ofid/ofaf785

**Published:** 2026-01-22

**Authors:** Xiwen Huang, Dylan Mezzio, Juan Yang, Jesse Najarro Cermeño, Soodi Navadeh, Li Tao

**Affiliations:** Gilead Sciences, Inc., Foster City, California, USA; Gilead Sciences, Inc., Foster City, California, USA; Gilead Sciences, Inc., Foster City, California, USA; Gilead Sciences, Parsippany, New Jersey, USA; Gilead Sciences, Inc., Foster City, California, USA; Gilead Sciences, Inc., Foster City, California, USA

**Keywords:** effectiveness, HIV-1 epidemiology, key populations, pre-exposure prophylaxis, real world

## Abstract

In this systematic literature review and meta-analysis, real-world data from high-income economies (excluding the US and Africa) on HIV-1 epidemiology (2019–2023), oral pre-exposure prophylaxis (PrEP) effectiveness (2017–2023), and prevalence of oral PrEP use (2017–2023) were assessed in key populations disproportionately affected by HIV-1. Overall, 204 unique data sources were identified from 38 high-income economies. In key populations, the pooled global HIV-1 prevalence estimate was 5.1% (95% confidence interval: 4.2%–6.1%), ranging from 0.2% in South Korea to 28.9% in Romania. Pooled global prevalence was lowest in transgender men (1.4%) and people in prison (2.2%); 7.0%–7.8% in men who have sex with men, people who inject drugs, sex workers, and transgender women; and highest in individuals who were in multiple key populations (19.4%). Global prevalence of oral PrEP use was 18.2% among key populations, with HIV-1 prevalence <0.4% in PrEP users, indicating high PrEP effectiveness. Targeted prevention strategies are needed to provide global equitable PrEP access and reduce HIV-1 acquisition.

## Teaser

HIV-1 prevalence ranged from <1% to 29% across high-income economies and varied among key populations within regions. Pre-exposure prophylaxis was highly effective in real-world settings, but its use varied widely across populations and regions, highlighting the need for equitable access.

While the number of newly-acquired HIV cases is declining globally, in 2023 there were 1.3 million new diagnoses worldwide, significantly exceeding the 2030 target of fewer than 350 000 new HIV-1 cases set by the United Nations Political Declaration on HIV/AIDS (UNAIDS) [1, 2]. The UNAIDS declaration 2030 goals include 90% of people in need of prevention using a prevention method, including but not limited to pre-exposure prophylaxis (PrEP) [3]. Currently, the most widely available HIV-1 PrEP options are the daily oral medications emtricitabine/tenofovir disoproxil fumarate (F/TDF) and emtricitabine/tenofovir alafenamide (F/TAF), first approved in the US in 2012 and 2019, respectively [4]. These medications have demonstrated high effectiveness in both clinical trials and in real-world investigations when taken consistently as directed [5–8]. In 2021, UNAIDS set a target of 10 million oral PrEP users globally by 2025, yet in 2023, only 3.5 million people have used oral PrEP, most of whom were living in Africa (2.6 million) or in the US (0.5 million) [9, 10].

HIV-1 accounts for 95% of HIV infections globally, with HIV-2 infections primarily limited to Western Africa [11]. In 2023, HIV-1 prevalence was higher in key populations known to be at increased risk of acquiring HIV-1, including men who have sex with men (MSM; 7.7%), people who inject drugs (PWID; 5.0%), people in prison (1.3%), sex workers (3.0%), and transgender people (9.2%), than in the overall adult population (0.8%) [1]. Many high-income countries allocate additional resources to key populations for HIV-1 surveillance and prevention through educational, financial, and destigmatization interventions, though funding allocations vary significantly across regions and populations [9, 12–15]. Despite these efforts, PrEP uptake among key populations in 2024 remained below the 2025 UNAIDS targets [9]. Barriers to PrEP uptake exist at the individual level (eg, low-perceived risk and concerns about side effects), social level (ie, migrant status and stigma related to sexual behavior), and structural level (ie, lack of access to PrEP and to healthcare providers) [16, 17].

Evidence-based evaluation of HIV-1 prevalence, PrEP effectiveness, and PrEP use is needed to identify unmet needs and inform the strategic development of local and regional HIV-1 prevention policies, particularly with the introduction of new modalities such as long-acting PrEP options. While many published studies have assessed real-world evidence for HIV-1 epidemiology and PrEP use in the US and in African countries, fewer studies have been published for other high-income economies. To identify these knowledge gaps, we conducted a systematic literature review and meta-analysis focusing on: (1) HIV-1 epidemiology, including prevalence and incidence; (2) PrEP effectiveness; and (3) prevalence of oral PrEP use among key populations. The review was limited to high-income countries [18], excluding the US and Africa, in order to assess HIV-1 epidemiology and PrEP use within a comparable context of policy and economic resources.

## METHODS

This systematic literature review and meta-analysis was conducted in accordance with the Preferred Reporting Items for Systematic Reviews and Meta-Analysis (PRISMA) statement [19] to identify real-world sources reporting data on 3 outcomes in MSM, PWID, people in prison, sex workers, transgender women, and transgender men: HIV-1 prevalence and incidence; PrEP effectiveness; and prevalence of PrEP use. HIV-1 infection was identified through self-report, lab tests, healthcare records, or surveillance. Pre-exposure prophylaxis effectiveness was measured by HIV-1 prevalence or incidence among PrEP users, using HIV-1 infections identified at the time of data collection or throughout a study period, depending on the study design. PrEP use was identified through self-report, biologic sampling, study staff observations, or review of healthcare records. Included high-income countries and independent economies were defined by the World Bank country classifications [18].

### Search Strategy

Three separate searches of Embase, PubMed, and the Cochrane Library for peer-reviewed, real-world literature, published in English, were performed in July 2023. HIV-1 epidemiology searches were constrained to sources published between 1 January 2019 and 15 July 2023; this search start date was selected because HIV-1 seroprevalence in Europe from 2009 to 2019 was previously published [20]. PrEP effectiveness and oral PrEP use were constrained to sources published between 1 January 2017 and 15 July 2023, to capture data published after the approval of oral PrEP in Europe in July 2016 [21]. Search terms are summarized in Supplementary Tables 1a–3c.

In addition, gray literature searches, not limited to publications in English, were performed in July 2023 for congress abstracts published between January 2017 and July 2023, and in November 2023 for local and international sources. Local sources included local public health department websites and local HIV research and educational institutions. International sources included international public health body websites, major national public health websites, and HIV advocacy/research organizations. Search results for each congress are summarized in Supplementary Table 4a, and results for local and international sources are summarized in Supplementary Tables 4b and 4c.

### Screening and Data Extraction

For each search, database results were imported into an EndNote library and deduplicated using a modification of a previously published method [22]. The remaining titles were imported into Covidence, a web-based tool for managing systematic reviews, article screening, and data extraction [23]. Titles and abstracts of articles were independently screened for inclusion by 2 reviewers, based on predetermined intervention, context, outcomes, and study design elements (Supplementary Table 5), with discrepancies resolved by a third reviewer. Data from randomized clinical trials were excluded, as were case reports, and single-site or clinic-level sources, due to the potential for bias related to site-specific factors. Sources without primary data and sources with counterfactual data (ie, mathematical modeling reports, meta-analyses, systematic reviews, opinion pieces/reviews, letters, or comments) were also excluded. Full-text articles selected from the title and abstract screening were retrieved and reviewed to assess their eligibility by 2 independent reviewers.

Titles of gray literature were evaluated for relevance. Full text of records selected as potentially relevant were retrieved and reviewed according to the inclusion/exclusion criteria outlined in Supplementary Table 5.

Data were extracted from eligible sources identified in the 3 searches into each outcome category by a reviewer and verified by a second independent reviewer [24]. HIV-1 prevalence and incidence data, regardless of PrEP use, were extracted as HIV-1 epidemiology; HIV-1 prevalence and incidence data among PrEP users were extracted as PrEP effectiveness; and the percentage of PrEP use in key populations was extracted as prevalence of PrEP use.

### Quality Assessment

Quality of included sources was assessed independently by one reviewer and verified by a second independent reviewer using a modified Joanna Briggs Institute's critical appraisal tool shown in Supplementary Tables 6a and 6b. This tool was designed to determine the extent to which a study reporting prevalence or incidence addressed the possibility of bias in its design, conduct, and analysis [25]. The checklist was modified to ask specific questions relevant to HIV-1 or PrEP use.

### Meta-Analyses

HIV-1 prevalence, HIV-1 incidence, and PrEP use data were pooled by high-income country or region, by key population, and by PrEP use status, where available. Simple or pooled (if ≥2 sources were included) HIV-1 prevalence or incidence rates and 95% confidence intervals (CIs) were calculated in Excel using the meta-analysis add-in MetaXL (version 5.3) [26]. For sources not directly reporting prevalence or incidence, proportions or rates were calculated using data available within the source (ie, number of HIV-1 cases or PrEP users, the size of key populations, or the total person-time). Prevalence or incidence estimates were pooled via a random-effects model that also calculated the variability within and between sources. For sources reporting data from multiple time points, data were combined across years. Sources reporting data for individuals in 2 or more key population groups were analyzed in each respective group, and as a separate group of individuals from multiple key populations. Further analyses were stratified by data year (before 2020 in or after 2020), participant characteristics (ie, PrEP eligibility, age, migrant status), and study quality score (high quality, ≥80%; moderate-to-low quality, <80%). Heterogeneity across sources was evaluated by Cochran's Q test and the I^2^ statistic, where I^2^ values ≥75% indicate high heterogeneity across sources in a meta-analysis [27].

For each key population, estimates of each sociodemographic characteristic, including sex, age, race/ethnicity, educational level, employment, and immigration status, were pooled using a random-effects meta-analysis.

## RESULTS

**Figure 1.**
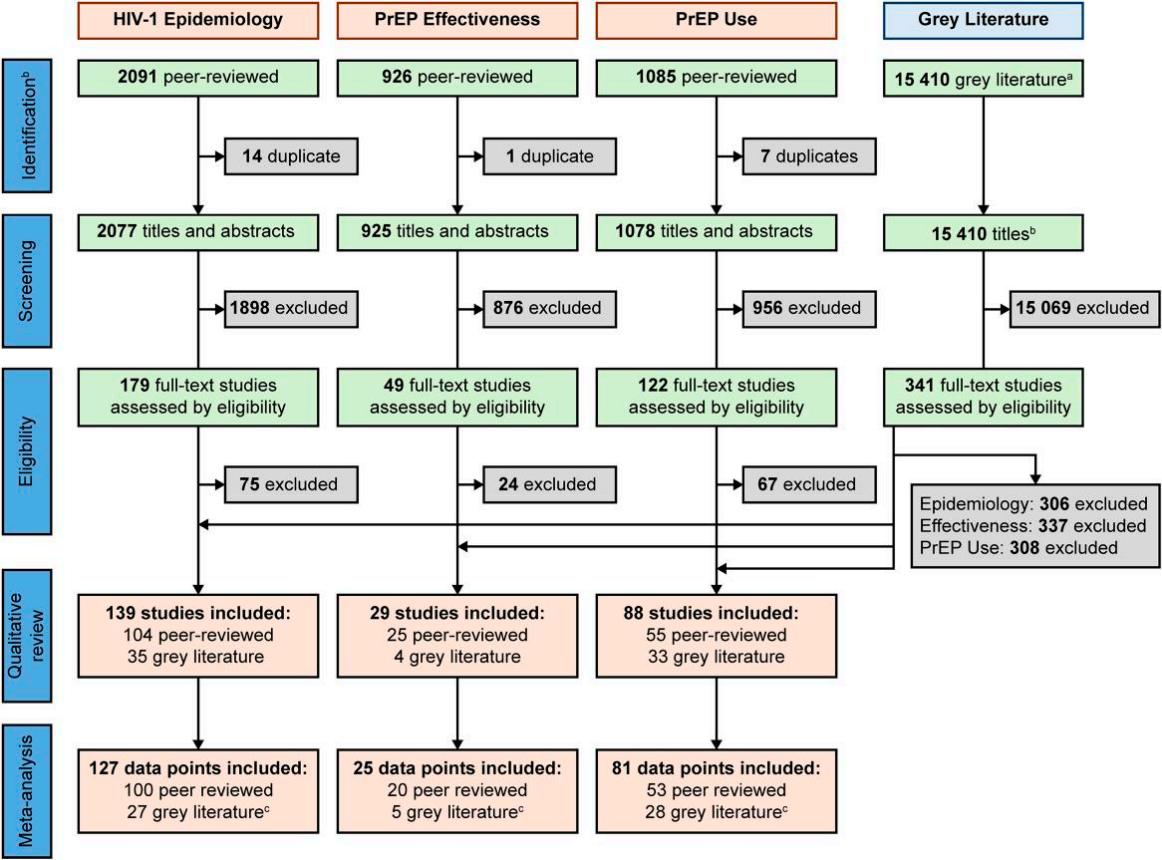
PRISMA diagram of study selection. ^a^Gray literature includes local and international sources; identification and screening were conducted for all 3 objectives simultaneously when searching the gray literature. ^b^Duplicates identified in Covidence; for peer-reviewed records, all identified articles from the database searches were deduplicated in Endnote before importing into Covidence for review. ^c^One gray literature source may contain more than one data point. PrEP, pre-exposure prophylaxis.

The combined searches captured a total of 4080 unique peer-reviewed sources and 15 410 gray literature sources. After review, a total of 204 unique sources were selected, of which 139 contained HIV-1 epidemiologic data, 29 contained PrEP effectiveness data, and 88 contained PrEP use data (Figure 1 and Table 1). Overall, this review comprised data from 38 high-income economies (36 countries, Hong Kong, and Taiwan). The majority of the sources (n = 129) recruited participants through non-probability sampling, with most regional data sourced from densely populated urban areas. The meta-analyses incorporated 127 data points for HIV-1 epidemiology, 25 for PrEP effectiveness, and 81 for prevalence of PrEP use, extracted from the combined search.

**Table 1.** Number of Sources Included in HIV-1 Epidemiology, PrEP Effectiveness, and PrEP Use Meta-analyses

High-income Country/Region	HIV-1 Epidemiology											PrEP Effectiveness^a^		Prevalence of PrEP Use
	*Prevalence*								*Incidence*			*Prev.*	*Inc.*	
	All KP	MSM	PWID	PIP	SW	TGW	TGM	MKP	All KP	MSM	PWID	PrEP users	PrEP users	
Overall	127	72	21	8	9	5	3	6	23	11	1	25	11	81
Australia	13	9	1	…	1	1	1	1	4	1	…	4	3	15
Austria	1	…	1	…	…	…	…	…	…	…	…	…	…	…
Belgium	3	1	1	…	…	…	…	…	1	…	…	1	1	3
Canada	19	12	4	3	…	…	…	1	2	2	…	1	…	11
Chile	2	2	…	…	…	…	…	…	…	…	…	…	…	…
Croatia	5	3	2	…	…	…	…	…	…	…	…	…	…	1
Cyprus	1	…	1	…	…	…	…	…	…	…	…	…	…	…
Czechia	1	…	1	…	…	…	…	…	…	…	…	…	…	…
Denmark	1	1	…	…	…	…	…	…	…	…	…	…	…	1
Estonia	3	1	1	1	…	…	…	…	…	…	…	…	…	…
Finland	1	1	…	…	…	…	…	…	…	…	…	…	…	…
France	12	8	1	1	…	…	…	…	2	…	…	2	2	13
Germany	8	3	1	…	…	…	…	…	2	…	…	4	2	6
Greece	5	1	4	…	…	…	…	…	1	…	1	…	…	…
Hong Kong	2	2	…	…	…	…	…	…	…	…	…	…	…	2
Hungary	1	…	1	…	…	…	…	…	…	…	…	…	…	…
Iceland	1	1	…	…	…	…	…	…	…	…	…	…	…	…
Ireland	3	3	…	…	…	…	…	…	…	…	…	…	…	1
Italy	4	1	…	1	1	1	…	1	…	…	…	…	…	1
Japan	1	1	…	…	…	…	…	…	…	…	…	…	…	1
Kuwait	1	…	1	…	…	…	…	…	…	…	…	…	…	…
Latvia	1	…	1	…	…	…	…	…	…	…	…	…	…	…
Lithuania	1	…	1	…	…	…	…	…	…	…	…	…	…	…
Netherlands	13	8	1	…	1	2	1	1	2	1	…	5	1	10
New Zealand	2	2	…	…	…	…	…	…	…	…	…	…	…	…
Norway	1	1	…	…	…	…	…	…	…	…	…	…	…	…
Poland	2	…	2	…	…	…	…	…	…	…	…	…	…	…
Portugal	5	3	1	1	…	…	…	…	…	…	…	…	…	3
Romania	1	…	1	…	…	…	…	…	…	…	…	…	…	…
Russia	…	…	…	…	…	…	…	…	…	…	…	…	…	1
Singapore	…	…	…	…	…	…	…	…	…	…	…	…	…	1
South Korea	1	…	…	1	…	…	…	…	…	…	…	…	…	…
Spain	16	8	2	2	4	…	…	1	3	1	…	2	…	2
Sweden	6	2	3	1	…	…	…	…	…	…	…	…	…	3
Switzerland	4	2	…	…	2	…	…	1	1	1	…	…	…	3
Taiwan	4	4	…	…	…	…	…	…	…	…	…	…	…	…
Ukraine	…	…	…	…	…	…	…	…	…	…	…	…	…	1
UK	17	9	4	…	…	1	1	…	5	5	…	6	2	14
Locations with data	35	25	22	8	5	4	3	6	10	6	1	8	6	20

Abbreviations: Inc, incidence; KP, key population; MKP, members of multiple key populations; MSM, men who have sex with men; PIP, people in prison; PrEP, pre-exposure prophylaxis; prev, prevalence; PWID, people who inject drugs; SW, sex workers; TGM, transgender men; TGW, transgender women.

^a^PrEP effectiveness was defined as the HIV-1 prevalence in known PrEP users.

### Sociodemographic Data in Key Populations

The majority of individuals across all sources, and in MSM, PWID, and PrEP users, respectively, were males (97.7%, 100%, 74.2%, 96.2%), younger than 40 years of age (71.4%, 70.2%, 50.4%, 92.9%), White (71.3%, 71.2%, 64.8%, 85.0%), and had a post-secondary degree or higher (60.4%, 68.1%, 8.8%, 55.0%); over a quarter of all individuals were born outside the study region (29.4%, 22.1%, 7.3%, 30.9%) (Supplementary Table 7). These comparisons should be viewed with caution, however, as individual sociodemographic variables were most often reported for MSM, with fewer sources reporting in other key populations and PrEP users.

### HIV-1 Epidemiology Sources

All epidemiology sources (n = 139) reported HIV-1 prevalence in at least one key population from a total of 35 countries (Supplementary Table 8a). More than half of sources were cross-sectional studies (n = 76), 50 were cohort studies (n = 35 prospective and n = 15 retrospective), 10 were from a national registry or surveillance database, and 3 studies did not report study design. Countries with the highest number of sources reporting HIV-1 prevalence included Canada (n = 25), followed by the UK (n = 23), Spain (n = 22), Australia, and the Netherlands (n = 21 each). Many sources reported HIV-1 prevalence in MSM (72 sources) and in PWID (21 sources) (Table 1, Supplementary Table 8a).

HIV-1 incidence was reported in 27 sources from 10 countries with at least one key population. HIV-1 incidence rates were most often reported in the UK (n = 5), Australia (n = 5), Canada (n = 4), and Spain (n = 3), and were primarily in MSM (n = 10), with one source reporting in PWID and 16 sources not reporting a specific key population (Table 1, Supplementary Table 8b).

### Global Pooled HIV-1 Epidemiology Estimates in Key Populations

**Figure 2.**
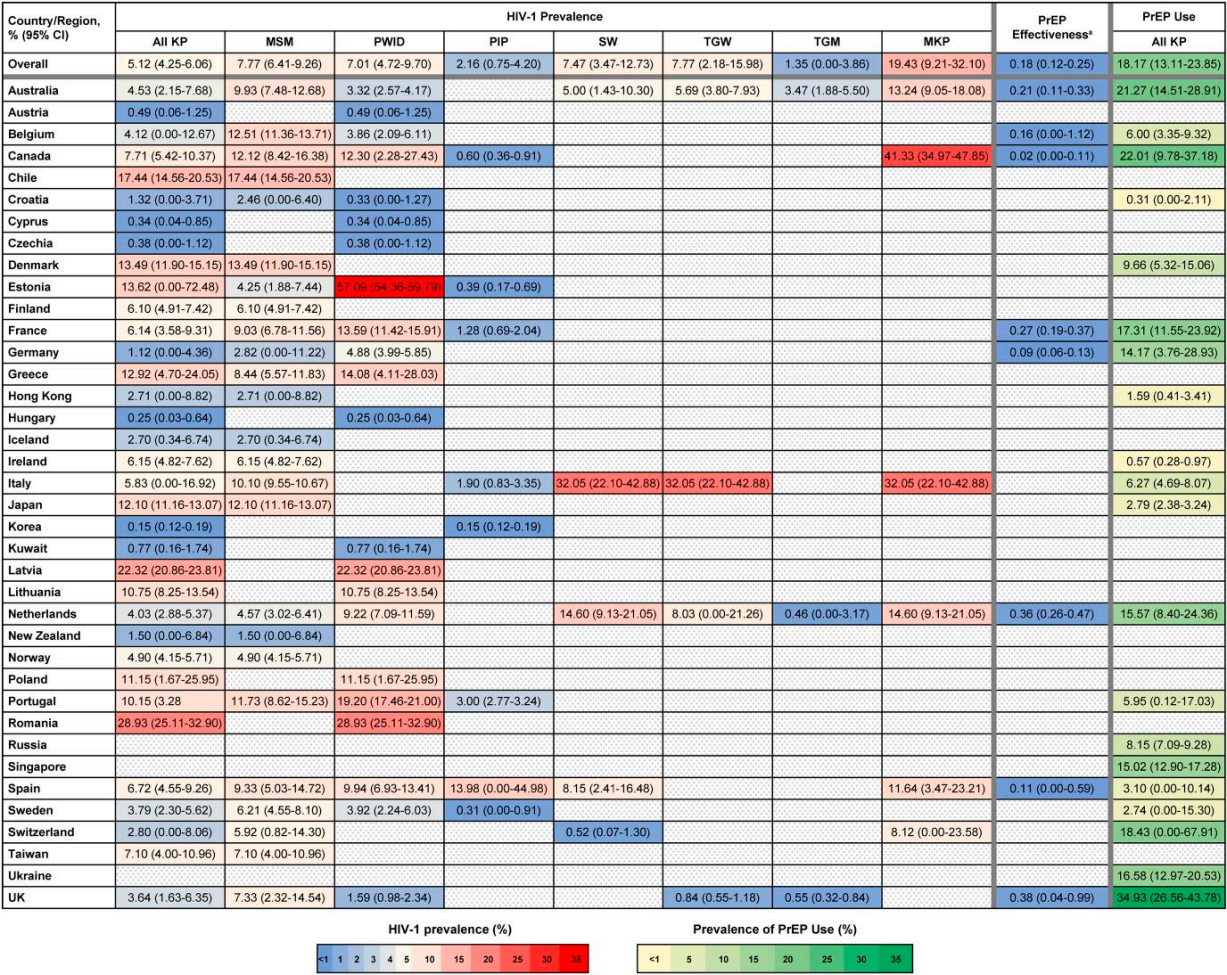
Heatmap of pooled HIV-1 prevalence in key populations, PrEP effectiveness, and of PrEP use. ^a^PrEP effectiveness was defined as the HIV-1 prevalence in known PrEP users. CI, confidence interval; MKP, members of multiple key populations; KP, key population; MSM, men who have sex with men; PIP, people in prison; PrEP, pre-exposure prophylaxis; PWID, people who inject drugs; SW, sex workers; TGM, transgender men; TGW, transgender women.

In total, 2 139 138 individuals were included in the meta-analyses for HIV-1 prevalence across all key populations and countries/regions. The global pooled estimate of HIV-1 prevalence across key populations was 5.1% (95% CI: 4.2%–6.1%) overall (n = 127 sources), and 6.7% (95% CI: 5.6%–7.8%) when excluding sources with PrEP users (n = 108 sources). The country-level median pooled prevalence in key populations was 4.9% (95% CI: 4.5%–9.1%), with the highest prevalence reported in Romania (28.9%), Latvia (22.3%), and Chile (17.4%). Countries reporting HIV-1 prevalence <1% in key populations included South Korea, Hungary, Cyprus, Czechia, Austria, and Kuwait (Figure 2).

For HIV-1 incidence, 569 076 person–years (PY) across all key populations and countries/regions were included in the meta-analyses. The global pooled estimate of HIV-1 incidence rate per 100 PY was 0.39 (95% CI: 0.26–0.58) overall (n = 23 sources) and 0.74 per 100 PY (95% CI: 0.46–1.19) when excluding sources in PrEP users (n = 14 sources). The highest overall HIV-1 incidence per 100 PY in key populations was reported in Greece (1.94 [95% CI: 1.50–2.52]), followed by Spain (0.85 [95% CI: 0.19–3.69]), with Germany reporting the lowest incidence (0.08 [95% CI: 0.04–0.16]; Table 2).

**Table 2.** Simple or Pooled Estimates of HIV-1 Incidence Rates (per 100 PY) and PrEP Effectiveness by Economy and Key Population

High-income Country	HIV Incidence Rate (95% CI), 2019–2023			PrEP Effectiveness^a^ (95% CI), 2017–2023
	All KP	MSM	PWID	All KP
Overall	0.39 (0.26–0.58)	0.71 (0.42–1.20)	1.94 (1.50–2.52)	0.15 (0.10–0.22)
Australia	0.15 (0.06–0.35)	0.40 (0.38–0.42)	…	0.11 (0.06–0.19)
Belgium	0.16 (0.01–2.51)	…	…	0.16 (0.01–2.51)
Canada	0.59 (0.28–1.25)	0.59 (0.28–1.25)	…	…
France	0.16 (0.10–0.27)	…	…	0.16 (0.10–0.27)
Germany	0.08 (0.04–0.16)	…	…	0.08 (0.04–0.16)
Greece	1.94 (1.50–2.52)	…	1.94 (1.50–2.52)	…
Netherlands	0.63 (0.25–1.60)	0.85 (0.63–1.15)	…	0.30 (0.07–1.19)
Spain	0.85 (0.19–3.69)	2.11 (1.93–2.30)	…	…
Switzerland	0.30 (0.04–2.13)	0.30 (0.04–2.13)	…	…
UK	0.71 (0.28–1.83)	0.72 (0.28–1.86)	…	0.49 (0.06–3.86)

Abbreviations: CI, confidence interval; KP, key populations; MSM, men who have sex with men; PrEP, pre-exposure prophylaxis; PWID, people who inject drugs; PY, person-year.

^a^PrEP effectiveness defined as the HIV-1 incidence in PrEP users.

### HIV-1 Epidemiology in Key Populations

Many sources reporting prevalence data in MSM were from Canada (n = 12), Australia (n = 9), and the UK (n = 9), with no sources in MSM from countries in Eastern Europe (Table 1). Prevalence data in PWID were most often reported in Canada, Greece, and the UK (n = 4 sources for each), and almost all sources collected data before 2020 (n = 19 sources).

The simple or pooled HIV-1 prevalence was the highest in members of multiple key populations (19.4%) and lowest in people in prison (2.2%) and transgender men (1.4%), with similar prevalences reported in MSM, PWID, sex workers, and transgender women (7.0%–7.8%) (Figure 2). Members of multiple key populations included those who were sex workers and transgender women (n = 3 sources), PWID and MSM (n = 3 sources), and PWID and sex workers (n = 2 sources).

Prevalence in MSM was lowest (<3%) in Croatia, Germany, Hong Kong, Iceland, and New Zealand, and highest in Chile (17.4%) and Denmark (13.5%) (Figure 2). Prevalence in PWID was <1% in Austria, Croatia, Cyprus, Czechia, Hungary, and Kuwait, high in Latvia (22.3%) and Romania (28.9%), and highest in Estonia (57.1%), the latter value coming from a single study [28]. In people in prison, HIV-1 prevalence was ≤3.0% in all countries with available data except for Spain (14.0%). Spain's prevalence was based on UNAIDS data collected in Catalonia between 2002 and 2016 [29]. In sex workers, prevalence was highest in Italy (32.1%), followed by the Netherlands (14.6%), and was lowest in Switzerland (0.5%). Prevalence in transgender women was also highest in Italy (32.1%) and the Netherlands (8.0%) and was lowest in the UK (0.8%). Prevalence in transgender men was 3.5% in Australia and <0.6% in the UK and the Netherlands. Prevalence was consistently high in members of multiple key populations, ranging from 8.1% (Switzerland) to 41.3% (Canada).

The overall pooled HIV-1 prevalence in individuals born locally and in those born outside the country was 7.2% (95% CI: 3.5%–12.0%) and 8.7% (95% CI: 2.6%–17.4%), respectively (n = 20 sources) and in MSM was 9.0% (95% CI: 5.9%–12.7%) and 10.7% (95% CI: 5.4%–17.4%), respectively (n = 10 sources). Global pooled HIV-1 prevalence was 5.6% (95% CI: 4.6%–6.6%) prior to 2020 (n = 104 sources), and was 4.7% (95% CI: 2.7%–7.3%) during or after 2020 (n = 13 sources). Prevalence pre-2020 and during or post-2020 in MSM was 8.3% (95% CI: 6.9%–9.9%, n = 62 sources) and 7.7% (95% CI: 2.1%–15.8%, n = 9 sources), respectively and, in people in prison, was 2.5% (95% CI: 0.4%–5.9%, n = 6 sources) and 1.2% (95% CI: 0%–5.3%, n = 2 sources), respectively. Global pooled HIV-1 prevalence was 1.9% (95% CI: 1.1%–2.8%) in individuals aged <40 years, and 5.5% (95% CI, 0.8%–13.3%) in those aged ≥40 years (n = 18 sources) and 2.5% (95% CI, 1.1%–4.3%) and 8.6% (95% CI, 0.3%–23.8%), respectively, in MSM (n = 12 sources).

Key populations with the highest incidence rates per 100 PY included MSM in Spain (2.11) and in PWID in Greece (1.94), with the lowest incidence rates observed in MSM in Switzerland (0.30) and in Australia (0.40) (Table 2).

### PrEP Effectiveness

PrEP effectiveness in key populations was assessed as the prevalence (n = 29 sources) and the incidence rate (n = 11 sources) of HIV-1 in individuals known to be using PrEP. The 29 effectiveness sources included 25 cohort studies (n = 19 prospective and n = 6 retrospective), 2 cross-sectional studies, one nested case-control study, and one study from a national registry or surveillance database. Prevalence among PrEP users was reported in 8 countries, with most sources from the UK (n = 6), the Netherlands (n = 5), Australia (n = 4), and Germany (n = 4). Incidence among PrEP users was reported in 6 countries, with most sources from Australia (n = 3) (Table 1, Supplementary Table 8c).

Global pooled HIV-1 prevalence among PrEP users was 0.2% (95% CI: 0.1%–0.3%; n = 25 sources; Figure 2) compared with 6.7% (95% CI: 5.6%–7.8%; n = 108 sources) in key populations excluding PrEP users. Notably, HIV-1 prevalence in PrEP users was among the lowest in Canada and Spain (0.0% and 0.1%, respectively), despite a high overall prevalence of HIV-1 in key populations within each country (7.7% and 6.7%, respectively).

Global pooled HIV-1 incidence per 100 PY was lower among PrEP users (0.15; 95% CI: 0.10–0.22; n = 11 sources) compared with key populations excluding PrEP users (0.74; 95% CI: 0.46–1.19; n = 11 sources; Table 2). Among the included HIV-1 effectiveness sources, 13 reported zero new HIV-1 diagnoses among PrEP users, and 6 reported that new diagnoses occurred during periods with non-optimal adherence or after PrEP discontinuation.

### Prevalence of PrEP Use

Overall, 88 sources reported on oral PrEP use in key populations from 20 countries; of these, 59 were cross-sectional, 21 were cohort studies (n = 18 prospective and n = 3 retrospective), 6 were from a national registry/database, and 2 studies did not specify the study design (Supplementary Table 8c).

Data points from 81 sources were included in the meta-analysis and were most often from Australia (n = 15), the UK (n = 14), France (n = 13), and Canada (n = 11) (Table 1). Thirteen sources specified that participants were PrEP-eligible individuals (5 in MSM or transgender women, one in PWID, 7 sources did not define the included key population). Most sources did not differentiate between daily and on-demand PrEP use (n = 75), with 6 sources on daily PrEP use only. Twenty-eight sources reported current PrEP use, while the rest included both concurrent and historical use. Overall, 71 data points were in MSM, 3 in transgender women, 2 in transgender men, one in PWID and 4 in other people who may need or want PrEP.

Global prevalence of oral PrEP use among all key populations was 18.2% (95% CI: 13.1%–23.9%), with a country-level median of 8.9% (95% CI: 6.9–15.4%). PrEP use was lowest in Croatia, Ireland, Hong Kong, Sweden, and Japan (<3.0% for each), and highest in the UK (34.9%), Canada (22.0%), and Australia (21.3%) (Figure 2). The point estimate of PrEP use increased from 15.9% (95% CI: 12.5%–19.6%; n = 62) before 2020% to 29.2% (95% CI: 13.5%–47.7%; n = 13) in or after 2020, although CIs overlapped. Estimates of PrEP use were higher among individuals specified as PrEP-eligible (43.4%; 95% CI: 28.8%–58.6%) than globally across all key populations (18.2%; 95% CI: 13.1–23.9).

**Figure 3.**
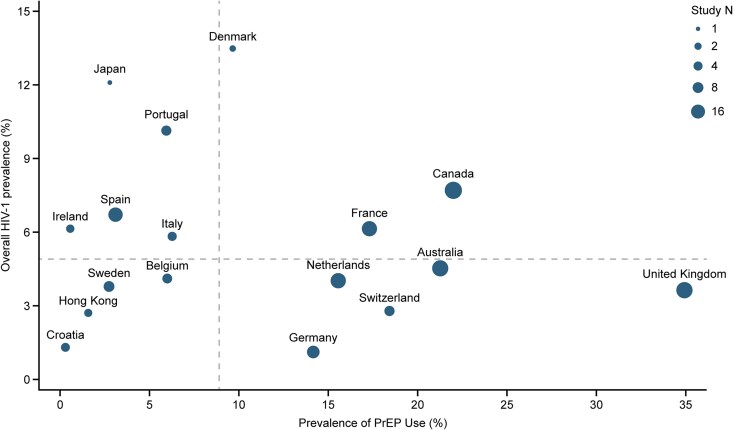
Pooled estimates of overall prevalence of HIV-1 and PrEP use across all key populations, by country/region. Out of a total of 38 high-income economies eligible for meta-analysis of the SLR, 17 economies with data available for both PrEP prevalence and HIV-1 use in key populations were plotted. Marker size indicates the combined number of HIV-1 and PrEP use sources for each country or region and marker position represents pooled meta-analysis estimates for both statistics. The dashed lines represent the country-level pooled estimated median overall HIV-1 prevalence (4.9% [95% CI: 4.5%–9.1%] for 35 countries) and median prevalence of PrEP use (8.9% [95% CI: 6.9%–15.4%] for 20 economies). CI, confidence interval; PrEP, pre-exposure prophylaxis; SLR, systematic literature review.

A comparison of the prevalence of HIV-1 and of oral PrEP use in key populations from 17 regions with available data is shown in Figure 3. Sources included in the pooled estimates of HIV-1 prevalence and PrEP use across key populations were most often from Canada (n = 28), the UK (n = 26), and Australia (n = 25). Prevalence of oral PrEP use ranged from 0.3%–32.2% in countries/regions with an HIV-1 prevalence below the country-level median pooled prevalence (4.9%) and from 2.8%–23.6% in countries with a prevalence above the median.

### Study Quality

The median quality score (range) was 64 (18–100) for sources reporting HIV-1 prevalence, 82 (27–100) for HIV-1 incidence, and 55 (27–91) for PrEP use (Supplementary Tables 8a–8c). HIV-1 prevalence was lower in high-quality sources (2.4% [95% CI: 1.3%–3.8%], n = 32) than in moderate-to-low-quality sources (6.2% [95% CI: 5.1%–7.4%], n = 95). Overall HIV-1 incidence rates and global pooled HIV-1 prevalence and incidence rates among PrEP users were similar regardless of study quality. Estimates of PrEP use were higher in high-quality studies (48.8% [95% CI: 30.2%–67.5%]) versus moderate-to-low-quality studies (15.9% [11.7%–20.5%]).

### Heterogeneity

I² statistics were high overall (>99.8%) for HIV-1 prevalence, HIV-1 incidence, and PrEP use, indicating considerable variation across regions and sources. Moderate-to-considerable heterogeneity was observed in PrEP effectiveness (71.9% for prevalence and 65.3% for incidence; Supplementary Table 9).

## DISCUSSION

This systematic literature review of HIV-1 epidemiology, PrEP effectiveness, and prevalence of oral PrEP use found considerable variation across key populations most affected by HIV-1 and high-income economies outside of the US and Africa. Sources were most often in the UK and Canada, with the majority of sources reporting in MSM. HIV-1 prevalence was highest in members of multiple key populations, MSM, and transgender women, and ranged from 0.2% in South Korea to 28.9% in Romania. PrEP was highly effective in these real-world settings, with lower HIV-1 prevalence rates reported in PrEP users (0.2%) compared with the overall population (6.7%). However, despite its effectiveness, oral PrEP use remained low (18.2%) among key populations.

National HIV-1 prevention strategies in high-income economies have historically focused heavily on MSM, which was reflected in the high number of sources identified for this population. Pooled estimates for HIV-1 prevalence in MSM were lower in the current study than in a study by Stengaard et al. (2021) for 7 of 9 overlapping countries, and appeared lower since 2020 than prior to 2020, though there was insufficient precision to demonstrate a significant change in HIV-1 prevalence over time [20]. Despite this possible overall decline, prevalence in MSM remained high (>10.0%) in some countries, particularly across Western Europe (Belgium, Denmark, Italy, and Portugal), Canada, Chile, and Japan, suggesting the need for additional or renewed HIV-1 reduction efforts targeting MSM in these regions.

Similar to in MSM, HIV-1 prevalence in people in prison appeared to decline somewhat from pre-2020 to post-2020, but remained high, likely due to the many factors present in prison environments associated with increased risk of HIV-1 acquisition, such as the high prevalence of injection drug use and condomless receptive sex [30–32]. While Canada initiated a country-wide prison needle exchange program in 2018 [12, 33, 34], similar national harm reduction programs are not widespread, and few prisons offer PrEP to incarcerated people despite being identified as an intervention of essential health impact by the World Health Organization [12, 33–35]. However, data on incarcerated people are limited and further research is needed to better understand HIV-1 epidemiology in this population.

High HIV-1 prevalence (>9%) among PWID was observed in countries with both high and low rates of injection drug use, such as the Baltic states, France, and Canada (high-use) and Romania, Greece, and the Netherlands (low-use) [36, 37]. Wide differences in HIV-1 prevalence among PWID in neighboring European countries (eg, Belgium and France, Hungary and Romania) revealed strong regional variation, likely due to differences in past localized outbreaks, such as in Greece and Romania [38, 39], and the timing, composition, and effectiveness of harm-reduction strategies implemented by each country. These findings align with the overall geographic variation in HIV-1 prevalence observed by Degenhardt et al. (2017) in PWID [40]. Recent and ongoing HIV-1 outbreaks among PWID support persistent and possibly rising rates of HIV-1 in these key populations [41, 42]. These results support the need for expanded harm reduction strategies, including needle and syringe exchange programs, in PWID.

The current review found very few sources for HIV-1 prevalence in transgender people, despite the high prevalence in transgender women observed here (7.8%) and in other published literature (19.9%) [43–45]. Transgender individuals more often experience multiple socioeconomic factors associated with acquiring HIV-1, including high rates of unemployment, homelessness, drug use, migrant status, incarceration, and sex work [43, 46, 47]. In the current study, 4 sources reported that transgender individuals were also often migrants and involved in sex work [48–51]. Despite the high prevalence of HIV-1 and multiple risk factors in transgender individuals, few sources have been published in this population, highlighting a need for further investigation in this key population.

While this review did not specifically target migrant individuals as a key population, HIV-1 prevalence appeared to be slightly higher in foreign-born individuals (8.7%) than in locally-born individuals (7.2%), though interpretation is limited due to wide and overlapping CIs. In addition, European Union (EU) countries with higher HIV-1 prevalence rates were often countries with a higher proportion of non-EU migrants (eg, countries in southern Europe and the Baltic states), with the exception of Germany, which has a large migrant population and low HIV-1 prevalence [52]. In 2023, migrants accounted for 9% of all people in Europe but 48% of all new diagnoses of HIV-1 [52–54]. Migrant individuals may experience multiple factors that increase their risk of HIV-1 acquisition, such as unemployment, socio-economic instability, language barriers, and limited access to PrEP [55]. In 2008, migrant individuals comprised 65% of sex workers in Western Europe and 20% of people in prison had foreign citizenship in the reporting country [54, 56]. An estimated 30% of migrants acquire HIV-1 post-migration, further underscoring the need for HIV-1 prevention policies and strategies tailored to migrant populations [57].

The high effectiveness of oral PrEP in real-world settings was demonstrated by the low pooled HIV-1 prevalence and incidence rates in known oral PrEP users versus other individuals, consistent with previously published studies [43, 58]. However, despite the effectiveness of PrEP, the observed overall prevalence of oral PrEP use among key populations was low (18%). PrEP use increased from 16% prior to 2020 to 29% subsequently, suggesting that global initiatives to improve PrEP accessibility are working, although levels still remain lower than reported in key populations in the US in 2022 (36%) [59]. PrEP use varied considerably across the 20 included high-income countries, from 34.9% in the UK to <0.6% in Croatia and Ireland.

PrEP utilization is determined by complex array of individual, social, and structural factors, such as medical mistrust, self-perceived risk, provider knowledge, stigma, and cost. Local health policies play a key role in shaping these dynamics [14]. A comparison of national PrEP strategies among countries with higher and lower estimated prevalences of PrEP use revealed a number of differences impacting PrEP accessibility. Countries with higher estimated PrEP use often had broad PrEP strategies that expanded eligibility beyond MSM, had clear and easy-to-find online resources, and allowed online purchasing of PrEP [58, 60–64]. Conversely, countries with lower estimated PrEP use often limited PrEP eligibility to MSM and transgender people, restricted providers to HIV specialists, and offered PrEP at a limited number of locations (eg, one site in Croatia) [58, 60–64]. PrEP costs in low-uptake countries were often high, for example in Italy where one month of PrEP cost 60€ in 2020, or were difficult to predict, for example in Ireland where individuals eligible for government assistance in paying for PrEP often experience roadblocks when trying to access it [62]. It is important to note, however, that the majority of the sources used to estimate PrEP prevalence in this study were in MSM (87.7%) from just 5 countries (77.8%). Further research is needed to assess PrEP use more broadly in key populations across high-income economies.

This is the first study to comprehensively report real-world evidence on HIV-1 epidemiology, PrEP effectiveness, and PrEP use in underrepresented geographies and populations using established and rigorous methodologies. However, a number of limitations may affect the robustness of cross-economy comparisons and reduce the generalizability of the findings. First, economies and key populations were unequally represented, leading to data gaps and disproportionate estimates in subsequent meta-analyses. Single-site sources, which could provide additional information about regional trends, were excluded from the meta-analysis based on methodological concerns. Second, estimates of PrEP coverage were limited to oral PrEP use and should not be interpreted as estimates of overall HIV prevention coverage as described in the UNAIDS 2030 HIV prevention target. Third, although the search strategies were designed to be inclusive of any studies reporting the proportion of oral PrEP users in key populations, additional relevant sources may not have been captured due to the wide range of reporting practices used to describe PrEP prevalence. Fourth, an important source of heterogeneity was the aggregation of data collected across multiple years into a single pooled estimate. While this approach was chosen to increase statistical power and provide a broader overview of HIV and PrEP use, it may obscure meaningful temporal variation, particularly during periods of rapid changes in HIV epidemiology and the evolving landscape of HIV prevention.

## CONCLUSION

This systematic literature review provides a comprehensive review of HIV-1 epidemiology, PrEP effectiveness, and PrEP use data across key populations in high-income economies. The results demonstrate that estimates of HIV-1 prevalence and PrEP use vary across key populations and regions, and that despite overall high PrEP effectiveness, PrEP use remains relatively low. Therefore, it is important that efforts are focused on targeting strategies to provide equitable access to PrEP to strengthen local and national HIV-1 prevention initiatives. There is also a need for the establishment of more precise estimates of denominators to better and more holistically assess HIV-1 epidemiology and prevention in all key populations.

## Supplementary Material

ofaf785_Supplementary_Data

## Supplementary Data

Supplementary materials are available at *Open Forum Infectious Diseases* online. Consisting of data provided by the authors to benefit the reader, the posted materials are not copyedited and are the sole responsibility of the authors, so questions or comments should be addressed to the corresponding author.
