# Supplementary material for: HIV-1 Prevalence and Oral Pre-Exposure Prophylaxis Effectiveness and Prevalence of Use Among Key Populations in High-Income Economies (2017–2023): A Systematic Review and Meta-Analysis of Real-World Studies
Source: Open Forum Infect Dis. 2026 Jan 22;13(1):ofaf785. doi: 10.1093/ofid/ofaf785 (PMC12824944; doi:10.1093/ofid/ofaf785)
Supplement: ofaf785_Supplementary_Data [file ofaf785_supplementary_data.docx]

**Supplement to HIV-1 Prevalence and Pre-Exposure Prophylaxis Effectiveness and Prevalence Among Key Populations in High-Income Economies (2017–2023): A Systematic Review and Meta-Analysis of Real-World Studies**

[**Supplementary Table 1a. Search Terms for HIV-1 Epidemiology Sources (Embase)** 2](#_Toc209192759)

[**Supplementary Table 1b. Search Terms for the HIV-1 Epidemiology Sources (PubMed)** 8](#_Toc209192760)

[**Supplementary Table 1c. Search Terms for the HIV-1 Epidemiology Sources (Cochrane Library)** 15](#_Toc209192761)

[**Supplementary Table 2a. Search Terms for Prevalence of PrEP Use Sources (Embase)** 18](#_Toc209192762)

[**Supplementary Table 2b. Search Terms for Prevalence of PrEP Use Sources (PubMed)** 25](#_Toc209192763)

[**Supplementary Table 2c. Search Terms for Prevalence of PrEP Use Sources (Cochrane Library)** 32](#_Toc209192764)

[**Supplementary Table 3a. Search Terms for PrEP Effectiveness Sources (Embase)** 36](#_Toc209192765)

[**Supplementary Table 3b. Search Terms for PrEP Effectiveness Sources (PubMed)** 42](#_Toc209192766)

[**Supplementary Table 3c. Search Terms for PrEP Effectiveness Sources (Cochrane Library)** 48](#_Toc209192767)

[**Supplementary Table 4a. Grey Literature Results for Hand-Searching Abstracts of Relevant Congresses** 50](#_Toc209192768)

[**Supplementary Table 4b. Search Strategies for Local Grey Literature Searches** 51](#_Toc209192769)

[**Supplementary Table 4c. Search Strategies for International Grey Literature Searches** 58](#_Toc209192770)

[**Supplementary Table 5. Inclusion and Exclusion Criteria** 61](#_Toc209192771)

[**Supplementary Table 6a. Criteria for Assessing Risk of Bias – HIV-1 Epidemiology** 62](#_Toc209192772)

[**Supplementary Table 6b. Criteria for Assessing Risk of Bias – Prevalence of PrEP Use** 64](#_Toc209192773)

[**Supplemental Table 7. Demographic Characteristics of Individuals Pooled Across Sources for all Three Outcomes** 66](#_Toc209192774)

[**Supplementary Table 8a. Overview of Unique Sources Reporting HIV-1 Prevalence and/or PrEP Effectiveness Data, Including (n=145 sources)** 67](#_Toc209192775)

[**Supplementary Table 8b. Overview of Included Sources With HIV-1 Incidence Data^a^ (n=26 sources)** 76](#_Toc209192776)

[**Supplementary Table 8c. Overview of Included Sources With PrEP Use Data (n=88 sources)** 78](#_Toc209192777)

[**Supplemental Table 9. I^2^ Statistics for Heterogeneity Among Included Sources for HIV-1 Epidemiology, PrEP Effectiveness, and PrEP Use, by Country/Region and Key Population** 83](#_Toc209192778)

[**References** 86](#_Toc209192779)

# **Supplementary Table 1a. Search Terms for HIV-1 Epidemiology Sources (Embase)**

| **Term Group** | **#** | **Search Terms** | **Number of Hits  (July 2023)** |
| --- | --- | --- | --- |
| **Epidemiology: HIV-1**  **(with proximity 'free text' terms)** | 1 | Human immunodeficiency virus infection'/dm_ep OR 'Human immunodeficiency virus 1 infection'/dm_ep OR 'human immunodeficiency virus prevalence'/exp | 49,537 |
|  | 2 | ('HIV adj1 rate' OR 'HIV adj1 incidence' OR 'HIV adj1 prevalence' OR 'HIV adj1 use' OR 'HIV adj1 utilization' OR 'HIV adj1 infection' OR 'HIV adj1 epidemiology'):ti | 0 |
|  | 3 | #1 OR #2 | 49,537 |
| **Sex Workers** | 4 | sex worker'/exp OR 'prostitution'/exp | 12,761 |
|  | 5 | ('sex work' OR 'sex work*' OR prostitute* OR prostitution OR 'call girl*' OR 'exchange sex' OR 'ladies night' OR 'sex payment' OR 'survival sex' OR 'transactional sex' OR 'walk pavement' OR nightwalker* OR prostitut* OR sex industr* OR streetwalker* OR whore*) | 23,762 |
|  | 6 | #4 OR #5 | 36,054 |
| **MSM** | 7 | men who have sex with men'/exp OR 'men who have sex with men and women'/exp OR 'homosexual male'/exp OR 'bisexual male'/exp OR 'LGBT people'/exp OR 'LGBTQIA+ people'/exp | 41,083 |
|  | 8 | ('men who have sex with men' OR 'men have sex with men' OR 'men having sex with men' OR bisexual* OR homosexual* OR MSM OR 'male to male sexual contact' OR 'men sexual relations with men' OR 'men who have sex with both men and women' OR 'non binary' OR 'non heterosexual*' OR bisexual* OR cruisin OR cruising* OR gay OR gays OR GBMSM OR GB-MSM OR homosexual* OR lesbigay* OR lgbt OR MASM OR MSM OR MSMW OR queer*) | 101,527 |
|  | 9 | #7 OR #8 | 111,889 |
| **Transgender Persons** | 10 | transgender'/exp OR 'transgender and gender nonbinary'/exp OR 'female to male transgender'/exp OR 'male to female transgender'/exp | 13,863 |
|  | 11 | ('trans female*' OR 'trans male*' OR 'trans man' OR 'trans men' OR 'trans people' OR 'trans people' OR 'trans peoples' OR 'trans person' OR 'trans wom*' OR 'transwom*' OR transman OR transmen OR transpeople OR transperson* OR 'gender reassignment' OR 'gender variant' OR 'intersex individual*' OR 'intersex people' OR 'intersex person' OR 'sexual dissident' OR 'trans sexual*' OR 'two spirit' OR glbt OR glbtq OR lgbt OR lgbtq OR lgbtqq OR transgender*) | 23,384 |
|  | 12 | #10 OR #11 | 23,447 |
| **Drug Users** | 13 | injection drug user'/exp | 4,688 |
|  | 14 | I V drug user' OR 'I V drug users' OR 'injectable drug user' OR 'injectable drug users' OR 'intravenous drug user' OR 'intra-venous drug user' OR 'intravenous drug users' OR 'intra-venous drug users' OR 'IV drug user' OR 'IV drug users' OR 'IVDU' OR 'IVDUs' OR 'IVDUs' OR 'people inject drugs' OR 'people injected drugs' OR 'people injecting drugs' OR 'people who use drugs' OR 'person inject drugs' OR 'person who uses drugs' OR 'persons injecting drugs' OR 'persons who use drugs' OR 'PWID' OR 'PWIDs' OR 'PWIDs' OR 'PWUD' OR 'PWUDs' OR 'PWUDs' OR 'injecting drug user' OR 'intravenous drug abuse' OR 'intravenous drug use' OR 'intravenous substance abuse' OR 'intravenous substance misuse' OR 'intravenous substance use' OR 'parenteral drug abuse' OR 'parenteral drug use' OR 'people who inject drugs' OR 'person who inject drugs' OR 'persons who inject drugs' OR 'injecting drug users' OR 'intravenous drug abuser' OR 'intravenous drug user' OR 'intravenous substance abuser' OR 'intravenous substance misuser' OR 'intravenous substance user' OR 'parenteral drug abuser' OR 'parenteral drug user' OR 'intravenous drug abusers' OR 'intravenous drug users' OR 'intravenous substance abusers' OR 'intravenous substance misusers' OR 'intravenous substance users' OR 'parenteral drug abusers' OR 'parenteral drug users' OR (((inject* OR intraven* OR parenteral OR use OR misus* OR user OR users) AND (drug* OR substance*)) OR ((inject* OR intraven* OR parenteral) AND (drug* OR substance*))) | 3,630,377 |
|  | 15 | #13 OR #14 | 3,630,377 |
| **Prisoner Population** | 16 | correctional facility'/exp OR 'prisoner'/exp | 33,600 |
|  | 17 | ('correctional facilities' OR 'correctional facility' OR 'correctional institution' OR 'correctional institutions' OR 'gaol' OR 'gaols' OR 'penal facilities' OR 'penal facility' OR 'penal institution' OR 'penal institutions' OR custodial OR imprison* OR incarcerat* OR inmate* OR jail OR jails OR penitentiar* OR prison* OR 'closed setting*' OR cellmate* OR confinement* OR detainee* OR detention* OR offender OR offenders OR penal OR penitentiar* OR reformator* OR remand* OR convict*) | 125,682 |
|  | 18 | #16 OR #17 | 125,914 |
| **Serodiscordant Couples** | 19 | serodiscordant couple'/exp OR (serodiscordan* OR 'sero-discordan*' OR 'mixed infection status') | 1,879 |
| **All Target Patient Populations** | 20 | #6 OR #9 OR #12 OR #15 OR #18 OR #19 | 3,863,730 |
| **Epidemiology in Target Populations** | 21 | #3 AND #20 | 18,453 |
| **Restrict to Publication On/After  1st Jan 2019** | 22 | #21 AND [2019-2023]/py | 2,956 |
| **Remove Irrelevant Publication Types - Expanded** | 23 | #22 NOT ('Review':de OR 'systematic':de OR 'meta-analysis':de OR 'meta analysis (topic)'/exp OR 'Case Reports':de OR 'comment':de OR 'Controlled study'/exp OR 'Controlled clinical trial'/exp OR 'Randomized controlled trial'/exp) | 2,019 |
| **Animal Studies** | 24 | #23 NOT ('animal cell' OR 'animal experiment' OR 'animal health' OR 'animal model' OR 'animal tissue' OR 'bird' OR 'cancer model' OR 'canine' OR 'dog' OR 'ex vivo study' OR 'goat' OR 'Hep-G2 cell line' OR 'in vitro study' OR 'knockout mouse' OR 'Kupffer cell' OR 'mammal' OR 'model' OR 'mouse' OR 'mouse model' OR 'murine' OR mice OR 'nonhuman' OR 'nude rat' OR 'rat' OR 'rat model' OR 'tumor cell' OR 'tumor model' OR 'tumor xenograft' OR 'veterinary'):ab,ti | 1,764 |
| **World Bank 'High-Income' Countries** | 25 | Andorra'/exp OR 'Antigua and Barbuda'/exp OR 'Antigua Barbuda' OR 'Aruba'/exp OR 'Asia'/exp OR 'Australia'/exp OR 'Austria'/exp OR 'Bahamas'/exp OR 'Bahrain'/exp OR 'Barbados'/exp OR 'Belgium'/exp OR 'Bermuda'/exp OR 'British Virgin Islands'/exp OR 'Brunei'/exp OR 'Canada'/exp OR 'Cayman Island*' OR 'Channel Islands'/exp OR 'Chile'/exp OR 'Cook Island*' OR 'Croatia'/exp OR 'Curacao'/exp OR 'Cyprus'/exp OR 'Czech Republic'/exp OR 'Czech Republic*' OR 'Denmark'/exp OR 'England'/exp OR 'Estonia'/exp OR 'Europe'/exp OR 'Faroe Island*' OR 'Finland'/exp OR 'France'/exp OR 'French Polynesia*' OR 'Germany'/exp OR 'Gibraltar'/exp OR 'Great Britain' OR 'Great Britain' OR 'Greece'/exp OR 'Greenland'/exp OR 'Guam'/exp OR 'Guernsey'/exp OR 'Hong Kong*' OR 'Hungary'/exp OR 'Iceland'/exp OR 'Ireland'/exp OR 'Isle of Man' OR 'Israel'/exp OR 'Italy'/exp OR 'Japan'/exp OR 'Jersey Channel Island*' OR 'Kuwait'/exp OR 'Latvia'/exp OR 'Liechtenstein'/exp OR 'Lithuania'/exp OR 'Luxembourg'/exp OR 'Macau'/exp OR 'Malta'/exp OR 'Monaco'/exp OR 'Netherlands'/exp OR 'New Caledonia'/exp OR 'New Zealand'/exp OR 'New Zealand*' OR 'Northern Mariana Island*' OR 'Norway'/exp OR 'Oman'/exp OR 'Panama'/exp OR 'Poland'/exp OR 'Polynesia'/exp OR 'Portugal'/exp OR 'Puerto Rico'/exp OR 'Qatar'/exp OR 'Republic of Korea'/exp OR 'Romania'/exp OR 'Saint Kitts and Nevis'/exp OR 'Saint Kitts Nevis' OR 'Saint Maarten*' OR 'Saint Martin*' OR 'San Marino'/exp OR 'San Marino*' OR 'Saudi Arabia'/exp OR 'Saudi Arabia*' OR 'Scotland'/exp OR 'Seychelles'/exp OR 'Singapore'/exp OR 'Slovakia'/exp OR 'Slovenia'/exp OR 'Spain'/exp OR 'Sweden'/exp OR 'Switzerland'/exp OR 'Taiwan'/exp OR 'Thailand'/exp OR 'Trinidad and Tobago'/exp OR 'Trinidad Tobago' OR 'Turks Caicos' OR 'U K' OR 'UK' OR 'United Arab Emirates'/exp OR 'United Arab Emirates' OR 'United Kingdom'/exp OR 'United Kingdom' OR 'United States Virgin Islands'/exp OR 'Uruguay'/exp OR 'Virgin Island*' OR Andorra* OR Aruba* OR Australia* OR Austria* OR Bahama* OR Bahrain* OR Barbados* OR Belgian* OR Belgium OR Bermuda* OR Britain OR British OR Brunei* OR Canada OR Canadian* OR Chile* OR Croatia* OR Curaçao* OR Cyprus* OR Dane* OR Danish OR Denmark OR Dutch* OR England OR English OR Estonia* OR Finland OR Finn* OR France OR French* OR German* OR Gibraltar* OR Greece* OR Greenland* OR Guam* OR Guernsey* OR Hungary* OR Iceland* OR Ireland OR Irish* OR Israel* OR Italian* OR Italy OR Japan* OR Korea* OR Kuwait* OR Latvia* OR Liechtenstein* OR Lithuania* OR Luxembourg* OR Macao* OR Malta* OR Monaco* OR Nauru* OR Netherlands OR New Caledonia* OR Norway OR Norwegian* OR Oman* OR Panama* OR Poland OR Pole* OR Polish OR Portugal OR Portuguese OR Puerto Rico* OR Qatar* OR Romania* OR Scot* OR Scotland OR Seychelle* OR Singapore* OR Slovakia* OR Slovenia* OR Spain OR Spaniard* OR Spanish OR Sweden OR Swedish OR Swiss OR Switzerland OR Taiwan* OR Thai* OR Uruguay* | 6,030,636 |
| **UK-Filter** | 26 | United Kingdom'/exp | 484,634 |
|  | 27 | ('national health service*' OR nhs OR 'national health service*':ad OR nhs:ad) | 526,717 |
|  | 28 | (english NOT ('published english' OR 'publication english' OR 'translate english' OR 'translated english' OR 'written english' OR 'language english' OR 'speak english' OR 'literature english' OR 'citation english')) | 43,464,662 |
|  | 29 | (gb OR 'g.b.' OR britain* OR (british* NOT 'british columbia') OR uk OR 'u.k.' OR united kingdom* OR (england* NOT 'new england') OR northern ireland* OR northern irish* OR scotland* OR scottish* OR ((wales OR 'south wales') NOT 'new south wales') OR welsh*) | 222,768 |
|  | 30 | (bath OR 'baths' OR ((birmingham NOT alabama*) OR ('birminghams' NOT alabama*) OR bradford OR 'bradfords' OR brighton OR 'brightons' OR bristol OR 'bristols' OR carlisle* OR 'carlisles' OR (cambridge NOT (massachusetts* OR boston* OR harvard*)) OR ('cambridges' NOT (massachusetts* OR boston* OR harvard*)) OR (canterbury NOT zealand*) OR ('canterburys' NOT zealand*) OR chelmsford OR 'chelmsfords' OR chester OR 'chesters' OR chichester OR 'chichesters' OR coventry OR 'coventrys' OR derby OR 'derbys' OR (durham NOT (carolina* OR nc)) OR ('durhams' NOT (carolina* OR nc)) OR ely OR 'elys' OR exeter OR 'exeters' OR gloucester OR 'gloucesters' OR hereford OR 'herefords' OR hull OR 'hulls' OR lancaster OR 'lancasters' OR leeds* OR leicester OR 'leicesters' OR (lincoln NOT nebraska*) OR ('lincolns' NOT nebraska*) OR (liverpool NOT (new south wales* OR nsw)) OR ('liverpools' NOT (new south wales* OR nsw)) OR ((london NOT (ontario* OR ont OR toronto*)) OR ('londons' NOT (ontario* OR ont OR toronto*)) OR manchester OR 'manchesters' OR (newcastle NOT (new south wales* OR nsw)) OR ('newcastles' NOT (new south wales* OR nsw)) OR norwich OR 'norwichs' OR nottingham OR 'nottinghams' OR oxford OR 'oxfords' OR peterborough OR 'peterboroughs' OR plymouth OR 'plymouths' OR portsmouth OR 'portsmouths' OR preston OR 'prestons' OR ripon OR 'ripons' OR salford OR 'salfords' OR salisbury OR 'salisburys' OR sheffield OR 'sheffields' OR southampton OR 'southamptons' OR st albans OR stoke OR 'stokes' OR sunderland OR 'sunderlands' OR truro OR 'truros' OR wakefield OR 'wakefields' OR wells OR westminster OR 'westminsters' OR winchester OR 'winchesters' OR wolverhampton OR 'wolverhamptons' OR (worcester NOT (massachusetts* OR boston* OR harvard*)) OR ('worcesters' NOT (massachusetts* OR boston* OR harvard*)) OR (york NOT ('new york*' OR ny OR ontario* OR ont OR toronto*)) OR ('yorks' NOT ('new york*' OR ny OR ontario* OR ont OR toronto*))))) | 1,860,950 |
|  | 31 | (bangor OR 'bangors' OR cardiff OR 'cardiffs' OR newport OR 'newports' OR 'st asaph' OR 'st asaphs' OR st davids OR swansea OR 'swanseas') | 22,381 |
|  | 32 | (aberdeen* OR dundee* OR edinburgh* OR glasgow* OR inverness OR (perth NOT australia*) OR stirling*) | 567,702 |
|  | 33 | (armagh OR 'armaghs' OR belfast OR 'belfasts' OR lisburn OR 'lisburns' OR londonderry OR 'londonderrys' OR derry OR 'derrys' OR newry OR 'newrys') | 61,970 |
|  | 34 | #26 OR #27 OR #28 OR #29 OR #30 OR #31 OR #32 OR #33 | 43,465,229 |
| **Canada-Filter** | 35 | Canada'/exp OR Canada OR Canad* | 1,972,009 |
|  | 36 | ('British Columbia' OR 'Colombie Britannique' OR Alberta* OR Saskatchewan OR Manitoba* OR Ontario OR Quebec OR 'Nouveau Brunswick' OR 'New Brunswick' OR 'Nova Scotia' OR 'Nouvelle Ecosse' OR 'Prince Edward Island' OR Newfoundland OR Labrador OR Nunavut OR NWT OR 'Northwest Territories' OR Yukon OR Nunavik OR Inuvialuit) | 724,265 |
|  | 37 | Abbotsford OR 'Ajax' OR 'Aurora' OR 'Barrie' OR 'Belleville' OR 'Blainville' OR 'Brampton' OR 'Brantford' OR 'Brossard' OR 'Burlington' OR 'Burnaby' OR 'Caledon' OR 'Calgary' OR 'cape breton' OR 'chatham kent' OR 'Chilliwack' OR 'Clarington' OR 'Coquitlam' OR 'Drummondville' OR 'Edmonton' OR 'Fredericton' OR 'fort mcmurray' OR 'Gatineau' OR 'Granby' OR 'grande prairie' OR 'Guelph' OR ('Halton Hills') OR 'Iqaluit' OR 'Inuvik' OR 'Kamloops' OR 'kawartha lakes' OR 'Kelowna' OR 'Kingston' OR 'Kitchener' OR 'Langley' OR 'Laval' OR 'Lethbridge' OR 'Levis' OR 'Longueuil' OR 'maple ridge' OR 'Markham' OR 'medicine hat' OR 'Milton' OR 'Mirabel' OR 'Mississauga' OR 'Moncton' OR 'Montreal' OR 'Nanaimo' OR 'new westminster' OR 'Newmarket' OR 'niagara falls' OR 'norfolk county' OR 'north bay' OR 'north vancouver' OR 'north vancouver' OR 'Oakville' OR 'Oshawa' OR 'Ottawa' OR 'port coquitlam' OR 'prince george' OR 'quebec city' OR 'red deer' OR 'Regina' OR 'Repentigny' OR 'Saanich' OR 'Saguenay' OR 'saint john' OR 'Saint-Hyacinthe' OR 'Saint-Jean-sur-Richelieu' OR 'Saint-Jerome' OR 'Sarnia' OR 'Saskatoon' OR 'sault ste marie' OR 'Sherbrooke' OR 'st albert' OR 'st catharines' OR 'st john s' OR ('Strathcona County') OR 'Terrebonne' OR 'thunder bay' OR 'Toronto' OR 'Trois-Rivieres' OR 'Vancouver' OR 'Vaughan' OR (('Airdrie' OR 'Cambridge' OR ('Halifax' OR 'Hamilton' OR 'London' OR 'Peterborough' OR 'Pickering' OR 'Richmond' OR 'richmond hill' OR 'Sudbury' OR 'Surrey' OR 'Victoria' OR 'Waterloo' OR 'Welland' OR 'Whitby' OR 'Windsor')) NOT ('UK' OR 'Britain' OR 'united kingdom' OR 'England' OR 'Australia')) OR 'Whitehorse' OR 'Winnipeg' OR 'wood buffalo' OR 'Yellowknife' | 2,502,895 |
|  | 38 | #35 OR #36 OR #37 | 2,988,916 |
| **Spain-Filter** | 39 | ((spain OR espagne OR espana OR spagna) OR (spain:ad OR espagne:ad OR espana:ad OR spanien:ad OR spagna:ad) OR (catalunya:ad OR catalonia:ad OR catalogne:ad OR cataluna:ad OR catala:ad OR barcelon*:ad OR tarragona:ad OR lleida:ad OR lerida:ad OR girona:ad OR gerona:ad OR sabadell:ad OR hospitalet:ad OR 'l hospitalet':ad) OR (valencia*:ad OR castello*:ad OR alacant:ad OR alicant*:ad) OR (murcia*:ad OR (cartagen*:ad NOT indias:ad)) OR (andalu*:ad OR sevill*:ad OR granad*:ad OR huelva:ad OR almeria:ad OR cadiz:ad OR jaen:ad OR malaga:ad OR (cordoba:ad NOT argentin*:ad)) OR (extremadura:ad OR caceres:ad OR badajoz:ad OR madrid:ad) OR (castilla:ad OR salamanca:ad OR zamora:ad OR valladolid:ad OR segovia:ad OR soria:ad OR palencia:ad OR avila:ad OR burgos:ad) OR (leon:ad NOT (france:ad OR clermont:ad OR rennes:ad OR lyon:ad OR USA:ad OR mexic*:ad)) OR (galicia:ad OR gallego:ad OR compostela:ad OR vigo:ad OR corun*:ad OR ferrol:ad OR orense:ad OR ourense:ad OR pontevedra:ad OR lugo:ad) OR (oviedo:ad OR gijon:ad OR asturia*:ad) OR (cantabr*:ad OR santander:ad) OR (vasco:ad OR euskadi:ad OR basque:ad OR bilbao:ad OR bilbo:ad OR donosti*:ad OR san sebastian:ad OR vizcaya:ad OR bizkaia:ad OR guipuzcoa:ad OR gipuzkoa:ad OR alava:ad OR araba:ad OR vitoria:ad OR gasteiz:ad) OR (navarr*:ad OR nafarroa:ad OR pamplona:ad OR iruna:ad OR irunea:ad) OR (logron*:ad OR rioj*:ad) OR (aragon*:ad OR zaragoza:ad OR teruel:ad OR huesca:ad) OR (mancha:ad OR ciudad real:ad OR albacete:ad OR cuenca:ad) OR (toledo:ad NOT (ohio:ad OR us:ad OR usa:ad OR OH:ad)) OR (guadalajara:ad NOT mexic*:ad) OR (balear*:ad OR mallorca:ad OR menorca:ad OR ibiza:ad OR eivissa:ad) OR (palmas:ad OR lanzarote:ad OR canari*:ad OR tenerif*:ad) OR (ceuta:ad OR melilla:ad)) OR (osasunbide*:ad OR osakidetza:ad OR insalud:ad OR sergas:ad OR catsalut:ad OR sespa:ad OR osasunbidea:ad OR imsalud:ad OR sescam:ad OR 'ib-salut':ad) | 1,655,396 |
| **Restrict to World Bank 'High-Income' Countries** | 40 | #24 AND (#25 OR #34 OR #38 OR #39) | 1,762 |
| **Restrict to Non-US / Non-African Countries** | 41 | #40 NOT (('africa'/exp OR 'north america'/exp OR 'united states'/exp) NOT ('asia'/exp OR 'australia'/exp OR 'canada'/exp OR 'europe'/exp OR 'south america'/exp)) | 1,132 |

# **Supplementary Table 1b. Search Terms for the HIV-1 Epidemiology Sources (PubMed)**

| **Term Group** | **#** | **Search Terms** | **Number of Hits  (July 2023)** |
| --- | --- | --- | --- |
| **Epidemiology: HIV-1**  **(with proximity 'free text' terms)** | 1 | "HIV Infections/epidemiology"[Majr] OR "HIV Seropositivity/epidemiology"[Majr] OR "HIV Seroprevalence"[Majr] | 44,264 |
|  | 2 | "HIV rate"[ti:~1] OR "HIV incidence"[ti:~1] OR "HIV prevalence"[ti:~1] OR "HIV use"[ti:~1] OR "HIV utilization"[ti:~1] OR "HIV infection"[ti:~1] OR "HIV epidemiology"[ti:~1] | 33,408 |
|  | 3 | #1 OR #2 | 70,039 |
| **Sex Workers** | 4 | "Sex Workers"[Mesh] OR "Sex Work"[Mesh] | 8,715 |
|  | 5 | "sex work"[tiab:~1] OR "sex work*"[tw] OR prostitute*[tw] OR prostitution[tw] OR "call girl*"[tw] OR "exchange sex"[tiab:~1] OR "ladies night"[tiab:~1] OR "sex payment"[tiab:~1] OR "survival sex"[tiab:~1] OR "transactional sex"[tiab:~1] OR "walk pavement"[tiab:~1] OR nightwalker*[tw] OR prostitut*[tw] OR sex industr*[tw] OR streetwalker*[tw] OR whore*[tw] | 16,443 |
|  | 6 | #4 OR #5 | 16,443 |
| **MSM** | 7 | "Homosexuality, Male"[Mesh] OR "Bisexuality"[Mesh] OR "Sexual and Gender Minorities"[Mesh] | 33,248 |
|  | 8 | "men who have sex with men"[tiab:~2] OR "men have sex with men"[tiab:~2] OR "men having sex with men"[tiab:~2] OR bisexual*[tw] OR homosexual*[tw] OR MSM[tw] OR "male to male sexual contact"[tw] OR "men sexual relations with men"[tiab:~1] OR "men who have sex with both men and women"[tiab:~1] OR "non binary"[tw] OR "non heterosexual*"[tw] OR bisexual*[tw] OR cruisin[tw] OR cruising*[tw] OR gay[tw] OR gays[tw] OR GBMSM[tw] OR GB-MSM[tw] OR homosexual*[tw] OR lesbigay*[tw] OR lgbt[tw] OR MASM[tw] OR MSM[tw] OR MSMW[tw] OR queer*[tw] | 58,462 |
|  | 9 | #7 OR #8 | 64,807 |
| **Transgender Persons** | 10 | "Transgender Persons"[Mesh] OR "Transsexualism"[Mesh] | 10,091 |
|  | 11 | "trans female*"[tw] OR "trans male*"[tw] OR "trans man"[tiab:~1] OR "trans men"[tiab:~1] OR "trans people"[tiab:~1] OR "trans people"[tiab:~1] OR "trans peoples"[tiab:~1] OR "trans person"[tiab:~1] OR "trans wom*"[tw] OR "transwom*"[tw] OR transman[tw] OR transmen[tw] OR transpeople[tw] OR transperson*[tw] OR "gender reassignment"[tiab:~1] OR "gender variant"[tiab:~1] OR "intersex individual*"[tw] OR "intersex people"[tiab:~1] OR "intersex person"[tiab:~1] OR "sexual dissident"[tiab:~1] OR "trans sexual*"[tw] OR "two spirit"[tiab:~1] OR glbt[tw] OR glbtq[tw] OR lgbt[tw] OR lgbtq[tw] OR lgbtqq[tw] OR transgender*[tw] | 16,117 |
|  | 12 | #10 OR #11 | 18,581 |
| **Drug Users** | 13 | "Drug Users"[Mesh] OR "Substance Abuse, Intravenous"[Mesh] | 19,401 |
|  | 14 | "I V drug user"[tiab:~3] OR "I V drug users"[tiab:~3] OR "injectable drug user"[tiab:~3] OR "injectable drug users"[tiab:~3] OR "intravenous drug user"[tiab:~3] OR "intra-venous drug user"[tiab:~3] OR "intravenous drug users"[tiab:~3] OR "intra-venous drug users"[tiab:~3] OR "IV drug user"[tiab:~3] OR "IV drug users"[tiab:~3] OR "IVDU"[tw] OR "IVDU's"[tiab:~1] OR "IVDUs"[tw] OR "people inject drugs"[tiab:~3] OR "people injected drugs"[tiab:~3] OR "people injecting drugs"[tiab:~3] OR "people who use drugs"[tiab:~3] OR "person inject drugs"[tiab:~3] OR "person who uses drugs"[tiab:~3] OR "persons injecting drugs"[tiab:~3] OR "persons who use drugs"[tiab:~3] OR "PWID"[tw] OR "PWIDs"[tw] OR "PWID's"[tw] OR "PWUD"[tw] OR "PWUDs"[tw] OR "PWUD's"[tw] OR "injecting drug user"[tiab:~1] OR "intravenous drug abuse"[tiab:~1] OR "intravenous drug use"[tiab:~1] OR "intravenous substance abuse"[tiab:~1] OR "intravenous substance misuse"[tiab:~1] OR "intravenous substance use"[tiab:~1] OR "parenteral drug abuse"[tiab:~1] OR "parenteral drug use"[tiab:~1] OR "people who inject drugs"[tiab:~1] OR "person who inject drugs"[tiab:~1] OR "persons who inject drugs"[tiab:~1] OR "injecting drug users"[tiab:~1] OR "intravenous drug abuser"[tiab:~1] OR "intravenous drug user"[tiab:~1] OR "intravenous substance abuser"[tiab:~1] OR "intravenous substance misuser"[tiab:~1] OR "intravenous substance user"[tiab:~1] OR "parenteral drug abuser"[tiab:~1] OR "parenteral drug user"[tiab:~1] OR "intravenous drug abusers"[tiab:~1] OR "intravenous drug users"[tiab:~1] OR "intravenous substance abusers"[tiab:~1] OR "intravenous substance misusers"[tiab:~1] OR "intravenous substance users"[tiab:~1] OR "parenteral drug abusers"[tiab:~1] OR "parenteral drug users"[tiab:~1] OR (((inject*[ti] OR intraven*[ti] OR parenteral[ti] OR use[ti] OR misus*[ti] OR user[ti] OR users[ti]) AND (drug*[ti] OR substance*[ti])) OR ((inject*[ot] OR intraven*[ot] OR parenteral[ot]) AND (drug*[ot] OR substance*[ot]))) | 72,690 |
|  | 15 | #13 OR #14 | 79,777 |
| **Prisoner Population** | 16 | "Correctional Facilities"[Mesh] OR "Prisons"[Mesh] OR "Jails"[Mesh] OR "Prisoners"[Mesh] | 26,011 |
|  | 17 | "correctional facilities"[tiab:~1] OR "correctional facility"[tiab:~1] OR "correctional institution"[tw] OR "correctional institutions"[tw] OR "gaol"[tw] OR "gaols"[tw] OR "penal facilities"[tiab:~1] OR "penal facility"[tiab:~1] OR "penal institution"[tiab:~1] OR "penal institutions"[tiab:~1] OR custodial[tw] OR imprison*[tw] OR incarcerat*[tw] OR inmate*[tw] OR jail[tw] OR jails[tw] OR penitentiar*[tw] OR prison*[tw] OR "closed setting*"[tw] OR cellmate*[tw] OR confinement*[tw] OR detainee*[tw] OR detention*[tw] OR offender[tw] OR offenders[tw] OR penal[tw] OR penitentiar*[tw] OR reformator*[tw] OR remand*[tw] OR convict*[tw] | 91,727 |
|  | 18 | #16 OR #17 | 92,156 |
| **Serodiscordant Couples** | 19 | serodiscordan*[tw] OR "sero-discordan*"[tw] OR "mixed infection status"[tiab:~2] | 1,383 |
| **All Target Patient Populations** | 20 | #6 OR #9 OR #12 OR #15 OR #18 OR #19 | 246,656 |
| **Epidemiology in Target Populations** | 21 | #3 AND #20 | 16,135 |
| **Restrict to Publication On/After  1st Jan 2019** | 22 | #21 AND (2019/1/1:3000/12/12[pdat]) | 3,763 |
| **Remove Irrelevant Publication Types - Expanded** | 23 | #22 NOT ("Review"[Publication Type] OR "Systematic Review"[Publication Type] OR "Meta-Analysis"[Publication Type] OR "Case Reports" [Publication Type] OR "Published Erratum"[Publication Type] OR "comment"[Publication Type] OR "Editorial"[Publication Type] OR "Controlled Clinical Trial"[Publication Type] OR "Randomized Controlled Trial"[Publication Type] OR "Clinical Trial, Phase III" [Publication Type]) | 3,284 |
| **Animal Studies** | 24 | #23 NOT ("animal cell"[tiab] OR "animal experiment"[tiab] OR "animal health"[tiab] OR "animal model"[tiab] OR "animal tissue"[tiab] OR "bird"[tiab] OR "cancer model"[tiab] OR "canine"[tiab] OR "dog"[tiab] OR "ex vivo study"[tiab] OR "goat"[tiab] OR "Hep-G2 cell line"[tiab] OR "in vitro study"[tiab] OR "knockout mouse"[tiab] OR "Kupffer cell"[tiab] OR "mammal"[tiab] OR "model"[tiab] OR "mouse"[tiab] OR "mouse model"[tiab] OR "murine"[tiab] OR mice[tiab] OR "nonhuman"[tiab] OR "nude rat"[tiab] OR "rat"[tiab] OR "rat model"[tiab] OR "tumor cell"[tiab] OR "tumor model"[tiab] OR "tumor xenograft"[tiab] OR "veterinary"[tiab]) | 2,807 |
| **World Bank 'High-Income' Countries** | 25 | "Andorra"[Mesh] OR "Antigua and Barbuda"[Mesh] OR "Antigua Barbuda"[tiab:~1] OR "Aruba"[Mesh] OR "Asia"[Mesh] OR "Australia"[Mesh] OR "Austria"[Mesh] OR "Bahamas"[Mesh] OR "Bahrain"[Mesh] OR "Barbados"[Mesh] OR "Belgium"[Mesh] OR "Bermuda"[Mesh] OR "British Virgin Islands"[Mesh] OR "Brunei"[Mesh] OR "Canada"[Mesh] OR "Cayman Island*"[tw] OR "Channel Islands"[Mesh] OR "Chile"[Mesh] OR "Cook Island*"[tw] OR "Croatia"[Mesh] OR "Curacao"[Mesh] OR "Cyprus"[Mesh] OR "Czech Republic"[Mesh] OR "Czech Republic*"[tw] OR "Denmark"[Mesh] OR "England"[Mesh] OR "Estonia"[Mesh] OR "Europe"[Mesh] OR "Faroe Island*"[tw] OR "Finland"[Mesh] OR "France"[Mesh] OR "French Polynesia*"[tw] OR "Germany"[Mesh] OR "Gibraltar"[Mesh] OR "Great Britain"[tiab:~1] OR "Great Britain"[tiab:~1] OR "Greece"[Mesh] OR "Greenland"[Mesh] OR "Guam"[Mesh] OR "Guernsey"[Mesh] OR "Hong Kong*"[tw] OR "Hungary"[Mesh] OR "Iceland"[Mesh] OR "Ireland"[Mesh] OR "Isle of Man"[tw] OR "Israel"[Mesh] OR "Italy"[Mesh] OR "Japan"[Mesh] OR "Jersey Channel Island*"[tw] OR "Kuwait"[Mesh] OR "Latvia"[Mesh] OR "Liechtenstein"[Mesh] OR "Lithuania"[Mesh] OR "Luxembourg"[Mesh] OR "Macau"[Mesh] OR "Malta"[Mesh] OR "Monaco"[Mesh] OR "Netherlands"[Mesh] OR "New Caledonia"[Mesh] OR "New Zealand"[Mesh] OR "New Zealand*"[tw] OR "Northern Mariana Island*"[tw] OR "Norway"[Mesh] OR "Oman"[Mesh] OR "Panama"[Mesh] OR "Poland"[Mesh] OR "Polynesia"[Mesh] OR "Portugal"[Mesh] OR "Puerto Rico"[Mesh] OR "Qatar"[Mesh] OR "Republic of Korea"[Mesh] OR "Romania"[Mesh] OR "Saint Kitts and Nevis"[Mesh] OR "Saint Kitts Nevis"[tiab:~1] OR "Saint Maarten*"[tw] OR "Saint Martin*"[tw] OR "San Marino"[Mesh] OR "San Marino*"[tw] OR "Saudi Arabia"[Mesh] OR "Saudi Arabia*"[tw] OR "Scotland"[Mesh] OR "Seychelles"[Mesh] OR "Singapore"[Mesh] OR "Slovakia"[Mesh] OR "Slovenia"[Mesh] OR "Spain"[Mesh] OR "Sweden"[Mesh] OR "Switzerland"[Mesh] OR "Taiwan"[Mesh] OR "Thailand"[Mesh] OR "Trinidad and Tobago"[Mesh] OR "Trinidad Tobago"[tiab:~1] OR "Turks Caicos"[tiab:~1] OR "U K"[tw] OR "UK"[tw] OR "United Arab Emirates"[Mesh] OR "United Arab Emirates"[tw] OR "United Kingdom"[Mesh] OR "United Kingdom"[tw] OR "United States Virgin Islands"[Mesh] OR "Uruguay"[Mesh] OR "Virgin Island*"[tw] OR Andorra*[tw] OR Aruba*[tw] OR Australia*[tw] OR Austria*[tw] OR Bahama*[tw] OR Bahrain*[tw] OR Barbados*[tw] OR Belgian*[tw] OR Belgium[tw] OR Bermuda*[tw] OR Britain[tw] OR British[tw] OR Brunei*[tw] OR Canada[tw] OR Canadian*[tw] OR Chile*[tw] OR Croatia*[tw] OR Curaçao*[tw] OR Cyprus*[tw] OR Dane*[tw] OR Danish[tw] OR Denmark[tw] OR Dutch*[tw] OR England[tw] OR English[tw] OR Estonia*[tw] OR Finland[tw] OR Finn*[tw] OR France[tw] OR French*[tw] OR German*[tw] OR Gibraltar*[tw] OR Greece*[tw] OR Greenland*[tw] OR Guam*[tw] OR Guernsey*[tw] OR Hungary*[tw] OR Iceland*[tw] OR Ireland[tw] OR Irish*[tw] OR Israel*[tw] OR Italian*[tw] OR Italy[tw] OR Japan*[tw] OR Korea*[tw] OR Kuwait*[tw] OR Latvia*[tw] OR Liechtenstein*[tw] OR Lithuania*[tw] OR Luxembourg*[tw] OR Macao*[tw] OR Malta*[tw] OR Monaco*[tw] OR Nauru*[tw] OR Netherlands[tw] OR New Caledonia*[tw] OR Norway[tw] OR Norwegian*[tw] OR Oman*[tw] OR Panama*[tw] OR Poland[tw] OR Pole*[tw] OR Polish[tw] OR Portugal[tw] OR Portuguese[tw] OR Puerto Rico*[tw] OR Qatar*[tw] OR Romania*[tw] OR Scot*[tw] OR Scotland[tw] OR Seychelle*[tw] OR Singapore*[tw] OR Slovakia*[tw] OR Slovenia*[tw] OR Spain[tw] OR Spaniard*[tw] OR Spanish[tw] OR Sweden[tw] OR Swedish[tw] OR Swiss[tw] OR Switzerland[tw] OR Taiwan*[tw] OR Thai*[tw] OR Uruguay*[tw] | 5,394,188 |
| **UK-Filter** | 26 | "United Kingdom"[Mesh] | 390,209 |
|  | 27 | ("national health service*"[tiab] OR nhs[tiab] OR "national health service*"[ad] OR nhs[ad]) | 270,709 |
|  | 28 | (english[tiab] NOT ("published english"[tiab:~5] OR "publication english"[tiab:~5] OR "translate english"[tiab:~5] OR "translated english"[tiab:~5] OR "written english"[tiab:~5] OR "language english"[tiab:~5] OR "speak english"[tiab:~5] OR "literature english"[tiab:~5] OR "citation english"[tiab:~5])) | 64,080 |
|  | 29 | (gb[tiab] OR "g.b."[tiab] OR britain*[tiab] OR (british*[tiab] NOT "british columbia"[tiab]) OR uk[tiab] OR "u.k."[tiab] OR united kingdom*[tiab] OR (england*[tiab] NOT "new england"[tiab:~0]) OR northern ireland*[tiab] OR northern irish*[tiab] OR scotland*[tiab] OR scottish*[tiab] OR ((wales[tiab] OR "south wales"[tiab:~0]) NOT "new south wales"[tiab:~0]) OR welsh*[tiab]) | 326,654 |
|  | 30 | (bath[tiab] OR "bath's"[tiab] OR ((birmingham[tiab] NOT alabama*[tiab]) OR ("birmingham's"[tiab] NOT alabama*[tiab]) OR bradford[tiab] OR "bradford's"[tiab] OR brighton[tiab] OR "brighton's"[tiab] OR bristol[tiab] OR "bristol's"[tiab] OR carlisle*[tiab] OR "carlisle's"[tiab] OR (cambridge[tiab] NOT (massachusetts*[tiab] OR boston*[tiab] OR harvard*[tiab])) OR ("cambridge's"[tiab] NOT (massachusetts*[tiab] OR boston*[tiab] OR harvard*[tiab])) OR (canterbury[tiab] NOT zealand*[tiab]) OR ("canterbury's"[tiab] NOT zealand*[tiab]) OR chelmsford[tiab] OR "chelmsford's"[tiab] OR chester[tiab] OR "chester's"[tiab] OR chichester[tiab] OR "chichester's"[tiab] OR coventry[tiab] OR "coventry's"[tiab] OR derby[tiab] OR "derby's"[tiab] OR (durham[tiab] NOT (carolina*[tiab] OR nc[tiab])) OR ("durham's"[tiab] NOT (carolina*[tiab] OR nc[tiab])) OR ely[tiab] OR "ely's"[tiab] OR exeter[tiab] OR "exeter's"[tiab] OR gloucester[tiab] OR "gloucester's"[tiab] OR hereford[tiab] OR "hereford's"[tiab] OR hull[tiab] OR "hull's"[tiab] OR lancaster[tiab] OR "lancaster's"[tiab] OR leeds*[tiab] OR leicester[tiab] OR "leicester's"[tiab] OR (lincoln[tiab] NOT nebraska*[tiab]) OR ("lincoln's"[tiab] NOT nebraska*[tiab]) OR (liverpool[tiab] NOT (new south wales*[tiab] OR nsw[tiab])) OR ("liverpool's"[tiab] NOT (new south wales*[tiab] OR nsw[tiab])) OR ((london[tiab] NOT (ontario*[tiab] OR ont[tiab] OR toronto*[tiab])) OR ("london's"[tiab] NOT (ontario*[tiab] OR ont[tiab] OR toronto*[tiab])) OR manchester[tiab] OR "manchester's"[tiab] OR (newcastle[tiab] NOT (new south wales*[tiab] OR nsw[tiab])) OR ("newcastle's"[tiab] NOT (new south wales*[tiab] OR nsw[tiab])) OR norwich[tiab] OR "norwich's"[tiab] OR nottingham[tiab] OR "nottingham's"[tiab] OR oxford[tiab] OR "oxford's"[tiab] OR peterborough[tiab] OR "peterborough's"[tiab] OR plymouth[tiab] OR "plymouth's"[tiab] OR portsmouth[tiab] OR "portsmouth's"[tiab] OR preston[tiab] OR "preston's"[tiab] OR ripon[tiab] OR "ripon's"[tiab] OR salford[tiab] OR "salford's"[tiab] OR salisbury[tiab] OR "salisbury's"[tiab] OR sheffield[tiab] OR "sheffield's"[tiab] OR southampton[tiab] OR "southampton's"[tiab] OR st albans[tiab] OR stoke[tiab] OR "stoke's"[tiab] OR sunderland[tiab] OR "sunderland's"[tiab] OR truro[tiab] OR "truro's"[tiab] OR wakefield[tiab] OR "wakefield's"[tiab] OR wells[tiab] OR westminster[tiab] OR "westminster's"[tiab] OR winchester[tiab] OR "winchester's"[tiab] OR wolverhampton[tiab] OR "wolverhampton's"[tiab] OR (worcester[tiab] NOT (massachusetts*[tiab] OR boston*[tiab] OR harvard*[tiab])) OR ("worcester's"[tiab] NOT (massachusetts*[tiab] OR boston*[tiab] OR harvard*[tiab])) OR (york[tiab] NOT ("new york*"[tiab] OR ny[tiab] OR ontario*[tiab] OR ont[tiab] OR toronto*[tiab])) OR ("york's"[tiab] NOT ("new york*"[tiab] OR ny[tiab] OR ontario*[tiab] OR ont[tiab] OR toronto*[tiab]))))) | 195,029 |
|  | 31 | (bangor[tiab] OR "bangor's"[tiab] OR cardiff[tiab] OR "cardiff's"[tiab] OR newport[tiab] OR "newport's"[tiab] OR "st asaph"[tiab] OR "st asaph's"[tiab] OR st davids[tiab] OR swansea[tiab] OR "swansea's"[tiab]) | 3,417 |
|  | 32 | (aberdeen[tiab] OR "aberdeen's"[tiab] OR dundee[tiab] OR "dundee's"[tiab] OR edinburgh[tiab] OR "edinburgh's"[tiab] OR glasgow[tiab] OR "glasgow's"[tiab] OR inverness[tiab] OR (perth[tiab] NOT australia*[tiab]) OR ("perth's"[tiab] NOT australia*[tiab]) OR stirling[tiab] OR "stirling's"[tiab]) | 41,936 |
|  | 33 | (armagh[tiab] OR "armagh's"[tiab] OR belfast[tiab] OR "belfast's"[tiab] OR lisburn[tiab] OR "lisburn's"[tiab] OR londonderry[tiab] OR "londonderry's"[tiab] OR derry[tiab] OR "derry's"[tiab] OR newry[tiab] OR "newry's"[tiab]) | 1,559 |
|  | 34 | #26 OR #27 OR #28 OR #29 OR #30 OR #31 OR #32 OR #33 | 991,125 |
|  | 35 | Canada[Mesh] OR Canada[tw] OR Canad*[tw] | 253,141 |
|  | 36 | ("British Columbia"[tiab] OR "Colombie Britannique"[tiab] OR Alberta*[tiab] OR Saskatchewan[tiab] OR Manitoba*[tiab] OR Ontario[tiab] OR Quebec[tiab] OR "Nouveau Brunswick"[tiab] OR "New Brunswick"[tiab] OR "Nova Scotia"[tiab] OR "Nouvelle Ecosse"[tiab] OR "Prince Edward Island"[tiab] OR Newfoundland[tiab] OR Labrador[tiab] OR Nunavut[tiab] OR NWT[tiab] OR "Northwest Territories"[tiab] OR Yukon[tiab] OR Nunavik[tiab] OR Inuvialuit[tiab]) | 86,049 |
|  | 37 | Abbotsford[tiab] OR "Ajax"[tiab] OR "Aurora"[tiab] OR "Barrie"[tiab] OR "Belleville"[tiab] OR "Blainville"[tiab] OR "Brampton"[tiab] OR "Brantford"[tiab] OR "Brossard"[tiab] OR "Burlington"[tiab] OR "Burnaby"[tiab] OR "Caledon"[tiab] OR "Calgary"[tiab] OR "cape breton"[tiab] OR "chatham kent"[tiab] OR "Chilliwack"[tiab] OR "Clarington"[tiab] OR "Coquitlam"[tiab] OR "Drummondville"[tiab] OR "Edmonton"[tiab] OR "Fredericton"[tiab] OR "fort mcmurray"[tiab] OR "Gatineau"[tiab] OR "Granby"[tiab] OR "grande prairie"[tiab] OR "Guelph"[tiab] OR ("Halton Hills"[tiab]) OR "Iqaluit"[tiab] OR "Inuvik"[tiab] OR "Kamloops"[tiab] OR "kawartha lakes"[tiab] OR "Kelowna"[tiab] OR "Kingston"[tiab] OR "Kitchener"[tiab] OR "Langley"[tiab] OR "Laval"[tiab] OR "Lethbridge"[tiab] OR "Levis"[tiab] OR "Longueuil"[tiab] OR "maple ridge"[tiab] OR "Markham"[tiab] OR "medicine hat"[tiab] OR "Milton"[tiab] OR "Mirabel"[tiab] OR "Mississauga"[tiab] OR "Moncton"[tiab] OR "Montreal"[tiab] OR "Nanaimo"[tiab] OR "new westminster"[tiab] OR "Newmarket"[tiab] OR "niagara falls"[tiab] OR "norfolk county"[tiab] OR "north bay"[tiab] OR "north vancouver"[tiab] OR "north vancouver"[tiab] OR "Oakville"[tiab] OR "Oshawa"[tiab] OR "Ottawa"[tiab] OR "port coquitlam"[tiab] OR "prince george"[tiab] OR "quebec city"[tiab] OR "red deer"[tiab] OR "Regina"[tiab] OR "Repentigny"[tiab] OR "Saanich"[tiab] OR "Saguenay"[tiab] OR "saint john"[tiab] OR "Saint-Hyacinthe"[tiab] OR "Saint-Jean-sur-Richelieu"[tiab] OR "Saint-Jerome"[tiab] OR "Sarnia"[tiab] OR "Saskatoon"[tiab] OR "sault ste marie"[tiab] OR "Sherbrooke"[tiab] OR "st albert"[tiab] OR "st catharines"[tiab] OR "st john s"[tiab] OR ("Strathcona County"[tiab:~2]) OR "Terrebonne"[tiab] OR "thunder bay"[tiab] OR "Toronto"[tiab] OR "Trois-Rivieres"[tiab] OR "Vancouver"[tiab] OR "Vaughan"[tiab] OR (("Airdrie"[tiab] OR "Cambridge"[tiab] OR ("Halifax"[tiab] OR "Hamilton"[tiab] OR "London"[tiab] OR "Peterborough"[tiab] OR "Pickering"[tiab] OR "Richmond"[tiab] OR "richmond hill"[tiab] OR "Sudbury"[tiab] OR "Surrey"[tiab] OR "Victoria"[tiab] OR "Waterloo"[tiab] OR "Welland"[tiab] OR "Whitby"[tiab] OR "Windsor"[tiab])) NOT ("UK"[tw] OR "Britain"[tw] OR "united kingdom"[tw] OR "England"[tw] OR "Australia"[tw])) OR "Whitehorse"[tiab] OR "Winnipeg"[tiab] OR "wood buffalo"[tiab] OR "Yellowknife"[tiab] | 136,555 |
|  | 38 | #35 OR #36 OR #37 | 382,287 |
| **Spain-Filter** | 39 | ((spain OR espagne OR espana OR spagna) OR (spain[ad] OR espagne[ad] OR espana[ad] OR spanien[ad] OR spagna[ad]) OR (catalunya[ad] OR catalonia[ad] OR catalogne[ad] OR cataluna[ad] OR catala[ad] OR barcelon*[ad] OR tarragona[ad] OR lleida[ad] OR lerida[ad] OR girona[ad] OR gerona[ad] OR sabadell[ad] OR hospitalet[ad] OR l’hospitalet[ad]) OR (valencia*[ad] OR castello*[ad] OR alacant[ad] OR alicant*[ad]) OR (murcia*[ad] OR (cartagen*[ad] NOT indias[ad])) OR (andalu*[ad] OR sevill*[ad] OR granad*[ad] OR huelva[ad] OR almeria[ad] OR cadiz[ad] OR jaen[ad] OR malaga[ad] OR (cordoba[ad] NOT argentin*[ad])) OR (extremadura[ad] OR caceres[ad] OR badajoz[ad] OR madrid[ad]) OR (castilla[ad] OR salamanca[ad] OR zamora[ad] OR valladolid[ad] OR segovia[ad] OR soria[ad] OR palencia[ad] OR avila[ad] OR burgos[ad]) OR (leon[ad] NOT (france[ad] OR clermont[ad] OR rennes[ad] OR lyon[ad] OR USA[ad] OR mexic*[ad])) OR (galicia[ad] OR gallego[ad] OR compostela[ad] OR vigo[ad] OR corun*[ad] OR ferrol[ad] OR orense[ad] OR ourense[ad] OR pontevedra[ad] OR lugo[ad]) OR (oviedo[ad] OR gijon[ad] OR asturia*[ad]) OR (cantabr*[ad] OR santander[ad]) OR (vasco[ad] OR euskadi[ad] OR basque[ad] OR bilbao[ad] OR bilbo[ad] OR donosti*[ad] OR san sebastian[ad] OR vizcaya[ad] OR bizkaia[ad] OR guipuzcoa[ad] OR gipuzkoa[ad] OR alava[ad] OR araba[ad] OR vitoria[ad] OR gasteiz[AD]) OR (navarr*[ad] OR nafarroa[ad] OR pamplona[ad] OR iruna[ad] OR irunea[ad]) OR (logron*[ad] OR rioj*[ad]) OR (aragon*[ad] OR zaragoza[ad] OR teruel[ad] OR huesca[ad]) OR (mancha[ad] OR ciudad real[ad] OR albacete[ad] OR cuenca[ad]) OR (toledo[ad] NOT (ohio[ad] OR us[ad] OR usa[ad] OR OH[ad])) OR (guadalajara[ad] NOT mexic*[ad]) OR (balear*[ad] OR mallorca[ad] OR menorca[ad] OR ibiza[ad] OR eivissa[ad]) OR (palmas[ad] OR lanzarote[ad] OR canari*[ad] OR tenerif*[ad]) OR (ceuta[ad] OR melilla[ad])) OR (osasunbide*[ad] OR osakidetza[ad] OR insalud[ad] OR sergas[ad] OR catsalut[ad] OR sespa[ad] OR osasunbidea[ad] OR imsalud[ad] OR sescam[ad] OR ib-salut[ad]) | 803,004 |
| **Restrict to World Bank 'High-Income' Countries** | 40 | #24 AND (#25 OR #34 OR #38 OR #39) | 1,255 |
| **Restrict to Non-US / Non-African countries** | 41 | #40 NOT ((africa[mesh] OR north america[mesh:noexp] OR united states[mesh]) NOT (asia[mesh] OR australia[mesh] OR canada[mesh] OR europe[mesh] OR south america[mesh])) | 1,208 |

# **Supplementary Table 1c. Search Terms for the HIV-1 Epidemiology Sources (Cochrane Library)**

| **#** | **Search Terms** | **Number of Hits  (July 2023)** |
| --- | --- | --- |
| 1 | MeSH descriptor: [HIV Infections] explode all trees and with qualifier(s): [epidemiology - EP] | 1685 |
| 2 | MeSH descriptor: [HIV Seropositivity] explode all trees and with qualifier(s): [epidemiology - EP] | 111 |
| 3 | MeSH descriptor: [HIV Seroprevalence] explode all trees | 23 |
| 4 | HIV NEAR/1 rate:ti OR HIV NEAR/1 incidence:ti OR HIV NEAR/1 prevalence:ti OR HIV NEAR/1 use:ti OR HIV NEAR/1 utilization:ti OR HIV NEAR/1 infection:ti OR HIV NEAR/1 epidemiology:ti | 1224 |
| 5 | #1 OR #2 OR #3 OR #4 | 2810 |
| 6 | MeSH descriptor: [Sex Workers] explode all trees | 119 |
| 7 | MeSH descriptor: [Sex Work] explode all trees | 132 |
| 8 | Sex NEAR/1 work OR sex NEXT work OR sex NEXT work* OR prostitute* OR prostitution OR call NEXT girl* OR exchange NEAR/1 sex OR ladies NEAR/1 night OR sex NEAR/1 payment OR survival NEAR/1 sex OR transactional NEAR/1 sex OR walk NEAR/1 pavement OR nightwalker OR prostitut* OR sex NEXT industr* OR streetwalker* OR whore* | 909 |
| 9 | #6 OR #7 OR #8 | 909 |
| 10 | MeSH descriptor: [Homosexuality, Male] explode all trees | 668 |
| 11 | MeSH descriptor: [Bisexuality] explode all trees | 86 |
| 12 | MeSH descriptor: [Sexual and Gender Minorities] explode all trees | 421 |
| 13 | Bisex* OR bisexuality OR bisexual OR Bi-sex* OR bi-sexuality OR bi-sexual OR Men NEAR/2 who have sex with men OR men NEAR/2 have sex with men OR men NEAR/2 having sex with men OR bisexual* OR homosexual* OR MSM OR 'male to male sexual contact' OR men NEAR/1 sexual relations with men OR men NEAR/1 who have sex with both men and women OR 'non binary' OR non NEXT heterosexual* OR bisexual* OR cruisin OR cruising* OR gay OR gays OR GBMSM OR GB-MSM OR homosexual* OR lesbigay* OR lgbt OR MASM OR MSM OR MSMW OR queer* | 101729 |
| 14 | #10 OR #11 OR #12 OR #13 | 101784 |
| 15 | MeSH descriptor: [Transgender Persons] explode all trees | 109 |
| 16 | MeSH descriptor: [Transsexualism] explode all trees | 40 |
| 17 | trans NEXT female* OR trans NEXT male* OR trans NEAR/1 men OR trans NEAR/1 people OR trans NEAR/1 peoples OR trans NEAR/1 person OR trans NEXT wom* OR transwom* OR transman OR transmen OR transpeople OR transperson* OR gender NEAR/1 reassignment OR gender NEAR/1 variant OR intersex NEXT individual* OR intersex NEAR/1 people OR intersex NEAR/1 person OR sexual NEAR/1 dissident OR trans NEXT sexual* OR two NEAR/1 spirit OR glbt OR glbtq OR lgbt OR lgbtq OR lgbtqq OR transgender* OR trans NEAR/1 man | 759 |
| 18 | #15 OR #16 OR #17 | 777 |
| 19 | MeSH descriptor: [Drug Users] explode all trees | 149 |
| 20 | MeSH descriptor: [Substance Abuse, Intravenous] explode all trees | 491 |
| 21 | I V NEAR/3 drug user OR I V NEAR/3 drug users OR injectable NEAR/3 drug user OR injectable NEAR/3 drug users OR intravenous NEAR/3 drug user OR intra-venous NEAR/3 drug user OR intravenous NEAR/3 drug users OR intra-venous NEAR/3 drug users OR IV NEAR/3 drug user OR IV NEAR/3 drug users OR IVDU OR IVDUs OR IVDUs OR people NEAR/3 inject drugs OR people NEAR/3 injected drugs OR people NEAR/3 injecting drugs OR people NEAR/3 who use drugs OR person NEAR/3 inject drugs OR person NEAR/3 who use drugs OR persons NEAR/3 injecting drugs OR persons NEAR/3 who use drugs OR PWID OR PWIDs OR PWIDs OR PWUD OR PWUDs OR PWUDs OR injecting NEAR/1 drug user OR intravenous NEAR/1 drug abuse OR intravenous NEAR/1 drug use OR intravenous NEAR/1 substance abuse OR intravenous NEAR/1 substance misuse OR intravenous NEAR/1 substance use OR parenteral NEAR/1 drug abuse OR parenteral NEAR/1 drug use OR people NEAR/1 who inject drugs OR person NEAR/1 who inject drugs OR persons NEAR/1 who inject drugs OR injecting NEAR/1 drug users OR intravenous NEAR/1 drug abuser OR intravenous NEAR/1 drug user OR intravenous NEAR/1 substance abuser OR intravenous NEAR/1 substance misuser OR intravenous NEAR/1 substance user OR parenteral NEAR/1 drug abuser OR parenteral NEAR/1 drug user OR intravenous NEAR/1 drug abusers OR intravenous NEAR/1 drug users OR intravenous NEAR/1 substance abusers OR intravenous NEAR/1 substance misusers OR intravenous NEAR/1 substance users OR parenteral NEAR/1 drug abusers OR parenteral NEAR/1 drug users OR ((inject*:ti OR intraven*:ti OR parenteral:ti OR use:ti OR misus*:ti OR user:ti OR users:ti) AND (drug*:ti OR substance*:ti)) | 49939 |
| 22 | #19 OR #20 OR #21 | 49984 |
| 23 | MeSH descriptor: [Correctional Facilities] explode all trees | 189 |
| 24 | MeSH descriptor: [Jails] explode all trees | 7 |
| 25 | MeSH descriptor: [Prisons] explode all trees | 182 |
| 26 | MeSH descriptor: [Prisoners] explode all trees | 421 |
| 27 | correctional NEAR/1 facilities OR correctional NEAR/1 facility OR 'correctional institution' OR 'correctional institutions' OR gaol OR gaols OR penal NEAR/1 facilities OR penal NEAR/1 facility OR penal NEAR/1 institution OR penal NEAR/1 institutions OR custodial OR imprison* OR incarcerat* OR inmate* OR jail OR jails OR penitentiar* OR prison* OR closed NEXT setting* OR cellmate* OR confinement* OR detainee* OR detention* OR offender OR offenders OR penal OR penitentiar* OR reformator* OR remand* OR convict* | 4010 |
| 28 | #23 OR #24 OR #25 OR #26 OR #27 | 4012 |
| 29 | serodiscordan* OR sero-discordan* OR sero NEXT discordan* OR mixed NEAR/2 infection status | 900 |
| 30 | #9 OR #14 OR #18 OR #22 OR #28 OR #29 | 149532 |
| 31 | #5 AND #30 | 906 |
| 32 | #31 with Publication Year from 2019 to 2023, in Trials | 283 |
| 33 | MeSH descriptor: [Randomized Controlled Trial] explode all trees | 25732 |
| 34 | MeSH descriptor: [Randomized Controlled Trials as Topic] explode all trees | 47396 |
| 35 | MeSH descriptor: [Controlled Clinical Trial] explode all trees | 38477 |
| 36 | MeSH descriptor: [Controlled Clinical Trials as Topic] explode all trees | 47802 |
| 37 | MeSH descriptor: [Review] explode all trees | 1002 |
| 38 | MeSH descriptor: [Review Literature as Topic] explode all trees | 435 |
| 39 | #33 OR #34 OR #35 OR #36 OR #37 OR #38 | 86578 |
| 40 | #32 NOT #39 | 265 |
| 41 | ('animal cell' OR 'animal experiment' OR 'animal health' OR 'animal model' OR 'animal tissue' OR 'bird' OR 'cancer model' OR 'canine' OR 'dog' OR 'ex vivo study' OR 'goat' OR 'Hep-G2 cell line' OR 'in vitro study' OR 'knockout mouse' OR 'Kupffer cell' OR 'mammal' OR 'model' OR 'mouse' OR 'mouse model' OR 'murine' OR mice OR 'nonhuman' OR 'nude rat' OR 'rat' OR 'rat model' OR 'tumor cell' OR 'tumor model' OR 'tumor xenograft' OR 'veterinary') | 200247 |
| 42 | #40 NOT #41 | 166 |
| 43 | MeSH descriptor: [Africa] explode all trees | 11427 |
| 44 | MeSH descriptor: [North America] this term only | 715 |
| 45 | MeSH descriptor: [United States] explode all trees | 27438 |
| 46 | MeSH descriptor: [Asia] explode all trees | 33380 |
| 47 | MeSH descriptor: [Australia] explode all trees | 6637 |
| 48 | MeSH descriptor: [Canada] explode all trees | 5612 |
| 49 | MeSH descriptor: [Europe] explode all trees | 41960 |
| 50 | MeSH descriptor: [South America] explode all trees | 3876 |
| 51 | (#43 OR #44 OR #45) NOT (#46 OR #47 Or #48 OR 49 OR #50) | 34386 |
| 52 | #42 NOT #51 | **95** |

# **Supplementary Table 2a. Search Terms for Prevalence of PrEP Use Sources (Embase)**

| **Term Group** | **#** | **Search Terms** | **Number of Hits  (July 2023)** |
| --- | --- | --- | --- |
| **Pre-Exposure Prophylaxis (PrEP)** | 1 | "Pre-Exposure Prophylaxis"[Mesh] OR "Chemoprevention"[Mesh] | 53,370 |
|  | 2 | "pre‐exposure prophylaxis"[tiab:~2] OR "preexposure prophylaxis"[tiab:~2] OR PREP[tiab] OR "anti‐retroviral chemoprophylaxis"[tiab:~2] OR "antiretroviral chemoprophylaxis"[tiab:~2] OR "Chemoprevention"[Mesh] OR chemoprevention[tiab] OR "HIV prophylaxis"[tiab:~2] | 46,938 |
|  | 3 | "Emtricitabine, Tenofovir Disoproxil Fumarate Drug Combination"[Mesh] OR "emtricitabine tenofovir alafenamide"[Supplementary Concept] | 6,041 |
|  | 4 | "fixed dose emtricitabine tenofovir"[tiab:~2] OR Truvada[tw] OR Descovy[tw] | 1,879 |
|  | 5 | #1 OR #2 OR #3 OR #4 | 74,369 |
| **HIV Prevention** | 6 | "HIV Infections/prevention and control"[Majr] | 42,456 |
|  | 7 | "HIV prevent"[tiab:~5] OR "HIV prevents"[tiab:~5] OR "HIV prevention"[tiab:~5] OR "human immunodeficiency virus prevent"[tiab:~5] OR "human immunodeficiency virus prevents"[tiab:~5] OR "human immunodeficiency virus prevention"[tiab:~5] | 20,830 |
|  | 8 | (#6 OR #7) | 53,796 |
| **Incidence/ Prevalence of PrEP Use** | 9 | "Prevalence"[Mesh] OR "Incidence"[Mesh] OR "statistics and numerical data"[Subheading] | 1,562,385 |
|  | 10 | prevalence*[tiab] OR prevalent[tiab] OR incidence*[tiab] OR incident[tiab] OR demographic*[tiab] OR epidemiolog*[tiab] OR estimate*[tiab] OR frequency[tiab] OR percent*[tiab] OR rate[tiab] OR rates[tiab] OR uptake[tiab] | 11,850,211 |
|  | 11 | #9 OR #10 | 11,854,785 |
| **Sex Workers** | 12 | "Sex Workers"[Mesh] OR "Sex Work"[Mesh] | 12,761 |
|  | 13 | "sex work"[tiab:~1] OR "sex work*"[tw] OR prostitute*[tw] OR prostitution[tw] OR "call girl*"[tw] OR "exchange sex"[tiab:~1] OR "ladies night"[tiab:~1] OR "sex payment"[tiab:~1] OR "survival sex"[tiab:~1] OR "transactional sex"[tiab:~1] OR "walk pavement"[tiab:~1] OR nightwalker*[tw] OR prostitut*[tw] OR sex industr*[tw] OR streetwalker*[tw] OR whore*[tw] | 23,762 |
|  | 14 | #12 OR #13 | 36,054 |
| **MSM** | 15 | "Homosexuality, Male"[Mesh] OR "Bisexuality"[Mesh] OR "Sexual and Gender Minorities"[Mesh] | 41,083 |
|  | 16 | "men who have sex with men"[tiab:~2] OR "men have sex with men"[tiab:~2] OR "men having sex with men"[tiab:~2] OR bisexual*[tw] OR homosexual*[tw] OR MSM[tw] OR "male to male sexual contact"[tw] OR "men sexual relations with men"[tiab:~1] OR "men who have sex with both men and women"[tiab:~1] OR "non binary"[tw] OR "non heterosexual*"[tw] OR bisexual*[tw] OR cruisin[tw] OR cruising*[tw] OR gay[tw] OR gays[tw] OR GBMSM[tw] OR GB-MSM[tw] OR homosexual*[tw] OR lesbigay*[tw] OR lgbt[tw] OR MASM[tw] OR MSM[tw] OR MSMW[tw] OR queer*[tw] | 101,527 |
|  | 17 | #15 OR #16 | 111,889 |
| **Transgender Persons** | 18 | "Transgender Persons"[Mesh] OR "Transsexualism"[Mesh] | 13,863 |
|  | 19 | "trans female*"[tw] OR "trans male*"[tw] OR "trans man"[tiab:~1] OR "trans men"[tiab:~1] OR "trans people"[tiab:~1] OR "trans people"[tiab:~1] OR "trans peoples"[tiab:~1] OR "trans person"[tiab:~1] OR "trans wom*"[tw] OR "transwom*"[tw] OR transman[tw] OR transmen[tw] OR transpeople[tw] OR transperson*[tw] OR "gender reassignment"[tiab:~1] OR "gender variant"[tiab:~1] OR "intersex individual*"[tw] OR "intersex people"[tiab:~1] OR "intersex person"[tiab:~1] OR "sexual dissident"[tiab:~1] OR "trans sexual*"[tw] OR "two spirit"[tiab:~1] OR glbt[tw] OR glbtq[tw] OR lgbt[tw] OR lgbtq[tw] OR lgbtqq[tw] OR transgender*[tw] | 23,384 |
|  | 20 | #18 OR #19 | 23,447 |
| **Drug Users** | 21 | "Drug Users"[Mesh] OR "Substance Abuse, Intravenous"[Mesh] | 4,688 |
|  | 22 | "I V drug user"[tiab:~3] OR "I V drug users"[tiab:~3] OR "injectable drug user"[tiab:~3] OR "injectable drug users"[tiab:~3] OR "intravenous drug user"[tiab:~3] OR "intra-venous drug user"[tiab:~3] OR "intravenous drug users"[tiab:~3] OR "intra-venous drug users"[tiab:~3] OR "IV drug user"[tiab:~3] OR "IV drug users"[tiab:~3] OR "IVDU"[tw] OR "IVDU's"[tiab:~1] OR "IVDUs"[tw] OR "people inject drugs"[tiab:~3] OR "people injected drugs"[tiab:~3] OR "people injecting drugs"[tiab:~3] OR "people who use drugs"[tiab:~3] OR "person inject drugs"[tiab:~3] OR "person who uses drugs"[tiab:~3] OR "persons injecting drugs"[tiab:~3] OR "persons who use drugs"[tiab:~3] OR "PWID"[tw] OR "PWIDs"[tw] OR "PWID's"[tw] OR "PWUD"[tw] OR "PWUDs"[tw] OR "PWUD's"[tw] OR "injecting drug user"[tiab:~1] OR "intravenous drug abuse"[tiab:~1] OR "intravenous drug use"[tiab:~1] OR "intravenous substance abuse"[tiab:~1] OR "intravenous substance misuse"[tiab:~1] OR "intravenous substance use"[tiab:~1] OR "parenteral drug abuse"[tiab:~1] OR "parenteral drug use"[tiab:~1] OR "people who inject drugs"[tiab:~1] OR "person who inject drugs"[tiab:~1] OR "persons who inject drugs"[tiab:~1] OR "injecting drug users"[tiab:~1] OR "intravenous drug abuser"[tiab:~1] OR "intravenous drug user"[tiab:~1] OR "intravenous substance abuser"[tiab:~1] OR "intravenous substance misuser"[tiab:~1] OR "intravenous substance user"[tiab:~1] OR "parenteral drug abuser"[tiab:~1] OR "parenteral drug user"[tiab:~1] OR "intravenous drug abusers"[tiab:~1] OR "intravenous drug users"[tiab:~1] OR "intravenous substance abusers"[tiab:~1] OR "intravenous substance misusers"[tiab:~1] OR "intravenous substance users"[tiab:~1] OR "parenteral drug abusers"[tiab:~1] OR "parenteral drug users"[tiab:~1] OR (((inject*[ti] OR intraven*[ti] OR parenteral[ti] OR use[ti] OR misus*[ti] OR user[ti] OR users[ti]) AND (drug*[ti] OR substance*[ti])) OR ((inject*[ot] OR intraven*[ot] OR parenteral[ot]) AND (drug*[ot] OR substance*[ot]))) | 3,630,377 |
|  | 23 | #21 OR #22 | 3,630,377 |
| **Prisoner Population** | 24 | "Correctional Facilities"[Mesh] OR "Prisons"[Mesh] OR "Jails"[Mesh] OR "Prisoners"[Mesh] | 33,600 |
|  | 25 | "correctional facilities"[tiab:~1] OR "correctional facility"[tiab:~1] OR "correctional institution"[tw] OR "correctional institutions"[tw] OR "gaol"[tw] OR "gaols"[tw] OR "penal facilities"[tiab:~1] OR "penal facility"[tiab:~1] OR "penal institution"[tiab:~1] OR "penal institutions"[tiab:~1] OR custodial[tw] OR imprison*[tw] OR incarcerat*[tw] OR inmate*[tw] OR jail[tw] OR jails[tw] OR penitentiar*[tw] OR prison*[tw] OR "closed setting*"[tw] OR cellmate*[tw] OR confinement*[tw] OR detainee*[tw] OR detention*[tw] OR offender[tw] OR offenders[tw] OR penal[tw] OR penitentiar*[tw] OR reformator*[tw] OR remand*[tw] OR convict*[tw] | 125,682 |
|  | 26 | #24 OR #25 | 125,914 |
| **Serodiscordant Couples** | 27 | serodiscordan*[tw] OR "sero-discordan*"[tw] OR "mixed infection status"[tiab:~2] | 1,879 |
| **All Target Patient Populations** | 28 | #14 OR #17 OR #20 OR #23 OR #26 OR #27 | 3,863,730 |
| **PrEP Use in Target Populations** | 29 | #5 AND #8 AND #11 AND #28 | 3,411 |
| **Restrict to Publication On/After  1st Jan 2017** | 30 | #29 AND (2017/1/1:3000/12/12[pdat]) | 2,539 |
| **Remove Irrelevant Publication Types - Expanded** | 31 | #30 NOT ("Review"[Publication Type] OR "Systematic Review"[Publication Type] OR "Meta-Analysis"[Publication Type] OR "Case Reports" [Publication Type] OR "Published Erratum"[Publication Type] OR "comment"[Publication Type] OR "Editorial"[Publication Type] OR "Controlled Clinical Trial"[Publication Type] OR "Randomized Controlled Trial"[Publication Type] OR "Clinical Trial, Phase III" [Publication Type]) | 1,412 |
| **Animal Studies** | 32 | #31 NOT ("animal cell"[tiab] OR "animal experiment"[tiab] OR "animal health"[tiab] OR "animal model"[tiab] OR "animal tissue"[tiab] OR "bird"[tiab] OR "cancer model"[tiab] OR "canine"[tiab] OR "dog"[tiab] OR "ex vivo study"[tiab] OR "goat"[tiab] OR "Hep-G2 cell line"[tiab] OR "in vitro study"[tiab] OR "knockout mouse"[tiab] OR "Kupffer cell"[tiab] OR "mammal"[tiab] OR "model"[tiab] OR "mouse"[tiab] OR "mouse model"[tiab] OR "murine"[tiab] OR mice[tiab] OR "nonhuman"[tiab] OR "nude rat"[tiab] OR "rat"[tiab] OR "rat model"[tiab] OR "tumor cell"[tiab] OR "tumor model"[tiab] OR "tumor xenograft"[tiab] OR "veterinary"[tiab]) | 1,205 |
| **World Bank 'High-Income' Countries** | 33 | "Andorra"[Mesh] OR "Antigua and Barbuda"[Mesh] OR "Antigua Barbuda"[tiab:~1] OR "Aruba"[Mesh] OR "Asia"[Mesh] OR "Australia"[Mesh] OR "Austria"[Mesh] OR "Bahamas"[Mesh] OR "Bahrain"[Mesh] OR "Barbados"[Mesh] OR "Belgium"[Mesh] OR "Bermuda"[Mesh] OR "British Virgin Islands"[Mesh] OR "Brunei"[Mesh] OR "Canada"[Mesh] OR "Cayman Island*"[tw] OR "Channel Islands"[Mesh] OR "Chile"[Mesh] OR "Cook Island*"[tw] OR "Croatia"[Mesh] OR "Curacao"[Mesh] OR "Cyprus"[Mesh] OR "Czech Republic"[Mesh] OR "Czech Republic*"[tw] OR "Denmark"[Mesh] OR "England"[Mesh] OR "Estonia"[Mesh] OR "Europe"[Mesh] OR "Faroe Island*"[tw] OR "Finland"[Mesh] OR "France"[Mesh] OR "French Polynesia*"[tw] OR "Germany"[Mesh] OR "Gibraltar"[Mesh] OR "Great Britain"[tiab:~1] OR "Great Britain"[tiab:~1] OR "Greece"[Mesh] OR "Greenland"[Mesh] OR "Guam"[Mesh] OR "Guernsey"[Mesh] OR "Hong Kong*"[tw] OR "Hungary"[Mesh] OR "Iceland"[Mesh] OR "Ireland"[Mesh] OR "Isle of Man"[tw] OR "Israel"[Mesh] OR "Italy"[Mesh] OR "Japan"[Mesh] OR "Jersey Channel Island*"[tw] OR "Kuwait"[Mesh] OR "Latvia"[Mesh] OR "Liechtenstein"[Mesh] OR "Lithuania"[Mesh] OR "Luxembourg"[Mesh] OR "Macau"[Mesh] OR "Malta"[Mesh] OR "Monaco"[Mesh] OR "Netherlands"[Mesh] OR "New Caledonia"[Mesh] OR "New Zealand"[Mesh] OR "New Zealand*"[tw] OR "Northern Mariana Island*"[tw] OR "Norway"[Mesh] OR "Oman"[Mesh] OR "Panama"[Mesh] OR "Poland"[Mesh] OR "Polynesia"[Mesh] OR "Portugal"[Mesh] OR "Puerto Rico"[Mesh] OR "Qatar"[Mesh] OR "Republic of Korea"[Mesh] OR "Romania"[Mesh] OR "Saint Kitts and Nevis"[Mesh] OR "Saint Kitts Nevis"[tiab:~1] OR "Saint Maarten*"[tw] OR "Saint Martin*"[tw] OR "San Marino"[Mesh] OR "San Marino*"[tw] OR "Saudi Arabia"[Mesh] OR "Saudi Arabia*"[tw] OR "Scotland"[Mesh] OR "Seychelles"[Mesh] OR "Singapore"[Mesh] OR "Slovakia"[Mesh] OR "Slovenia"[Mesh] OR "Spain"[Mesh] OR "Sweden"[Mesh] OR "Switzerland"[Mesh] OR "Taiwan"[Mesh] OR "Thailand"[Mesh] OR "Trinidad and Tobago"[Mesh] OR "Trinidad Tobago"[tiab:~1] OR "Turks Caicos"[tiab:~1] OR "U K"[tw] OR "UK"[tw] OR "United Arab Emirates"[Mesh] OR "United Arab Emirates"[tw] OR "United Kingdom"[Mesh] OR "United Kingdom"[tw] OR "United States Virgin Islands"[Mesh] OR "Uruguay"[Mesh] OR "Virgin Island*"[tw] OR Andorra*[tw] OR Aruba*[tw] OR Australia*[tw] OR Austria*[tw] OR Bahama*[tw] OR Bahrain*[tw] OR Barbados*[tw] OR Belgian*[tw] OR Belgium[tw] OR Bermuda*[tw] OR Britain[tw] OR British[tw] OR Brunei*[tw] OR Canada[tw] OR Canadian*[tw] OR Chile*[tw] OR Croatia*[tw] OR Curaçao*[tw] OR Cyprus*[tw] OR Dane*[tw] OR Danish[tw] OR Denmark[tw] OR Dutch*[tw] OR England[tw] OR English[tw] OR Estonia*[tw] OR Finland[tw] OR Finn*[tw] OR France[tw] OR French*[tw] OR German*[tw] OR Gibraltar*[tw] OR Greece*[tw] OR Greenland*[tw] OR Guam*[tw] OR Guernsey*[tw] OR Hungary*[tw] OR Iceland*[tw] OR Ireland[tw] OR Irish*[tw] OR Israel*[tw] OR Italian*[tw] OR Italy[tw] OR Japan*[tw] OR Korea*[tw] OR Kuwait*[tw] OR Latvia*[tw] OR Liechtenstein*[tw] OR Lithuania*[tw] OR Luxembourg*[tw] OR Macao*[tw] OR Malta*[tw] OR Monaco*[tw] OR Nauru*[tw] OR Netherlands[tw] OR New Caledonia*[tw] OR Norway[tw] OR Norwegian*[tw] OR Oman*[tw] OR Panama*[tw] OR Poland[tw] OR Pole*[tw] OR Polish[tw] OR Portugal[tw] OR Portuguese[tw] OR Puerto Rico*[tw] OR Qatar*[tw] OR Romania*[tw] OR Scot*[tw] OR Scotland[tw] OR Seychelle*[tw] OR Singapore*[tw] OR Slovakia*[tw] OR Slovenia*[tw] OR Spain[tw] OR Spaniard*[tw] OR Spanish[tw] OR Sweden[tw] OR Swedish[tw] OR Swiss[tw] OR Switzerland[tw] OR Taiwan*[tw] OR Thai*[tw] OR Uruguay*[tw] | 6,030,636 |
| **UK-Filter** | 34 | "United Kingdom"[Mesh] | 484,634 |
|  | 35 | ("national health service*"[tiab] OR nhs[tiab] OR "national health service*"[ad] OR nhs[ad]) | 526,717 |
|  | 36 | (english[tiab] NOT ("published english"[tiab:~5] OR "publication english"[tiab:~5] OR "translate english"[tiab:~5] OR "translated english"[tiab:~5] OR "written english"[tiab:~5] OR "language english"[tiab:~5] OR "speak english"[tiab:~5] OR "literature english"[tiab:~5] OR "citation english"[tiab:~5])) | 43,464,662 |
|  | 37 | (gb[tiab] OR "g.b."[tiab] OR britain*[tiab] OR (british*[tiab] NOT "british columbia"[tiab]) OR uk[tiab] OR "u.k."[tiab] OR united kingdom*[tiab] OR (england*[tiab] NOT "new england"[tiab:~0]) OR northern ireland*[tiab] OR northern irish*[tiab] OR scotland*[tiab] OR scottish*[tiab] OR ((wales[tiab] OR "south wales"[tiab:~0]) NOT "new south wales"[tiab:~0]) OR welsh*[tiab]) | 222,768 |
|  | 38 | (bath[tiab] OR "bath's"[tiab] OR ((birmingham[tiab] NOT alabama*[tiab]) OR ("birmingham's"[tiab] NOT alabama*[tiab]) OR bradford[tiab] OR "bradford's"[tiab] OR brighton[tiab] OR "brighton's"[tiab] OR bristol[tiab] OR "bristol's"[tiab] OR carlisle*[tiab] OR "carlisle's"[tiab] OR (cambridge[tiab] NOT (massachusetts*[tiab] OR boston*[tiab] OR harvard*[tiab])) OR ("cambridge's"[tiab] NOT (massachusetts*[tiab] OR boston*[tiab] OR harvard*[tiab])) OR (canterbury[tiab] NOT zealand*[tiab]) OR ("canterbury's"[tiab] NOT zealand*[tiab]) OR chelmsford[tiab] OR "chelmsford's"[tiab] OR chester[tiab] OR "chester's"[tiab] OR chichester[tiab] OR "chichester's"[tiab] OR coventry[tiab] OR "coventry's"[tiab] OR derby[tiab] OR "derby's"[tiab] OR (durham[tiab] NOT (carolina*[tiab] OR nc[tiab])) OR ("durham's"[tiab] NOT (carolina*[tiab] OR nc[tiab])) OR ely[tiab] OR "ely's"[tiab] OR exeter[tiab] OR "exeter's"[tiab] OR gloucester[tiab] OR "gloucester's"[tiab] OR hereford[tiab] OR "hereford's"[tiab] OR hull[tiab] OR "hull's"[tiab] OR lancaster[tiab] OR "lancaster's"[tiab] OR leeds*[tiab] OR leicester[tiab] OR "leicester's"[tiab] OR (lincoln[tiab] NOT nebraska*[tiab]) OR ("lincoln's"[tiab] NOT nebraska*[tiab]) OR (liverpool[tiab] NOT (new south wales*[tiab] OR nsw[tiab])) OR ("liverpool's"[tiab] NOT (new south wales*[tiab] OR nsw[tiab])) OR ((london[tiab] NOT (ontario*[tiab] OR ont[tiab] OR toronto*[tiab])) OR ("london's"[tiab] NOT (ontario*[tiab] OR ont[tiab] OR toronto*[tiab])) OR manchester[tiab] OR "manchester's"[tiab] OR (newcastle[tiab] NOT (new south wales*[tiab] OR nsw[tiab])) OR ("newcastle's"[tiab] NOT (new south wales*[tiab] OR nsw[tiab])) OR norwich[tiab] OR "norwich's"[tiab] OR nottingham[tiab] OR "nottingham's"[tiab] OR oxford[tiab] OR "oxford's"[tiab] OR peterborough[tiab] OR "peterborough's"[tiab] OR plymouth[tiab] OR "plymouth's"[tiab] OR portsmouth[tiab] OR "portsmouth's"[tiab] OR preston[tiab] OR "preston's"[tiab] OR ripon[tiab] OR "ripon's"[tiab] OR salford[tiab] OR "salford's"[tiab] OR salisbury[tiab] OR "salisbury's"[tiab] OR sheffield[tiab] OR "sheffield's"[tiab] OR southampton[tiab] OR "southampton's"[tiab] OR st albans[tiab] OR stoke[tiab] OR "stoke's"[tiab] OR sunderland[tiab] OR "sunderland's"[tiab] OR truro[tiab] OR "truro's"[tiab] OR wakefield[tiab] OR "wakefield's"[tiab] OR wells[tiab] OR westminster[tiab] OR "westminster's"[tiab] OR winchester[tiab] OR "winchester's"[tiab] OR wolverhampton[tiab] OR "wolverhampton's"[tiab] OR (worcester[tiab] NOT (massachusetts*[tiab] OR boston*[tiab] OR harvard*[tiab])) OR ("worcester's"[tiab] NOT (massachusetts*[tiab] OR boston*[tiab] OR harvard*[tiab])) OR (york[tiab] NOT ("new york*"[tiab] OR ny[tiab] OR ontario*[tiab] OR ont[tiab] OR toronto*[tiab])) OR ("york's"[tiab] NOT ("new york*"[tiab] OR ny[tiab] OR ontario*[tiab] OR ont[tiab] OR toronto*[tiab]))))) | 1,860,950 |
|  | 39 | (bangor[tiab] OR "bangor's"[tiab] OR cardiff[tiab] OR "cardiff's"[tiab] OR newport[tiab] OR "newport's"[tiab] OR "st asaph"[tiab] OR "st asaph's"[tiab] OR st davids[tiab] OR swansea[tiab] OR "swansea's"[tiab]) | 22,381 |
|  | 40 | (aberdeen[tiab] OR "aberdeen's"[tiab] OR dundee[tiab] OR "dundee's"[tiab] OR edinburgh[tiab] OR "edinburgh's"[tiab] OR glasgow[tiab] OR "glasgow's"[tiab] OR inverness[tiab] OR (perth[tiab] NOT australia*[tiab]) OR ("perth's"[tiab] NOT australia*[tiab]) OR stirling[tiab] OR "stirling's"[tiab]) | 567,702 |
|  | 41 | (armagh[tiab] OR "armagh's"[tiab] OR belfast[tiab] OR "belfast's"[tiab] OR lisburn[tiab] OR "lisburn's"[tiab] OR londonderry[tiab] OR "londonderry's"[tiab] OR derry[tiab] OR "derry's"[tiab] OR newry[tiab] OR "newry's"[tiab]) | 61,970 |
|  | 42 | #34 OR #35 OR #36 OR #37 OR #38 OR #39 OR #40 OR #41 | 43,465,229 |
| **Canada-Filter** | 43 | Canada[Mesh] OR Canada[tw] OR Canad*[tw] | 1,972,009 |
|  | 44 | ("British Columbia"[tiab] OR "Colombie Britannique"[tiab] OR Alberta*[tiab] OR Saskatchewan[tiab] OR Manitoba*[tiab] OR Ontario[tiab] OR Quebec[tiab] OR "Nouveau Brunswick"[tiab] OR "New Brunswick"[tiab] OR "Nova Scotia"[tiab] OR "Nouvelle Ecosse"[tiab] OR "Prince Edward Island"[tiab] OR Newfoundland[tiab] OR Labrador[tiab] OR Nunavut[tiab] OR NWT[tiab] OR "Northwest Territories"[tiab] OR Yukon[tiab] OR Nunavik[tiab] OR Inuvialuit[tiab]) | 724,265 |
|  | 45 | Abbotsford[tiab] OR "Ajax"[tiab] OR "Aurora"[tiab] OR "Barrie"[tiab] OR "Belleville"[tiab] OR "Blainville"[tiab] OR "Brampton"[tiab] OR "Brantford"[tiab] OR "Brossard"[tiab] OR "Burlington"[tiab] OR "Burnaby"[tiab] OR "Caledon"[tiab] OR "Calgary"[tiab] OR "cape breton"[tiab] OR "chatham kent"[tiab] OR "Chilliwack"[tiab] OR "Clarington"[tiab] OR "Coquitlam"[tiab] OR "Drummondville"[tiab] OR "Edmonton"[tiab] OR "Fredericton"[tiab] OR "fort mcmurray"[tiab] OR "Gatineau"[tiab] OR "Granby"[tiab] OR "grande prairie"[tiab] OR "Guelph"[tiab] OR ("Halton Hills"[tiab]) OR "Iqaluit"[tiab] OR "Inuvik"[tiab] OR "Kamloops"[tiab] OR "kawartha lakes"[tiab] OR "Kelowna"[tiab] OR "Kingston"[tiab] OR "Kitchener"[tiab] OR "Langley"[tiab] OR "Laval"[tiab] OR "Lethbridge"[tiab] OR "Levis"[tiab] OR "Longueuil"[tiab] OR "maple ridge"[tiab] OR "Markham"[tiab] OR "medicine hat"[tiab] OR "Milton"[tiab] OR "Mirabel"[tiab] OR "Mississauga"[tiab] OR "Moncton"[tiab] OR "Montreal"[tiab] OR "Nanaimo"[tiab] OR "new westminster"[tiab] OR "Newmarket"[tiab] OR "niagara falls"[tiab] OR "norfolk county"[tiab] OR "north bay"[tiab] OR "north vancouver"[tiab] OR "north vancouver"[tiab] OR "Oakville"[tiab] OR "Oshawa"[tiab] OR "Ottawa"[tiab] OR "port coquitlam"[tiab] OR "prince george"[tiab] OR "quebec city"[tiab] OR "red deer"[tiab] OR "Regina"[tiab] OR "Repentigny"[tiab] OR "Saanich"[tiab] OR "Saguenay"[tiab] OR "saint john"[tiab] OR "Saint-Hyacinthe"[tiab] OR "Saint-Jean-sur-Richelieu"[tiab] OR "Saint-Jerome"[tiab] OR "Sarnia"[tiab] OR "Saskatoon"[tiab] OR "sault ste marie"[tiab] OR "Sherbrooke"[tiab] OR "st albert"[tiab] OR "st catharines"[tiab] OR "st john s"[tiab] OR ("Strathcona County"[tiab:~2]) OR "Terrebonne"[tiab] OR "thunder bay"[tiab] OR "Toronto"[tiab] OR "Trois-Rivieres"[tiab] OR "Vancouver"[tiab] OR "Vaughan"[tiab] OR (("Airdrie"[tiab] OR "Cambridge"[tiab] OR ("Halifax"[tiab] OR "Hamilton"[tiab] OR "London"[tiab] OR "Peterborough"[tiab] OR "Pickering"[tiab] OR "Richmond"[tiab] OR "richmond hill"[tiab] OR "Sudbury"[tiab] OR "Surrey"[tiab] OR "Victoria"[tiab] OR "Waterloo"[tiab] OR "Welland"[tiab] OR "Whitby"[tiab] OR "Windsor"[tiab])) NOT ("UK"[tw] OR "Britain"[tw] OR "united kingdom"[tw] OR "England"[tw] OR "Australia"[tw])) OR "Whitehorse"[tiab] OR "Winnipeg"[tiab] OR "wood buffalo"[tiab] OR "Yellowknife"[tiab] | 2,502,895 |
|  | 46 | #43 OR #44 OR #45 | 2,988,916 |
| **Spain-Filter** | 47 | ((spain OR espagne OR espana OR spagna) OR (spain[ad] OR espagne[ad] OR espana[ad] OR spanien[ad] OR spagna[ad]) OR (catalunya[ad] OR catalonia[ad] OR catalogne[ad] OR cataluna[ad] OR catala[ad] OR barcelon*[ad] OR tarragona[ad] OR lleida[ad] OR lerida[ad] OR girona[ad] OR gerona[ad] OR sabadell[ad] OR hospitalet[ad] OR l’hospitalet[ad]) OR (valencia*[ad] OR castello*[ad] OR alacant[ad] OR alicant*[ad]) OR (murcia*[ad] OR (cartagen*[ad] NOT indias[ad])) OR (andalu*[ad] OR sevill*[ad] OR granad*[ad] OR huelva[ad] OR almeria[ad] OR cadiz[ad] OR jaen[ad] OR malaga[ad] OR (cordoba[ad] NOT argentin*[ad])) OR (extremadura[ad] OR caceres[ad] OR badajoz[ad] OR madrid[ad]) OR (castilla[ad] OR salamanca[ad] OR zamora[ad] OR valladolid[ad] OR segovia[ad] OR soria[ad] OR palencia[ad] OR avila[ad] OR burgos[ad]) OR (leon[ad] NOT (france[ad] OR clermont[ad] OR rennes[ad] OR lyon[ad] OR USA[ad] OR mexic*[ad])) OR (galicia[ad] OR gallego[ad] OR compostela[ad] OR vigo[ad] OR corun*[ad] OR ferrol[ad] OR orense[ad] OR ourense[ad] OR pontevedra[ad] OR lugo[ad]) OR (oviedo[ad] OR gijon[ad] OR asturia*[ad]) OR (cantabr*[ad] OR santander[ad]) OR (vasco[ad] OR euskadi[ad] OR basque[ad] OR bilbao[ad] OR bilbo[ad] OR donosti*[ad] OR san sebastian[ad] OR vizcaya[ad] OR bizkaia[ad] OR guipuzcoa[ad] OR gipuzkoa[ad] OR alava[ad] OR araba[ad] OR vitoria[ad] OR gasteiz[AD]) OR (navarr*[ad] OR nafarroa[ad] OR pamplona[ad] OR iruna[ad] OR irunea[ad]) OR (logron*[ad] OR rioj*[ad]) OR (aragon*[ad] OR zaragoza[ad] OR teruel[ad] OR huesca[ad]) OR (mancha[ad] OR ciudad real[ad] OR albacete[ad] OR cuenca[ad]) OR (toledo[ad] NOT (ohio[ad] OR us[ad] OR usa[ad] OR OH[ad])) OR (guadalajara[ad] NOT mexic*[ad]) OR (balear*[ad] OR mallorca[ad] OR menorca[ad] OR ibiza[ad] OR eivissa[ad]) OR (palmas[ad] OR lanzarote[ad] OR canari*[ad] OR tenerif*[ad]) OR (ceuta[ad] OR melilla[ad])) OR (osasunbide*[ad] OR osakidetza[ad] OR insalud[ad] OR sergas[ad] OR catsalut[ad] OR sespa[ad] OR osasunbidea[ad] OR imsalud[ad] OR sescam[ad] OR ib-salut[ad]) | 1,655,396 |
| **Restrict to World Bank 'High-Income' Countries** | 48 | #32 AND (#33 OR #42 OR #46 OR #47) | 1,205 |
| **Restrict to Non-US /  Non-African countries** | 49 | #48 NOT ((africa[mesh] OR north america[mesh:noexp] OR united states[mesh]) NOT (asia[mesh] OR australia[mesh] OR canada[mesh] OR europe[mesh] OR south america[mesh])) | **695** |

# **Supplementary Table 2b. Search Terms for Prevalence of PrEP Use Sources (PubMed)**

| **Term Group** | **#** | **Search Terms** | **Number of Hits  (July 2023)** |
| --- | --- | --- | --- |
| **Pre-Exposure Prophylaxis (PrEP)** | 1 | "Pre-Exposure Prophylaxis"[Mesh] OR "Chemoprevention"[Mesh] | 27,430 |
|  | 2 | "pre‐exposure prophylaxis"[tiab:~2] OR "preexposure prophylaxis"[tiab:~2] OR PREP[tiab] OR "anti‐retroviral chemoprophylaxis"[tiab:~2] OR "antiretroviral chemoprophylaxis"[tiab:~2] OR "Chemoprevention"[Mesh] OR chemoprevention[tiab] OR "HIV prophylaxis"[tiab:~2] | 44,466 |
|  | 3 | "Emtricitabine, Tenofovir Disoproxil Fumarate Drug Combination"[Mesh] OR "emtricitabine tenofovir alafenamide"[Supplementary Concept] OR "cabotegravir" [Supplementary Concept] | 497 |
|  | 4 | "fixed dose emtricitabine tenofovir"[tiab:~1] OR "cabotegravir extended-release"[tiab:~1] OR Truvada[tw] OR Descovy[tw] OR Apretude | 679 |
|  | 5 | #1 OR #2 OR #3 OR #4 | 45,599 |
| **HIV Prevention** | 6 | "HIV Infections/prevention and control"[Majr] | 37,802 |
|  | 7 | "HIV prevent"[tiab:~5] OR "HIV prevents"[tiab:~5] OR "HIV prevention"[tiab:~5] OR "human immunodeficiency virus prevent"[tiab:~5] OR "human immunodeficiency virus prevents"[tiab:~5] OR "human immunodeficiency virus prevention"[tiab:~5] | 32,818 |
|  | 8 | (#6 OR #7) | 56,917 |
| **Incidence/ Prevalence of PrEP Use** | 9 | "Prevalence"[Mesh] OR "Incidence"[Mesh] OR "statistics and numerical data"[Subheading] | 3,371,202 |
|  | 10 | prevalence*[tiab] OR prevalent[tiab] OR incidence*[tiab] OR incident[tiab] OR demographic*[tiab] OR epidemiolog*[tiab] OR estimate*[tiab] OR frequency[tiab] OR percent*[tiab] OR rate[tiab] OR rates[tiab] OR uptake[tiab] | 7,459,837 |
|  | 11 | #9 OR #10 | 9,037,610 |
| **Sex Workers** | 12 | "Sex Workers"[Mesh] OR "Sex Work"[Mesh] | 8,715 |
|  | 13 | "sex work"[tiab:~1] OR "sex work*"[tw] OR prostitute*[tw] OR prostitution[tw] OR "call girl*"[tw] OR "exchange sex"[tiab:~1] OR "ladies night"[tiab:~1] OR "sex payment"[tiab:~1] OR "survival sex"[tiab:~1] OR "transactional sex"[tiab:~1] OR "walk pavement"[tiab:~1] OR nightwalker*[tw] OR prostitut*[tw] OR sex industr*[tw] OR streetwalker*[tw] OR whore*[tw] | 16,443 |
|  | 14 | #12 OR #13 | 16,443 |
| **MSM** | 15 | "Homosexuality, Male"[Mesh] OR "Bisexuality"[Mesh] OR "Sexual and Gender Minorities"[Mesh] | 33,248 |
|  | 16 | "men who have sex with men"[tiab:~2] OR "men have sex with men"[tiab:~2] OR "men having sex with men"[tiab:~2] OR bisexual*[tw] OR homosexual*[tw] OR MSM[tw] OR "male to male sexual contact"[tw] OR "men sexual relations with men"[tiab:~1] OR "men who have sex with both men and women"[tiab:~1] OR "non binary"[tw] OR "non heterosexual*"[tw] OR bisexual*[tw] OR cruisin[tw] OR cruising*[tw] OR gay[tw] OR gays[tw] OR GBMSM[tw] OR GB-MSM[tw] OR homosexual*[tw] OR lesbigay*[tw] OR lgbt[tw] OR MASM[tw] OR MSM[tw] OR MSMW[tw] OR queer*[tw] | 58,462 |
|  | 17 | #15 OR #16 | 64,807 |
| **Transgender Persons** | 18 | "Transgender Persons"[Mesh] OR "Transsexualism"[Mesh] | 10,091 |
|  | 19 | "trans female*"[tw] OR "trans male*"[tw] OR "trans man"[tiab:~1] OR "trans men"[tiab:~1] OR "trans people"[tiab:~1] OR "trans people"[tiab:~1] OR "trans peoples"[tiab:~1] OR "trans person"[tiab:~1] OR "trans wom*"[tw] OR "transwom*"[tw] OR transman[tw] OR transmen[tw] OR transpeople[tw] OR transperson*[tw] OR "gender reassignment"[tiab:~1] OR "gender variant"[tiab:~1] OR "intersex individual*"[tw] OR "intersex people"[tiab:~1] OR "intersex person"[tiab:~1] OR "sexual dissident"[tiab:~1] OR "trans sexual*"[tw] OR "two spirit"[tiab:~1] OR glbt[tw] OR glbtq[tw] OR lgbt[tw] OR lgbtq[tw] OR lgbtqq[tw] OR transgender*[tw] | 16,117 |
|  | 20 | #18 OR #19 | 18,581 |
| **Drug Users** | 21 | "Drug Users"[Mesh] OR "Substance Abuse, Intravenous"[Mesh] | 19,401 |
|  | 22 | "I V drug user"[tiab:~3] OR "I V drug users"[tiab:~3] OR "injectable drug user"[tiab:~3] OR "injectable drug users"[tiab:~3] OR "intravenous drug user"[tiab:~3] OR "intra-venous drug user"[tiab:~3] OR "intravenous drug users"[tiab:~3] OR "intra-venous drug users"[tiab:~3] OR "IV drug user"[tiab:~3] OR "IV drug users"[tiab:~3] OR "IVDU"[tw] OR "IVDU's"[tiab:~1] OR "IVDUs"[tw] OR "people inject drugs"[tiab:~3] OR "people injected drugs"[tiab:~3] OR "people injecting drugs"[tiab:~3] OR "people who use drugs"[tiab:~3] OR "person inject drugs"[tiab:~3] OR "person who uses drugs"[tiab:~3] OR "persons injecting drugs"[tiab:~3] OR "persons who use drugs"[tiab:~3] OR "PWID"[tw] OR "PWIDs"[tw] OR "PWID's"[tw] OR "PWUD"[tw] OR "PWUDs"[tw] OR "PWUD's"[tw] OR "injecting drug user"[tiab:~1] OR "intravenous drug abuse"[tiab:~1] OR "intravenous drug use"[tiab:~1] OR "intravenous substance abuse"[tiab:~1] OR "intravenous substance misuse"[tiab:~1] OR "intravenous substance use"[tiab:~1] OR "parenteral drug abuse"[tiab:~1] OR "parenteral drug use"[tiab:~1] OR "people who inject drugs"[tiab:~1] OR "person who inject drugs"[tiab:~1] OR "persons who inject drugs"[tiab:~1] OR "injecting drug users"[tiab:~1] OR "intravenous drug abuser"[tiab:~1] OR "intravenous drug user"[tiab:~1] OR "intravenous substance abuser"[tiab:~1] OR "intravenous substance misuser"[tiab:~1] OR "intravenous substance user"[tiab:~1] OR "parenteral drug abuser"[tiab:~1] OR "parenteral drug user"[tiab:~1] OR "intravenous drug abusers"[tiab:~1] OR "intravenous drug users"[tiab:~1] OR "intravenous substance abusers"[tiab:~1] OR "intravenous substance misusers"[tiab:~1] OR "intravenous substance users"[tiab:~1] OR "parenteral drug abusers"[tiab:~1] OR "parenteral drug users"[tiab:~1] OR (((inject*[ti] OR intraven*[ti] OR parenteral[ti] OR use[ti] OR misus*[ti] OR user[ti] OR users[ti]) AND (drug*[ti] OR substance*[ti])) OR ((inject*[ot] OR intraven*[ot] OR parenteral[ot]) AND (drug*[ot] OR substance*[ot]))) | 72,690 |
|  | 23 | #21 OR #22 | 79,777 |
| **Prisoner Population** | 24 | "Correctional Facilities"[Mesh] OR "Prisons"[Mesh] OR "Jails"[Mesh] OR "Prisoners"[Mesh] | 26,011 |
|  | 25 | "correctional facilities"[tiab:~1] OR "correctional facility"[tiab:~1] OR "correctional institution"[tw] OR "correctional institutions"[tw] OR "gaol"[tw] OR "gaols"[tw] OR "penal facilities"[tiab:~1] OR "penal facility"[tiab:~1] OR "penal institution"[tiab:~1] OR "penal institutions"[tiab:~1] OR custodial[tw] OR imprison*[tw] OR incarcerat*[tw] OR inmate*[tw] OR jail[tw] OR jails[tw] OR penitentiar*[tw] OR prison*[tw] OR "closed setting*"[tw] OR cellmate*[tw] OR confinement*[tw] OR detainee*[tw] OR detention*[tw] OR offender[tw] OR offenders[tw] OR penal[tw] OR penitentiar*[tw] OR reformator*[tw] OR remand*[tw] OR convict*[tw] | 91,727 |
|  | 26 | #24 OR #25 | 92,156 |
| **Serodiscordant Couples** | 27 | serodiscordan*[tw] OR "sero-discordan*"[tw] OR "mixed infection status"[tiab:~2] | 1,383 |
| **All Target Patient Populations** | 28 | #14 OR #17 OR #20 OR #23 OR #26 OR #27 | 246,656 |
| **PrEP Use in Target Populations** | 29 | #5 AND #8 AND #11 AND #28 | 2,592 |
| **Restrict to Publication On/After  1st Jan 2017** | 30 | #29 AND (2017/1/1:3000/12/12[pdat]) | 2,112 |
| **Remove Irrelevant Publication Types - Expanded** | 31 | #30 NOT ("Review"[Publication Type] OR "Systematic Review"[Publication Type] OR "Meta-Analysis"[Publication Type] OR "Case Reports" [Publication Type] OR "Published Erratum"[Publication Type] OR "comment"[Publication Type] OR "Editorial"[Publication Type] OR "Controlled Clinical Trial"[Publication Type] OR "Randomized Controlled Trial"[Publication Type] OR "Clinical Trial, Phase III" [Publication Type]) | 1,840 |
| **Animal Studies** | 32 | #31 NOT ("animal cell"[tiab] OR "animal experiment"[tiab] OR "animal health"[tiab] OR "animal model"[tiab] OR "animal tissue"[tiab] OR "bird"[tiab] OR "cancer model"[tiab] OR "canine"[tiab] OR "dog"[tiab] OR "ex vivo study"[tiab] OR "goat"[tiab] OR "Hep-G2 cell line"[tiab] OR "in vitro study"[tiab] OR "knockout mouse"[tiab] OR "Kupffer cell"[tiab] OR "mammal"[tiab] OR "model"[tiab] OR "mouse"[tiab] OR "mouse model"[tiab] OR "murine"[tiab] OR mice[tiab] OR "nonhuman"[tiab] OR "nude rat"[tiab] OR "rat"[tiab] OR "rat model"[tiab] OR "tumor cell"[tiab] OR "tumor model"[tiab] OR "tumor xenograft"[tiab] OR "veterinary"[tiab]) | 1,520 |
| **World Bank 'High-Income' Countries** | 33 | "Andorra"[Mesh] OR "Antigua and Barbuda"[Mesh] OR "Antigua Barbuda"[tiab:~1] OR "Aruba"[Mesh] OR "Asia"[Mesh] OR "Australia"[Mesh] OR "Austria"[Mesh] OR "Bahamas"[Mesh] OR "Bahrain"[Mesh] OR "Barbados"[Mesh] OR "Belgium"[Mesh] OR "Bermuda"[Mesh] OR "British Virgin Islands"[Mesh] OR "Brunei"[Mesh] OR "Canada"[Mesh] OR "Cayman Island*"[tw] OR "Channel Islands"[Mesh] OR "Chile"[Mesh] OR "Cook Island*"[tw] OR "Croatia"[Mesh] OR "Curacao"[Mesh] OR "Cyprus"[Mesh] OR "Czech Republic"[Mesh] OR "Czech Republic*"[tw] OR "Denmark"[Mesh] OR "England"[Mesh] OR "Estonia"[Mesh] OR "Europe"[Mesh] OR "Faroe Island*"[tw] OR "Finland"[Mesh] OR "France"[Mesh] OR "French Polynesia*"[tw] OR "Germany"[Mesh] OR "Gibraltar"[Mesh] OR "Great Britain"[tiab:~1] OR "Great Britain"[tiab:~1] OR "Greece"[Mesh] OR "Greenland"[Mesh] OR "Guam"[Mesh] OR "Guernsey"[Mesh] OR "Hong Kong*"[tw] OR "Hungary"[Mesh] OR "Iceland"[Mesh] OR "Ireland"[Mesh] OR "Isle of Man"[tw] OR "Israel"[Mesh] OR "Italy"[Mesh] OR "Japan"[Mesh] OR "Jersey Channel Island*"[tw] OR "Kuwait"[Mesh] OR "Latvia"[Mesh] OR "Liechtenstein"[Mesh] OR "Lithuania"[Mesh] OR "Luxembourg"[Mesh] OR "Macau"[Mesh] OR "Malta"[Mesh] OR "Monaco"[Mesh] OR "Netherlands"[Mesh] OR "New Caledonia"[Mesh] OR "New Zealand"[Mesh] OR "New Zealand*"[tw] OR "Northern Mariana Island*"[tw] OR "Norway"[Mesh] OR "Oman"[Mesh] OR "Panama"[Mesh] OR "Poland"[Mesh] OR "Polynesia"[Mesh] OR "Portugal"[Mesh] OR "Puerto Rico"[Mesh] OR "Qatar"[Mesh] OR "Republic of Korea"[Mesh] OR "Romania"[Mesh] OR "Saint Kitts and Nevis"[Mesh] OR "Saint Kitts Nevis"[tiab:~1] OR "Saint Maarten*"[tw] OR "Saint Martin*"[tw] OR "San Marino"[Mesh] OR "San Marino*"[tw] OR "Saudi Arabia"[Mesh] OR "Saudi Arabia*"[tw] OR "Scotland"[Mesh] OR "Seychelles"[Mesh] OR "Singapore"[Mesh] OR "Slovakia"[Mesh] OR "Slovenia"[Mesh] OR "Spain"[Mesh] OR "Sweden"[Mesh] OR "Switzerland"[Mesh] OR "Taiwan"[Mesh] OR "Thailand"[Mesh] OR "Trinidad and Tobago"[Mesh] OR "Trinidad Tobago"[tiab:~1] OR "Turks Caicos"[tiab:~1] OR "U K"[tw] OR "UK"[tw] OR "United Arab Emirates"[Mesh] OR "United Arab Emirates"[tw] OR "United Kingdom"[Mesh] OR "United Kingdom"[tw] OR "United States Virgin Islands"[Mesh] OR "Uruguay"[Mesh] OR "Virgin Island*"[tw] OR Andorra*[tw] OR Aruba*[tw] OR Australia*[tw] OR Austria*[tw] OR Bahama*[tw] OR Bahrain*[tw] OR Barbados*[tw] OR Belgian*[tw] OR Belgium[tw] OR Bermuda*[tw] OR Britain[tw] OR British[tw] OR Brunei*[tw] OR Canada[tw] OR Canadian*[tw] OR Chile*[tw] OR Croatia*[tw] OR Curaçao*[tw] OR Cyprus*[tw] OR Dane*[tw] OR Danish[tw] OR Denmark[tw] OR Dutch*[tw] OR England[tw] OR English[tw] OR Estonia*[tw] OR Finland[tw] OR Finn*[tw] OR France[tw] OR French*[tw] OR German*[tw] OR Gibraltar*[tw] OR Greece*[tw] OR Greenland*[tw] OR Guam*[tw] OR Guernsey*[tw] OR Hungary*[tw] OR Iceland*[tw] OR Ireland[tw] OR Irish*[tw] OR Israel*[tw] OR Italian*[tw] OR Italy[tw] OR Japan*[tw] OR Korea*[tw] OR Kuwait*[tw] OR Latvia*[tw] OR Liechtenstein*[tw] OR Lithuania*[tw] OR Luxembourg*[tw] OR Macao*[tw] OR Malta*[tw] OR Monaco*[tw] OR Nauru*[tw] OR Netherlands[tw] OR New Caledonia*[tw] OR Norway[tw] OR Norwegian*[tw] OR Oman*[tw] OR Panama*[tw] OR Poland[tw] OR Pole*[tw] OR Polish[tw] OR Portugal[tw] OR Portuguese[tw] OR Puerto Rico*[tw] OR Qatar*[tw] OR Romania*[tw] OR Scot*[tw] OR Scotland[tw] OR Seychelle*[tw] OR Singapore*[tw] OR Slovakia*[tw] OR Slovenia*[tw] OR Spain[tw] OR Spaniard*[tw] OR Spanish[tw] OR Sweden[tw] OR Swedish[tw] OR Swiss[tw] OR Switzerland[tw] OR Taiwan*[tw] OR Thai*[tw] OR Uruguay*[tw] | 5,394,188 |
| **UK-Filter** | 34 | "United Kingdom"[Mesh] | 390,209 |
|  | 35 | ("national health service*"[tiab] OR nhs[tiab] OR "national health service*"[ad] OR nhs[ad]) | 270,709 |
|  | 36 | (english[tiab] NOT ("published english"[tiab:~5] OR "publication english"[tiab:~5] OR "translate english"[tiab:~5] OR "translated english"[tiab:~5] OR "written english"[tiab:~5] OR "language english"[tiab:~5] OR "speak english"[tiab:~5] OR "literature english"[tiab:~5] OR "citation english"[tiab:~5])) | 64,080 |
|  | 37 | (gb[tiab] OR "g.b."[tiab] OR britain*[tiab] OR (british*[tiab] NOT "british columbia"[tiab]) OR uk[tiab] OR "u.k."[tiab] OR united kingdom*[tiab] OR (england*[tiab] NOT "new england"[tiab:~0]) OR northern ireland*[tiab] OR northern irish*[tiab] OR scotland*[tiab] OR scottish*[tiab] OR ((wales[tiab] OR "south wales"[tiab:~0]) NOT "new south wales"[tiab:~0]) OR welsh*[tiab]) | 326,654 |
|  | 38 | (bath[tiab] OR "bath's"[tiab] OR ((birmingham[tiab] NOT alabama*[tiab]) OR ("birmingham's"[tiab] NOT alabama*[tiab]) OR bradford[tiab] OR "bradford's"[tiab] OR brighton[tiab] OR "brighton's"[tiab] OR bristol[tiab] OR "bristol's"[tiab] OR carlisle*[tiab] OR "carlisle's"[tiab] OR (cambridge[tiab] NOT (massachusetts*[tiab] OR boston*[tiab] OR harvard*[tiab])) OR ("cambridge's"[tiab] NOT (massachusetts*[tiab] OR boston*[tiab] OR harvard*[tiab])) OR (canterbury[tiab] NOT zealand*[tiab]) OR ("canterbury's"[tiab] NOT zealand*[tiab]) OR chelmsford[tiab] OR "chelmsford's"[tiab] OR chester[tiab] OR "chester's"[tiab] OR chichester[tiab] OR "chichester's"[tiab] OR coventry[tiab] OR "coventry's"[tiab] OR derby[tiab] OR "derby's"[tiab] OR (durham[tiab] NOT (carolina*[tiab] OR nc[tiab])) OR ("durham's"[tiab] NOT (carolina*[tiab] OR nc[tiab])) OR ely[tiab] OR "ely's"[tiab] OR exeter[tiab] OR "exeter's"[tiab] OR gloucester[tiab] OR "gloucester's"[tiab] OR hereford[tiab] OR "hereford's"[tiab] OR hull[tiab] OR "hull's"[tiab] OR lancaster[tiab] OR "lancaster's"[tiab] OR leeds*[tiab] OR leicester[tiab] OR "leicester's"[tiab] OR (lincoln[tiab] NOT nebraska*[tiab]) OR ("lincoln's"[tiab] NOT nebraska*[tiab]) OR (liverpool[tiab] NOT (new south wales*[tiab] OR nsw[tiab])) OR ("liverpool's"[tiab] NOT (new south wales*[tiab] OR nsw[tiab])) OR ((london[tiab] NOT (ontario*[tiab] OR ont[tiab] OR toronto*[tiab])) OR ("london's"[tiab] NOT (ontario*[tiab] OR ont[tiab] OR toronto*[tiab])) OR manchester[tiab] OR "manchester's"[tiab] OR (newcastle[tiab] NOT (new south wales*[tiab] OR nsw[tiab])) OR ("newcastle's"[tiab] NOT (new south wales*[tiab] OR nsw[tiab])) OR norwich[tiab] OR "norwich's"[tiab] OR nottingham[tiab] OR "nottingham's"[tiab] OR oxford[tiab] OR "oxford's"[tiab] OR peterborough[tiab] OR "peterborough's"[tiab] OR plymouth[tiab] OR "plymouth's"[tiab] OR portsmouth[tiab] OR "portsmouth's"[tiab] OR preston[tiab] OR "preston's"[tiab] OR ripon[tiab] OR "ripon's"[tiab] OR salford[tiab] OR "salford's"[tiab] OR salisbury[tiab] OR "salisbury's"[tiab] OR sheffield[tiab] OR "sheffield's"[tiab] OR southampton[tiab] OR "southampton's"[tiab] OR st albans[tiab] OR stoke[tiab] OR "stoke's"[tiab] OR sunderland[tiab] OR "sunderland's"[tiab] OR truro[tiab] OR "truro's"[tiab] OR wakefield[tiab] OR "wakefield's"[tiab] OR wells[tiab] OR westminster[tiab] OR "westminster's"[tiab] OR winchester[tiab] OR "winchester's"[tiab] OR wolverhampton[tiab] OR "wolverhampton's"[tiab] OR (worcester[tiab] NOT (massachusetts*[tiab] OR boston*[tiab] OR harvard*[tiab])) OR ("worcester's"[tiab] NOT (massachusetts*[tiab] OR boston*[tiab] OR harvard*[tiab])) OR (york[tiab] NOT ("new york*"[tiab] OR ny[tiab] OR ontario*[tiab] OR ont[tiab] OR toronto*[tiab])) OR ("york's"[tiab] NOT ("new york*"[tiab] OR ny[tiab] OR ontario*[tiab] OR ont[tiab] OR toronto*[tiab]))))) | 195,029 |
|  | 39 | (bangor[tiab] OR "bangor's"[tiab] OR cardiff[tiab] OR "cardiff's"[tiab] OR newport[tiab] OR "newport's"[tiab] OR "st asaph"[tiab] OR "st asaph's"[tiab] OR st davids[tiab] OR swansea[tiab] OR "swansea's"[tiab]) | 3,417 |
|  | 40 | (aberdeen[tiab] OR "aberdeen's"[tiab] OR dundee[tiab] OR "dundee's"[tiab] OR edinburgh[tiab] OR "edinburgh's"[tiab] OR glasgow[tiab] OR "glasgow's"[tiab] OR inverness[tiab] OR (perth[tiab] NOT australia*[tiab]) OR ("perth's"[tiab] NOT australia*[tiab]) OR stirling[tiab] OR "stirling's"[tiab]) | 41,936 |
|  | 41 | (armagh[tiab] OR "armagh's"[tiab] OR belfast[tiab] OR "belfast's"[tiab] OR lisburn[tiab] OR "lisburn's"[tiab] OR londonderry[tiab] OR "londonderry's"[tiab] OR derry[tiab] OR "derry's"[tiab] OR newry[tiab] OR "newry's"[tiab]) | 1,559 |
|  | 42 | #34 OR #35 OR #36 OR #37 OR #38 OR #39 OR #40 OR #41 | 991,125 |
| **Canada-Filter** | 43 | Canada[Mesh] OR Canada[tw] OR Canad*[tw] | 253,141 |
|  | 44 | ("British Columbia"[tiab] OR "Colombie Britannique"[tiab] OR Alberta*[tiab] OR Saskatchewan[tiab] OR Manitoba*[tiab] OR Ontario[tiab] OR Quebec[tiab] OR "Nouveau Brunswick"[tiab] OR "New Brunswick"[tiab] OR "Nova Scotia"[tiab] OR "Nouvelle Ecosse"[tiab] OR "Prince Edward Island"[tiab] OR Newfoundland[tiab] OR Labrador[tiab] OR Nunavut[tiab] OR NWT[tiab] OR "Northwest Territories"[tiab] OR Yukon[tiab] OR Nunavik[tiab] OR Inuvialuit[tiab]) | 86,049 |
|  | 45 | Abbotsford[tiab] OR "Ajax"[tiab] OR "Aurora"[tiab] OR "Barrie"[tiab] OR "Belleville"[tiab] OR "Blainville"[tiab] OR "Brampton"[tiab] OR "Brantford"[tiab] OR "Brossard"[tiab] OR "Burlington"[tiab] OR "Burnaby"[tiab] OR "Caledon"[tiab] OR "Calgary"[tiab] OR "cape breton"[tiab] OR "chatham kent"[tiab] OR "Chilliwack"[tiab] OR "Clarington"[tiab] OR "Coquitlam"[tiab] OR "Drummondville"[tiab] OR "Edmonton"[tiab] OR "Fredericton"[tiab] OR "fort mcmurray"[tiab] OR "Gatineau"[tiab] OR "Granby"[tiab] OR "grande prairie"[tiab] OR "Guelph"[tiab] OR ("Halton Hills"[tiab]) OR "Iqaluit"[tiab] OR "Inuvik"[tiab] OR "Kamloops"[tiab] OR "kawartha lakes"[tiab] OR "Kelowna"[tiab] OR "Kingston"[tiab] OR "Kitchener"[tiab] OR "Langley"[tiab] OR "Laval"[tiab] OR "Lethbridge"[tiab] OR "Levis"[tiab] OR "Longueuil"[tiab] OR "maple ridge"[tiab] OR "Markham"[tiab] OR "medicine hat"[tiab] OR "Milton"[tiab] OR "Mirabel"[tiab] OR "Mississauga"[tiab] OR "Moncton"[tiab] OR "Montreal"[tiab] OR "Nanaimo"[tiab] OR "new westminster"[tiab] OR "Newmarket"[tiab] OR "niagara falls"[tiab] OR "norfolk county"[tiab] OR "north bay"[tiab] OR "north vancouver"[tiab] OR "north vancouver"[tiab] OR "Oakville"[tiab] OR "Oshawa"[tiab] OR "Ottawa"[tiab] OR "port coquitlam"[tiab] OR "prince george"[tiab] OR "quebec city"[tiab] OR "red deer"[tiab] OR "Regina"[tiab] OR "Repentigny"[tiab] OR "Saanich"[tiab] OR "Saguenay"[tiab] OR "saint john"[tiab] OR "Saint-Hyacinthe"[tiab] OR "Saint-Jean-sur-Richelieu"[tiab] OR "Saint-Jerome"[tiab] OR "Sarnia"[tiab] OR "Saskatoon"[tiab] OR "sault ste marie"[tiab] OR "Sherbrooke"[tiab] OR "st albert"[tiab] OR "st catharines"[tiab] OR "st john s"[tiab] OR ("Strathcona County"[tiab:~2]) OR "Terrebonne"[tiab] OR "thunder bay"[tiab] OR "Toronto"[tiab] OR "Trois-Rivieres"[tiab] OR "Vancouver"[tiab] OR "Vaughan"[tiab] OR (("Airdrie"[tiab] OR "Cambridge"[tiab] OR ("Halifax"[tiab] OR "Hamilton"[tiab] OR "London"[tiab] OR "Peterborough"[tiab] OR "Pickering"[tiab] OR "Richmond"[tiab] OR "richmond hill"[tiab] OR "Sudbury"[tiab] OR "Surrey"[tiab] OR "Victoria"[tiab] OR "Waterloo"[tiab] OR "Welland"[tiab] OR "Whitby"[tiab] OR "Windsor"[tiab])) NOT ("UK"[tw] OR "Britain"[tw] OR "united kingdom"[tw] OR "England"[tw] OR "Australia"[tw])) OR "Whitehorse"[tiab] OR "Winnipeg"[tiab] OR "wood buffalo"[tiab] OR "Yellowknife"[tiab] | 136,555 |
|  | 46 | #43 OR #44 OR #45 | 382,287 |
| **Spain-Filter** | 47 | ((spain OR espagne OR espana OR spagna) OR (spain[ad] OR espagne[ad] OR espana[ad] OR spanien[ad] OR spagna[ad]) OR (catalunya[ad] OR catalonia[ad] OR catalogne[ad] OR cataluna[ad] OR catala[ad] OR barcelon*[ad] OR tarragona[ad] OR lleida[ad] OR lerida[ad] OR girona[ad] OR gerona[ad] OR sabadell[ad] OR hospitalet[ad] OR l’hospitalet[ad]) OR (valencia*[ad] OR castello*[ad] OR alacant[ad] OR alicant*[ad]) OR (murcia*[ad] OR (cartagen*[ad] NOT indias[ad])) OR (andalu*[ad] OR sevill*[ad] OR granad*[ad] OR huelva[ad] OR almeria[ad] OR cadiz[ad] OR jaen[ad] OR malaga[ad] OR (cordoba[ad] NOT argentin*[ad])) OR (extremadura[ad] OR caceres[ad] OR badajoz[ad] OR madrid[ad]) OR (castilla[ad] OR salamanca[ad] OR zamora[ad] OR valladolid[ad] OR segovia[ad] OR soria[ad] OR palencia[ad] OR avila[ad] OR burgos[ad]) OR (leon[ad] NOT (france[ad] OR clermont[ad] OR rennes[ad] OR lyon[ad] OR USA[ad] OR mexic*[ad])) OR (galicia[ad] OR gallego[ad] OR compostela[ad] OR vigo[ad] OR corun*[ad] OR ferrol[ad] OR orense[ad] OR ourense[ad] OR pontevedra[ad] OR lugo[ad]) OR (oviedo[ad] OR gijon[ad] OR asturia*[ad]) OR (cantabr*[ad] OR santander[ad]) OR (vasco[ad] OR euskadi[ad] OR basque[ad] OR bilbao[ad] OR bilbo[ad] OR donosti*[ad] OR san sebastian[ad] OR vizcaya[ad] OR bizkaia[ad] OR guipuzcoa[ad] OR gipuzkoa[ad] OR alava[ad] OR araba[ad] OR vitoria[ad] OR gasteiz[AD]) OR (navarr*[ad] OR nafarroa[ad] OR pamplona[ad] OR iruna[ad] OR irunea[ad]) OR (logron*[ad] OR rioj*[ad]) OR (aragon*[ad] OR zaragoza[ad] OR teruel[ad] OR huesca[ad]) OR (mancha[ad] OR ciudad real[ad] OR albacete[ad] OR cuenca[ad]) OR (toledo[ad] NOT (ohio[ad] OR us[ad] OR usa[ad] OR OH[ad])) OR (guadalajara[ad] NOT mexic*[ad]) OR (balear*[ad] OR mallorca[ad] OR menorca[ad] OR ibiza[ad] OR eivissa[ad]) OR (palmas[ad] OR lanzarote[ad] OR canari*[ad] OR tenerif*[ad]) OR (ceuta[ad] OR melilla[ad])) OR (osasunbide*[ad] OR osakidetza[ad] OR insalud[ad] OR sergas[ad] OR catsalut[ad] OR sespa[ad] OR osasunbidea[ad] OR imsalud[ad] OR sescam[ad] OR ib-salut[ad]) | 803,004 |
| **Restrict to World Bank 'High-Income' Countries** | 48 | #32 AND (#33 OR #42 OR #46 OR #47) | 490 |
| **Restrict to Non-US /  Non-African countries** | 49 | #48 NOT ((africa[mesh] OR north america[mesh:noexp] OR united states[mesh]) NOT (asia[mesh] OR australia[mesh] OR canada[mesh] OR europe[mesh] OR south america[mesh])) | **460** |

# **Supplementary Table 2c. Search Terms for Prevalence of PrEP Use Sources (Cochrane Library)**

| **#** | **Search Terms** | **Number of Hits  (July 2023)** |
| --- | --- | --- |
| 1 | MeSH descriptor: [Pre-Exposure Prophylaxis] explode all trees | 395 |
| 2 | MeSH descriptor: [Chemoprevention] explode all trees | 2419 |
| 3 | pre‐exposure NEAR/2 prophylaxis OR preexposure NEAR/2 prophylaxis OR PREP OR anti‐retroviral NEAR/2 chemoprophylaxis OR antiretroviral NEAR/2 chemoprophylaxis OR chemoprevention OR HIV NEAR/2 prophylaxis | 4504 |
| 4 | MeSH descriptor: [Emtricitabine, Tenofovir Disoproxil Fumarate Drug Combination] explode all trees | 207 |
| 5 | 'emtricitabine tenofovir alafenamide' | 463 |
| 6 | fixed dose NEAR/1 emtricitabine tenofovir OR 'cabotegravir' NEAR/1 'extended-release' OR Truvada OR Descovy OR Apretude | 272 |
| 7 | #1 OR #2 OR #3 OR #4 OR #5 OR #6 | 7080 |
| 8 | MeSH descriptor: [HIV Infections] explode all trees and with qualifier(s): [prevention & control - PC] | 3149 |
| 9 | HIV NEAR/5 prevent OR HIV NEAR/5 prevents OR HIV NEAR/5 prevention OR human immunodeficiency virus NEAR/5 prevent OR human immunodeficiency virus NEAR/5 prevents OR human immunodeficiency virus NEAR/5 prevention | 4874 |
| 10 | #8 OR #9 | 6353 |
| 11 | MeSH descriptor: [Prevalence] explode all trees | 8698 |
| 12 | MeSH descriptor: [Incidence] explode all trees | 15075 |
| 13 | prevalence* OR prevalent OR incidence* OR incident OR demographic* OR epidemiolog* OR estimate* OR frequency OR percent* OR rate OR rates OR uptake | 801607 |
| 14 | #11 OR #12 OR #13 | 801607 |
| 15 | MeSH descriptor: [Sex Workers] explode all trees | 119 |
| 16 | MeSH descriptor: [Sex Work] explode all trees | 132 |
| 17 | Sex NEAR/1 work OR sex NEXT work OR sex NEXT work* OR prostitute* OR prostitution OR call NEXT girl* OR exchange NEAR/1 sex OR ladies NEAR/1 night OR sex NEAR/1 payment OR survival NEAR/1 sex OR transactional NEAR/1 sex OR walk NEAR/1 pavement OR nightwalker OR prostitut* OR sex NEXT industr* OR streetwalker* OR whore* | 909 |
| 18 | #15 OR #16 OR #17 | 909 |
| 19 | MeSH descriptor: [Homosexuality, Male] explode all trees | 668 |
| 20 | MeSH descriptor: [Bisexuality] explode all trees | 86 |
| 21 | MeSH descriptor: [Sexual and Gender Minorities] explode all trees | 421 |
| 22 | Bisex* OR bisexuality OR bisexual OR Bi-sex* OR bi-sexuality OR bi-sexual OR Men NEAR/2 who have sex with men OR men NEAR/2 have sex with men OR men NEAR/2 having sex with men OR bisexual* OR homosexual* OR MSM OR 'male to male sexual contact' OR men NEAR/1 sexual relations with men OR men NEAR/1 who have sex with both men and women OR 'non binary' OR non NEXT heterosexual* OR bisexual* OR cruisin OR cruising* OR gay OR gays OR GBMSM OR GB-MSM OR homosexual* OR lesbigay* OR lgbt OR MASM OR MSM OR MSMW OR queer* | 101729 |
| 23 | #19 OR #20 OR #21 OR #22 | 101784 |
| 24 | MeSH descriptor: [Transgender Persons] explode all trees | 109 |
| 25 | MeSH descriptor: [Transsexualism] explode all trees | 40 |
| 26 | trans NEXT female* OR trans NEXT male* OR trans NEAR/1 men OR trans NEAR/1 people OR trans NEAR/1 peoples OR trans NEAR/1 person OR trans NEXT wom* OR transwom* OR transman OR transmen OR transpeople OR transperson* OR gender NEAR/1 reassignment OR gender NEAR/1 variant OR intersex NEXT individual* OR intersex NEAR/1 people OR intersex NEAR/1 person OR sexual NEAR/1 dissident OR trans NEXT sexual* OR two NEAR/1 spirit OR glbt OR glbtq OR lgbt OR lgbtq OR lgbtqq OR transgender* OR trans NEAR/1 man | 759 |
| 27 | #24 OR #25 OR #26 | 777 |
| 28 | MeSH descriptor: [Drug Users] explode all trees | 149 |
| 29 | MeSH descriptor: [Substance Abuse, Intravenous] explode all trees | 491 |
| 30 | I V NEAR/3 drug user OR I V NEAR/3 drug users OR injectable NEAR/3 drug user OR injectable NEAR/3 drug users OR intravenous NEAR/3 drug user OR intra-venous NEAR/3 drug user OR intravenous NEAR/3 drug users OR intra-venous NEAR/3 drug users OR IV NEAR/3 drug user OR IV NEAR/3 drug users OR IVDU OR IVDUs OR IVDUs OR people NEAR/3 inject drugs OR people NEAR/3 injected drugs OR people NEAR/3 injecting drugs OR people NEAR/3 who use drugs OR person NEAR/3 inject drugs OR person NEAR/3 who use drugs OR persons NEAR/3 injecting drugs OR persons NEAR/3 who use drugs OR PWID OR PWIDs OR PWIDs OR PWUD OR PWUDs OR PWUDs OR injecting NEAR/1 drug user OR intravenous NEAR/1 drug abuse OR intravenous NEAR/1 drug use OR intravenous NEAR/1 substance abuse OR intravenous NEAR/1 substance misuse OR intravenous NEAR/1 substance use OR parenteral NEAR/1 drug abuse OR parenteral NEAR/1 drug use OR people NEAR/1 who inject drugs OR person NEAR/1 who inject drugs OR persons NEAR/1 who inject drugs OR injecting NEAR/1 drug users OR intravenous NEAR/1 drug abuser OR intravenous NEAR/1 drug user OR intravenous NEAR/1 substance abuser OR intravenous NEAR/1 substance misuser OR intravenous NEAR/1 substance user OR parenteral NEAR/1 drug abuser OR parenteral NEAR/1 drug user OR intravenous NEAR/1 drug abusers OR intravenous NEAR/1 drug users OR intravenous NEAR/1 substance abusers OR intravenous NEAR/1 substance misusers OR intravenous NEAR/1 substance users OR parenteral NEAR/1 drug abusers OR parenteral NEAR/1 drug users OR ((inject*:ti OR intraven*:ti OR parenteral:ti OR use:ti OR misus*:ti OR user:ti OR users:ti) AND (drug*:ti OR substance*:ti)) | 49939 |
| 31 | #28 OR #29 OR #30 | 49984 |
| 32 | MeSH descriptor: [Correctional Facilities] explode all trees | 189 |
| 33 | MeSH descriptor: [Jails] explode all trees | 7 |
| 34 | MeSH descriptor: [Prisons] explode all trees | 182 |
| 35 | MeSH descriptor: [Prisoners] explode all trees | 421 |
| 36 | correctional NEAR/1 facilities OR correctional NEAR/1 facility OR 'correctional institution' OR 'correctional institutions' OR gaol OR gaols OR penal NEAR/1 facilities OR penal NEAR/1 facility OR penal NEAR/1 institution OR penal NEAR/1 institutions OR custodial OR imprison* OR incarcerat* OR inmate* OR jail OR jails OR penitentiar* OR prison* OR closed NEXT setting* OR cellmate* OR confinement* OR detainee* OR detention* OR offender OR offenders OR penal OR penitentiar* OR reformator* OR remand* OR convict* | 4010 |
| 37 | #32 OR #33 OR #34 OR #35 OR #36 | 4012 |
| 38 | serodiscordan* OR sero-discordan* OR sero NEXT discordan* OR mixed NEAR/2 infection status | 900 |
| 39 | #18 OR #23 OR #27 OR #31 OR #37 OR #38 | 149532 |
| 40 | #7 AND #10 AND #14 AND #39 | 442 |
| 41 | #40 with Publication Year from 2017 to 2023, in Trials | 302 |
| 42 | MeSH descriptor: [Randomized Controlled Trial] explode all trees | 25732 |
| 43 | MeSH descriptor: [Randomized Controlled Trials as Topic] explode all trees | 47396 |
| 44 | MeSH descriptor: [Controlled Clinical Trial] explode all trees | 38477 |
| 45 | MeSH descriptor: [Controlled Clinical Trials as Topic] explode all trees | 47802 |
| 46 | MeSH descriptor: [Review] explode all trees | 1002 |
| 47 | MeSH descriptor: [Review Literature as Topic] explode all trees | 435 |
| 48 | #42 OR #43 OR #44 OR #45 OR #46 OR #47 | 86578 |
| 49 | #41 NOT #48 | 283 |
| 50 | ('animal cell' OR 'animal experiment' OR 'animal health' OR 'animal model' OR 'animal tissue' OR 'bird' OR 'cancer model' OR 'canine' OR 'dog' OR 'ex vivo study' OR 'goat' OR 'Hep-G2 cell line' OR 'in vitro study' OR 'knockout mouse' OR 'Kupffer cell' OR 'mammal' OR 'model' OR 'mouse' OR 'mouse model' OR 'murine' OR mice OR 'nonhuman' OR 'nude rat' OR 'rat' OR 'rat model' OR 'tumor cell' OR 'tumor model' OR 'tumor xenograft' OR 'veterinary') | 200247 |
| 51 | #49 NOT #50 | 182 |
| 52 | MeSH descriptor: [Africa] explode all trees | 11427 |
| 53 | MeSH descriptor: [North America] this term only | 715 |
| 54 | MeSH descriptor: [United States] explode all trees | 27438 |
| 55 | MeSH descriptor: [Asia] explode all trees | 33380 |
| 56 | MeSH descriptor: [Australia] explode all trees | 6637 |
| 57 | MeSH descriptor: [Canada] explode all trees | 5612 |
| 58 | MeSH descriptor: [Europe] explode all trees | 41960 |
| 59 | MeSH descriptor: [South America] explode all trees | 3876 |
| 60 | (#52 OR #53 OR #54) NOT (#55 OR #56 Or #57 OR 49 OR #59) | 34386 |
| 61 | #51 NOT #60 | **148** |

# **Supplementary Table 3a. Search Terms for PrEP Effectiveness Sources (Embase)**

| **Term Group** | **#** | **Search Terms** | **Number of Hits  (July 2023)** |
| --- | --- | --- | --- |
| **Pre-Exposure Prophylaxis (PrEP)** | 1 | "Pre-Exposure Prophylaxis"[Mesh] OR "Chemoprevention"[Mesh] | 53,370 |
|  | 2 | "pre‐exposure prophylaxis"[tiab:~2] OR "preexposure prophylaxis"[tiab:~2] OR PREP[tiab] OR "anti‐retroviral chemoprophylaxis"[tiab:~2] OR "antiretroviral chemoprophylaxis"[tiab:~2] OR "Chemoprevention"[Mesh] OR chemoprevention[tiab] OR "HIV prophylaxis"[tiab:~2] | 46,938 |
|  | 3 | "Emtricitabine, Tenofovir Disoproxil Fumarate Drug Combination"[Mesh] OR "emtricitabine tenofovir alafenamide"[Supplementary Concept] | 6,041 |
|  | 4 | "fixed dose emtricitabine tenofovir"[tiab:~2] OR Truvada[tw] OR Descovy[tw] | 1,879 |
|  | 5 | #1 OR #2 OR #3 OR #4 | 74,369 |
| **HIV Infection Prevention** | 6 | "HIV Infections/prevention and control"[Majr] | 42,456 |
|  | 7 | "HIV prevent"[tiab:~5] OR "HIV prevents"[tiab:~5] OR "HIV prevention"[tiab:~5] OR "human immunodeficiency virus prevent"[tiab:~5] OR "human immunodeficiency virus prevents"[tiab:~5] OR "human immunodeficiency virus prevention"[tiab:~5] | 20,830 |
|  | 8 | (#6 OR #7) | 53,796 |
| **Treatment Outcome** | 9 | "Treatment Outcome"[Mesh] | 3,096,863 |
|  | 10 | effectiv*[tiab] OR efficacious*[tiab] OR efficacy[tiab] OR efficiency[tiab] OR outcome*[tiab] OR response[tiab] OR responsiveness[tiab] | 11,828,722 |
|  | 11 | #9 OR #10 | 11,902,266 |
| **Combination: HIV and PrEP** | 12 | #5 AND #8 AND #11 | 3,910 |
| **Restrict to Publication On/After  1st Jan 2017** | 13 | #12 AND (2017/1/1:3000/12/12[pdat]) | 2,482 |
| **Remove Irrelevant Publication Types - Expanded** | 14 | #13 NOT ("Review"[Publication Type] OR "Systematic Review"[Publication Type] OR "Meta-Analysis"[Publication Type] OR "Case Reports" [Publication Type] OR "Published Erratum"[Publication Type] OR "comment"[Publication Type] OR "Editorial"[Publication Type] OR "Controlled Clinical Trial"[Publication Type] OR "Randomized Controlled Trial"[Publication Type] OR "Clinical Trial, Phase III" [Publication Type]) | 1,249 |
| **Animal Studies** | 15 | #14 NOT ("animal cell"[tiab] OR "animal experiment"[tiab] OR "animal health"[tiab] OR "animal model"[tiab] OR "animal tissue"[tiab] OR "bird"[tiab] OR "cancer model"[tiab] OR "canine"[tiab] OR "dog"[tiab] OR "ex vivo study"[tiab] OR "goat"[tiab] OR "Hep-G2 cell line"[tiab] OR "in vitro study"[tiab] OR "knockout mouse"[tiab] OR "Kupffer cell"[tiab] OR "mammal"[tiab] OR "model"[tiab] OR "mouse"[tiab] OR "mouse model"[tiab] OR "murine"[tiab] OR mice[tiab] OR "nonhuman"[tiab] OR "nude rat"[tiab] OR "rat"[tiab] OR "rat model"[tiab] OR "tumor cell"[tiab] OR "tumor model"[tiab] OR "tumor xenograft"[tiab] OR "veterinary"[tiab]) | 1,055 |
| **World Bank 'High-Income' Countries** | 16 | "Andorra"[Mesh] OR "Antigua and Barbuda"[Mesh] OR "Antigua Barbuda"[tiab:~1] OR "Aruba"[Mesh] OR "Asia"[Mesh] OR "Australia"[Mesh] OR "Austria"[Mesh] OR "Bahamas"[Mesh] OR "Bahrain"[Mesh] OR "Barbados"[Mesh] OR "Belgium"[Mesh] OR "Bermuda"[Mesh] OR "British Virgin Islands"[Mesh] OR "Brunei"[Mesh] OR "Canada"[Mesh] OR "Cayman Island*"[tw] OR "Channel Islands"[Mesh] OR "Chile"[Mesh] OR "Cook Island*"[tw] OR "Croatia"[Mesh] OR "Curacao"[Mesh] OR "Cyprus"[Mesh] OR "Czech Republic"[Mesh] OR "Czech Republic*"[tw] OR "Denmark"[Mesh] OR "England"[Mesh] OR "Estonia"[Mesh] OR "Europe"[Mesh] OR "Faroe Island*"[tw] OR "Finland"[Mesh] OR "France"[Mesh] OR "French Polynesia*"[tw] OR "Germany"[Mesh] OR "Gibraltar"[Mesh] OR "Great Britain"[tiab:~1] OR "Great Britain"[tiab:~1] OR "Greece"[Mesh] OR "Greenland"[Mesh] OR "Guam"[Mesh] OR "Guernsey"[Mesh] OR "Hong Kong*"[tw] OR "Hungary"[Mesh] OR "Iceland"[Mesh] OR "Ireland"[Mesh] OR "Isle of Man"[tw] OR "Israel"[Mesh] OR "Italy"[Mesh] OR "Japan"[Mesh] OR "Jersey Channel Island*"[tw] OR "Kuwait"[Mesh] OR "Latvia"[Mesh] OR "Liechtenstein"[Mesh] OR "Lithuania"[Mesh] OR "Luxembourg"[Mesh] OR "Macau"[Mesh] OR "Malta"[Mesh] OR "Monaco"[Mesh] OR "Netherlands"[Mesh] OR "New Caledonia"[Mesh] OR "New Zealand"[Mesh] OR "New Zealand*"[tw] OR "Northern Mariana Island*"[tw] OR "Norway"[Mesh] OR "Oman"[Mesh] OR "Panama"[Mesh] OR "Poland"[Mesh] OR "Polynesia"[Mesh] OR "Portugal"[Mesh] OR "Puerto Rico"[Mesh] OR "Qatar"[Mesh] OR "Republic of Korea"[Mesh] OR "Romania"[Mesh] OR "Saint Kitts and Nevis"[Mesh] OR "Saint Kitts Nevis"[tiab:~1] OR "Saint Maarten*"[tw] OR "Saint Martin*"[tw] OR "San Marino"[Mesh] OR "San Marino*"[tw] OR "Saudi Arabia"[Mesh] OR "Saudi Arabia*"[tw] OR "Scotland"[Mesh] OR "Seychelles"[Mesh] OR "Singapore"[Mesh] OR "Slovakia"[Mesh] OR "Slovenia"[Mesh] OR "Spain"[Mesh] OR "Sweden"[Mesh] OR "Switzerland"[Mesh] OR "Taiwan"[Mesh] OR "Thailand"[Mesh] OR "Trinidad and Tobago"[Mesh] OR "Trinidad Tobago"[tiab:~1] OR "Turks Caicos"[tiab:~1] OR "U K"[tw] OR "UK"[tw] OR "United Arab Emirates"[Mesh] OR "United Arab Emirates"[tw] OR "United Kingdom"[Mesh] OR "United Kingdom"[tw] OR "United States Virgin Islands"[Mesh] OR "Uruguay"[Mesh] OR "Virgin Island*"[tw] OR Andorra*[tw] OR Aruba*[tw] OR Australia*[tw] OR Austria*[tw] OR Bahama*[tw] OR Bahrain*[tw] OR Barbados*[tw] OR Belgian*[tw] OR Belgium[tw] OR Bermuda*[tw] OR Britain[tw] OR British[tw] OR Brunei*[tw] OR Canada[tw] OR Canadian*[tw] OR Chile*[tw] OR Croatia*[tw] OR Curaçao*[tw] OR Cyprus*[tw] OR Dane*[tw] OR Danish[tw] OR Denmark[tw] OR Dutch*[tw] OR England[tw] OR English[tw] OR Estonia*[tw] OR Finland[tw] OR Finn*[tw] OR France[tw] OR French*[tw] OR German*[tw] OR Gibraltar*[tw] OR Greece*[tw] OR Greenland*[tw] OR Guam*[tw] OR Guernsey*[tw] OR Hungary*[tw] OR Iceland*[tw] OR Ireland[tw] OR Irish*[tw] OR Israel*[tw] OR Italian*[tw] OR Italy[tw] OR Japan*[tw] OR Korea*[tw] OR Kuwait*[tw] OR Latvia*[tw] OR Liechtenstein*[tw] OR Lithuania*[tw] OR Luxembourg*[tw] OR Macao*[tw] OR Malta*[tw] OR Monaco*[tw] OR Nauru*[tw] OR Netherlands[tw] OR New Caledonia*[tw] OR Norway[tw] OR Norwegian*[tw] OR Oman*[tw] OR Panama*[tw] OR Poland[tw] OR Pole*[tw] OR Polish[tw] OR Portugal[tw] OR Portuguese[tw] OR Puerto Rico*[tw] OR Qatar*[tw] OR Romania*[tw] OR Scot*[tw] OR Scotland[tw] OR Seychelle*[tw] OR Singapore*[tw] OR Slovakia*[tw] OR Slovenia*[tw] OR Spain[tw] OR Spaniard*[tw] OR Spanish[tw] OR Sweden[tw] OR Swedish[tw] OR Swiss[tw] OR Switzerland[tw] OR Taiwan*[tw] OR Thai*[tw] OR Uruguay*[tw] | 6,030,636 |
| **UK-Filter** | 17 | "United Kingdom"[Mesh] | 484,634 |
|  | 18 | ("national health service*"[tiab] OR nhs[tiab] OR "national health service*"[ad] OR nhs[ad]) | 526,717 |
|  | 19 | (english[tiab] NOT ("published english"[tiab:~5] OR "publication english"[tiab:~5] OR "translate english"[tiab:~5] OR "translated english"[tiab:~5] OR "written english"[tiab:~5] OR "language english"[tiab:~5] OR "speak english"[tiab:~5] OR "literature english"[tiab:~5] OR "citation english"[tiab:~5])) | 43,464,662 |
|  | 20 | (gb[tiab] OR "g.b."[tiab] OR britain*[tiab] OR (british*[tiab] NOT "british columbia"[tiab]) OR uk[tiab] OR "u.k."[tiab] OR united kingdom*[tiab] OR (england*[tiab] NOT "new england"[tiab:~0]) OR northern ireland*[tiab] OR northern irish*[tiab] OR scotland*[tiab] OR scottish*[tiab] OR ((wales[tiab] OR "south wales"[tiab:~0]) NOT "new south wales"[tiab:~0]) OR welsh*[tiab]) | 222,768 |
|  | 21 | (bath[tiab] OR "bath's"[tiab] OR ((birmingham[tiab] NOT alabama*[tiab]) OR ("birmingham's"[tiab] NOT alabama*[tiab]) OR bradford[tiab] OR "bradford's"[tiab] OR brighton[tiab] OR "brighton's"[tiab] OR bristol[tiab] OR "bristol's"[tiab] OR carlisle*[tiab] OR "carlisle's"[tiab] OR (cambridge[tiab] NOT (massachusetts*[tiab] OR boston*[tiab] OR harvard*[tiab])) OR ("cambridge's"[tiab] NOT (massachusetts*[tiab] OR boston*[tiab] OR harvard*[tiab])) OR (canterbury[tiab] NOT zealand*[tiab]) OR ("canterbury's"[tiab] NOT zealand*[tiab]) OR chelmsford[tiab] OR "chelmsford's"[tiab] OR chester[tiab] OR "chester's"[tiab] OR chichester[tiab] OR "chichester's"[tiab] OR coventry[tiab] OR "coventry's"[tiab] OR derby[tiab] OR "derby's"[tiab] OR (durham[tiab] NOT (carolina*[tiab] OR nc[tiab])) OR ("durham's"[tiab] NOT (carolina*[tiab] OR nc[tiab])) OR ely[tiab] OR "ely's"[tiab] OR exeter[tiab] OR "exeter's"[tiab] OR gloucester[tiab] OR "gloucester's"[tiab] OR hereford[tiab] OR "hereford's"[tiab] OR hull[tiab] OR "hull's"[tiab] OR lancaster[tiab] OR "lancaster's"[tiab] OR leeds*[tiab] OR leicester[tiab] OR "leicester's"[tiab] OR (lincoln[tiab] NOT nebraska*[tiab]) OR ("lincoln's"[tiab] NOT nebraska*[tiab]) OR (liverpool[tiab] NOT (new south wales*[tiab] OR nsw[tiab])) OR ("liverpool's"[tiab] NOT (new south wales*[tiab] OR nsw[tiab])) OR ((london[tiab] NOT (ontario*[tiab] OR ont[tiab] OR toronto*[tiab])) OR ("london's"[tiab] NOT (ontario*[tiab] OR ont[tiab] OR toronto*[tiab])) OR manchester[tiab] OR "manchester's"[tiab] OR (newcastle[tiab] NOT (new south wales*[tiab] OR nsw[tiab])) OR ("newcastle's"[tiab] NOT (new south wales*[tiab] OR nsw[tiab])) OR norwich[tiab] OR "norwich's"[tiab] OR nottingham[tiab] OR "nottingham's"[tiab] OR oxford[tiab] OR "oxford's"[tiab] OR peterborough[tiab] OR "peterborough's"[tiab] OR plymouth[tiab] OR "plymouth's"[tiab] OR portsmouth[tiab] OR "portsmouth's"[tiab] OR preston[tiab] OR "preston's"[tiab] OR ripon[tiab] OR "ripon's"[tiab] OR salford[tiab] OR "salford's"[tiab] OR salisbury[tiab] OR "salisbury's"[tiab] OR sheffield[tiab] OR "sheffield's"[tiab] OR southampton[tiab] OR "southampton's"[tiab] OR st albans[tiab] OR stoke[tiab] OR "stoke's"[tiab] OR sunderland[tiab] OR "sunderland's"[tiab] OR truro[tiab] OR "truro's"[tiab] OR wakefield[tiab] OR "wakefield's"[tiab] OR wells[tiab] OR westminster[tiab] OR "westminster's"[tiab] OR winchester[tiab] OR "winchester's"[tiab] OR wolverhampton[tiab] OR "wolverhampton's"[tiab] OR (worcester[tiab] NOT (massachusetts*[tiab] OR boston*[tiab] OR harvard*[tiab])) OR ("worcester's"[tiab] NOT (massachusetts*[tiab] OR boston*[tiab] OR harvard*[tiab])) OR (york[tiab] NOT ("new york*"[tiab] OR ny[tiab] OR ontario*[tiab] OR ont[tiab] OR toronto*[tiab])) OR ("york's"[tiab] NOT ("new york*"[tiab] OR ny[tiab] OR ontario*[tiab] OR ont[tiab] OR toronto*[tiab]))))) | 1,860,950 |
|  | 22 | (bangor[tiab] OR "bangor's"[tiab] OR cardiff[tiab] OR "cardiff's"[tiab] OR newport[tiab] OR "newport's"[tiab] OR "st asaph"[tiab] OR "st asaph's"[tiab] OR st davids[tiab] OR swansea[tiab] OR "swansea's"[tiab]) | 22,381 |
|  | 23 | (aberdeen[tiab] OR "aberdeen's"[tiab] OR dundee[tiab] OR "dundee's"[tiab] OR edinburgh[tiab] OR "edinburgh's"[tiab] OR glasgow[tiab] OR "glasgow's"[tiab] OR inverness[tiab] OR (perth[tiab] NOT australia*[tiab]) OR ("perth's"[tiab] NOT australia*[tiab]) OR stirling[tiab] OR "stirling's"[tiab]) | 567,702 |
|  | 24 | (armagh[tiab] OR "armagh's"[tiab] OR belfast[tiab] OR "belfast's"[tiab] OR lisburn[tiab] OR "lisburn's"[tiab] OR londonderry[tiab] OR "londonderry's"[tiab] OR derry[tiab] OR "derry's"[tiab] OR newry[tiab] OR "newry's"[tiab]) | 61,970 |
|  | 25 | #17 OR #18 OR #19 OR #20 OR #21 OR #22 OR #23 OR #24 | 43,465,229 |
| **Canada-Filter** | 26 | Canada[Mesh] OR Canada[tw] OR Canad*[tw] | 1,972,009 |
|  | 27 | ("British Columbia"[tiab] OR "Colombie Britannique"[tiab] OR Alberta*[tiab] OR Saskatchewan[tiab] OR Manitoba*[tiab] OR Ontario[tiab] OR Quebec[tiab] OR "Nouveau Brunswick"[tiab] OR "New Brunswick"[tiab] OR "Nova Scotia"[tiab] OR "Nouvelle Ecosse"[tiab] OR "Prince Edward Island"[tiab] OR Newfoundland[tiab] OR Labrador[tiab] OR Nunavut[tiab] OR NWT[tiab] OR "Northwest Territories"[tiab] OR Yukon[tiab] OR Nunavik[tiab] OR Inuvialuit[tiab]) | 724,265 |
|  | 28 | Abbotsford[tiab] OR "Ajax"[tiab] OR "Aurora"[tiab] OR "Barrie"[tiab] OR "Belleville"[tiab] OR "Blainville"[tiab] OR "Brampton"[tiab] OR "Brantford"[tiab] OR "Brossard"[tiab] OR "Burlington"[tiab] OR "Burnaby"[tiab] OR "Caledon"[tiab] OR "Calgary"[tiab] OR "cape breton"[tiab] OR "chatham kent"[tiab] OR "Chilliwack"[tiab] OR "Clarington"[tiab] OR "Coquitlam"[tiab] OR "Drummondville"[tiab] OR "Edmonton"[tiab] OR "Fredericton"[tiab] OR "fort mcmurray"[tiab] OR "Gatineau"[tiab] OR "Granby"[tiab] OR "grande prairie"[tiab] OR "Guelph"[tiab] OR ("Halton Hills"[tiab]) OR "Iqaluit"[tiab] OR "Inuvik"[tiab] OR "Kamloops"[tiab] OR "kawartha lakes"[tiab] OR "Kelowna"[tiab] OR "Kingston"[tiab] OR "Kitchener"[tiab] OR "Langley"[tiab] OR "Laval"[tiab] OR "Lethbridge"[tiab] OR "Levis"[tiab] OR "Longueuil"[tiab] OR "maple ridge"[tiab] OR "Markham"[tiab] OR "medicine hat"[tiab] OR "Milton"[tiab] OR "Mirabel"[tiab] OR "Mississauga"[tiab] OR "Moncton"[tiab] OR "Montreal"[tiab] OR "Nanaimo"[tiab] OR "new westminster"[tiab] OR "Newmarket"[tiab] OR "niagara falls"[tiab] OR "norfolk county"[tiab] OR "north bay"[tiab] OR "north vancouver"[tiab] OR "north vancouver"[tiab] OR "Oakville"[tiab] OR "Oshawa"[tiab] OR "Ottawa"[tiab] OR "port coquitlam"[tiab] OR "prince george"[tiab] OR "quebec city"[tiab] OR "red deer"[tiab] OR "Regina"[tiab] OR "Repentigny"[tiab] OR "Saanich"[tiab] OR "Saguenay"[tiab] OR "saint john"[tiab] OR "Saint-Hyacinthe"[tiab] OR "Saint-Jean-sur-Richelieu"[tiab] OR "Saint-Jerome"[tiab] OR "Sarnia"[tiab] OR "Saskatoon"[tiab] OR "sault ste marie"[tiab] OR "Sherbrooke"[tiab] OR "st albert"[tiab] OR "st catharines"[tiab] OR "st john s"[tiab] OR ("Strathcona County"[tiab:~2]) OR "Terrebonne"[tiab] OR "thunder bay"[tiab] OR "Toronto"[tiab] OR "Trois-Rivieres"[tiab] OR "Vancouver"[tiab] OR "Vaughan"[tiab] OR (("Airdrie"[tiab] OR "Cambridge"[tiab] OR ("Halifax"[tiab] OR "Hamilton"[tiab] OR "London"[tiab] OR "Peterborough"[tiab] OR "Pickering"[tiab] OR "Richmond"[tiab] OR "richmond hill"[tiab] OR "Sudbury"[tiab] OR "Surrey"[tiab] OR "Victoria"[tiab] OR "Waterloo"[tiab] OR "Welland"[tiab] OR "Whitby"[tiab] OR "Windsor"[tiab])) NOT ("UK"[tw] OR "Britain"[tw] OR "united kingdom"[tw] OR "England"[tw] OR "Australia"[tw])) OR "Whitehorse"[tiab] OR "Winnipeg"[tiab] OR "wood buffalo"[tiab] OR "Yellowknife"[tiab] | 2,502,895 |
|  | 29 | #26 OR #27 OR #28 | 2,988,916 |
| **Spain-Filter** | 30 | ((spain OR espagne OR espana OR spagna) OR (spain[ad] OR espagne[ad] OR espana[ad] OR spanien[ad] OR spagna[ad]) OR (catalunya[ad] OR catalonia[ad] OR catalogne[ad] OR cataluna[ad] OR catala[ad] OR barcelon*[ad] OR tarragona[ad] OR lleida[ad] OR lerida[ad] OR girona[ad] OR gerona[ad] OR sabadell[ad] OR hospitalet[ad] OR l’hospitalet[ad]) OR (valencia*[ad] OR castello*[ad] OR alacant[ad] OR alicant*[ad]) OR (murcia*[ad] OR (cartagen*[ad] NOT indias[ad])) OR (andalu*[ad] OR sevill*[ad] OR granad*[ad] OR huelva[ad] OR almeria[ad] OR cadiz[ad] OR jaen[ad] OR malaga[ad] OR (cordoba[ad] NOT argentin*[ad])) OR (extremadura[ad] OR caceres[ad] OR badajoz[ad] OR madrid[ad]) OR (castilla[ad] OR salamanca[ad] OR zamora[ad] OR valladolid[ad] OR segovia[ad] OR soria[ad] OR palencia[ad] OR avila[ad] OR burgos[ad]) OR (leon[ad] NOT (france[ad] OR clermont[ad] OR rennes[ad] OR lyon[ad] OR USA[ad] OR mexic*[ad])) OR (galicia[ad] OR gallego[ad] OR compostela[ad] OR vigo[ad] OR corun*[ad] OR ferrol[ad] OR orense[ad] OR ourense[ad] OR pontevedra[ad] OR lugo[ad]) OR (oviedo[ad] OR gijon[ad] OR asturia*[ad]) OR (cantabr*[ad] OR santander[ad]) OR (vasco[ad] OR euskadi[ad] OR basque[ad] OR bilbao[ad] OR bilbo[ad] OR donosti*[ad] OR san sebastian[ad] OR vizcaya[ad] OR bizkaia[ad] OR guipuzcoa[ad] OR gipuzkoa[ad] OR alava[ad] OR araba[ad] OR vitoria[ad] OR gasteiz[AD]) OR (navarr*[ad] OR nafarroa[ad] OR pamplona[ad] OR iruna[ad] OR irunea[ad]) OR (logron*[ad] OR rioj*[ad]) OR (aragon*[ad] OR zaragoza[ad] OR teruel[ad] OR huesca[ad]) OR (mancha[ad] OR ciudad real[ad] OR albacete[ad] OR cuenca[ad]) OR (toledo[ad] NOT (ohio[ad] OR us[ad] OR usa[ad] OR OH[ad])) OR (guadalajara[ad] NOT mexic*[ad]) OR (balear*[ad] OR mallorca[ad] OR menorca[ad] OR ibiza[ad] OR eivissa[ad]) OR (palmas[ad] OR lanzarote[ad] OR canari*[ad] OR tenerif*[ad]) OR (ceuta[ad] OR melilla[ad])) OR (osasunbide*[ad] OR osakidetza[ad] OR insalud[ad] OR sergas[ad] OR catsalut[ad] OR sespa[ad] OR osasunbidea[ad] OR imsalud[ad] OR sescam[ad] OR ib-salut[ad]) | 1,655,396 |
| **Restrict to World Bank 'High-Income' Countries** | 31 | #15 AND (#16 OR #25 OR #29 OR #30) | 1,054 |
| **Restrict to Non-US /  Non-African countries** | 32 | #31 NOT ((africa[mesh] OR north america[mesh:noexp] OR united states[mesh]) NOT (asia[mesh] OR australia[mesh] OR canada[mesh] OR europe[mesh] OR south america[mesh])) | **639** |

# **Supplementary Table 3b. Search Terms for PrEP Effectiveness Sources (PubMed)**

| **Term Group** | **#** | **Search Terms** | **Number of Hits  (July 2023)** |
| --- | --- | --- | --- |
| **Pre-Exposure Prophylaxis (PrEP)** | 1 | "Pre-Exposure Prophylaxis"[Mesh] OR "Chemoprevention"[Mesh] | 27,430 |
|  | 2 | "pre‐exposure prophylaxis"[tiab:~2] OR "preexposure prophylaxis"[tiab:~2] OR PREP[tiab] OR "anti‐retroviral chemoprophylaxis"[tiab:~2] OR "antiretroviral chemoprophylaxis"[tiab:~2] OR "Chemoprevention"[Mesh] OR chemoprevention[tiab] OR "HIV prophylaxis"[tiab:~2] | 44,466 |
|  | 3 | "Emtricitabine, Tenofovir Disoproxil Fumarate Drug Combination"[Mesh] OR "emtricitabine tenofovir alafenamide"[Supplementary Concept] OR "cabotegravir" [Supplementary Concept] | 497 |
|  | 4 | "fixed dose emtricitabine tenofovir"[tiab:~1] OR "cabotegravir extended-release"[tiab:~1] OR Truvada[tw] OR Descovy[tw] OR Apretude | 679 |
|  | 5 | #1 OR #2 OR #3 OR #4 | 45,599 |
| **HIV Infection Prevention** | 6 | "HIV Infections/prevention and control"[Majr] | 37,802 |
|  | 7 | "HIV prevent"[tiab:~5] OR "HIV prevents"[tiab:~5] OR "HIV prevention"[tiab:~5] OR "human immunodeficiency virus prevent"[tiab:~5] OR "human immunodeficiency virus prevents"[tiab:~5] OR "human immunodeficiency virus prevention"[tiab:~5] | 32,818 |
|  | 8 | (#6 OR #7) | 56,917 |
| **Treatment Outcome** | 9 | "Treatment Outcome"[Mesh] | 1,245,273 |
|  | 10 | effectiv*[tiab] OR efficacious*[tiab] OR efficacy[tiab] OR efficiency[tiab] OR outcome*[tiab] OR response[tiab] OR responsiveness[tiab] | 7,535,288 |
|  | 11 | #9 OR #10 | 8,019,352 |
| **Combination: HIV and PrEP** | 12 | #5 AND #8 AND #11 | 3,227 |
| **Restrict to Publication On/After  1st Jan 2017** | 13 | #12 AND (2017/1/1:3000/12/12[pdat]) | 2,177 |
| **Remove irrelevant publication types - expanded** | 14 | #13 NOT ("Review"[Publication Type] OR "Systematic Review"[Publication Type] OR "Meta-Analysis"[Publication Type] OR "Case Reports" [Publication Type] OR "Published Erratum"[Publication Type] OR "comment"[Publication Type] OR "Editorial"[Publication Type] OR "Controlled Clinical Trial"[Publication Type] OR "Randomized Controlled Trial"[Publication Type] OR "Clinical Trial, Phase III" [Publication Type]) | 1,661 |
| **Animal Studies** | 15 | #14 NOT ("animal cell"[tiab] OR "animal experiment"[tiab] OR "animal health"[tiab] OR "animal model"[tiab] OR "animal tissue"[tiab] OR "bird"[tiab] OR "cancer model"[tiab] OR "canine"[tiab] OR "dog"[tiab] OR "ex vivo study"[tiab] OR "goat"[tiab] OR "Hep-G2 cell line"[tiab] OR "in vitro study"[tiab] OR "knockout mouse"[tiab] OR "Kupffer cell"[tiab] OR "mammal"[tiab] OR "model"[tiab] OR "mouse"[tiab] OR "mouse model"[tiab] OR "murine"[tiab] OR mice[tiab] OR "nonhuman"[tiab] OR "nude rat"[tiab] OR "rat"[tiab] OR "rat model"[tiab] OR "tumor cell"[tiab] OR "tumor model"[tiab] OR "tumor xenograft"[tiab] OR "veterinary"[tiab]) | 1323 |
| **World Bank 'High-Income' Countries** | 16 | "Andorra"[Mesh] OR "Antigua and Barbuda"[Mesh] OR "Antigua Barbuda"[tiab:~1] OR "Aruba"[Mesh] OR "Asia"[Mesh] OR "Australia"[Mesh] OR "Austria"[Mesh] OR "Bahamas"[Mesh] OR "Bahrain"[Mesh] OR "Barbados"[Mesh] OR "Belgium"[Mesh] OR "Bermuda"[Mesh] OR "British Virgin Islands"[Mesh] OR "Brunei"[Mesh] OR "Canada"[Mesh] OR "Cayman Island*"[tw] OR "Channel Islands"[Mesh] OR "Chile"[Mesh] OR "Cook Island*"[tw] OR "Croatia"[Mesh] OR "Curacao"[Mesh] OR "Cyprus"[Mesh] OR "Czech Republic"[Mesh] OR "Czech Republic*"[tw] OR "Denmark"[Mesh] OR "England"[Mesh] OR "Estonia"[Mesh] OR "Europe"[Mesh] OR "Faroe Island*"[tw] OR "Finland"[Mesh] OR "France"[Mesh] OR "French Polynesia*"[tw] OR "Germany"[Mesh] OR "Gibraltar"[Mesh] OR "Great Britain"[tiab:~1] OR "Great Britain"[tiab:~1] OR "Greece"[Mesh] OR "Greenland"[Mesh] OR "Guam"[Mesh] OR "Guernsey"[Mesh] OR "Hong Kong*"[tw] OR "Hungary"[Mesh] OR "Iceland"[Mesh] OR "Ireland"[Mesh] OR "Isle of Man"[tw] OR "Israel"[Mesh] OR "Italy"[Mesh] OR "Japan"[Mesh] OR "Jersey Channel Island*"[tw] OR "Kuwait"[Mesh] OR "Latvia"[Mesh] OR "Liechtenstein"[Mesh] OR "Lithuania"[Mesh] OR "Luxembourg"[Mesh] OR "Macau"[Mesh] OR "Malta"[Mesh] OR "Monaco"[Mesh] OR "Netherlands"[Mesh] OR "New Caledonia"[Mesh] OR "New Zealand"[Mesh] OR "New Zealand*"[tw] OR "Northern Mariana Island*"[tw] OR "Norway"[Mesh] OR "Oman"[Mesh] OR "Panama"[Mesh] OR "Poland"[Mesh] OR "Polynesia"[Mesh] OR "Portugal"[Mesh] OR "Puerto Rico"[Mesh] OR "Qatar"[Mesh] OR "Republic of Korea"[Mesh] OR "Romania"[Mesh] OR "Saint Kitts and Nevis"[Mesh] OR "Saint Kitts Nevis"[tiab:~1] OR "Saint Maarten*"[tw] OR "Saint Martin*"[tw] OR "San Marino"[Mesh] OR "San Marino*"[tw] OR "Saudi Arabia"[Mesh] OR "Saudi Arabia*"[tw] OR "Scotland"[Mesh] OR "Seychelles"[Mesh] OR "Singapore"[Mesh] OR "Slovakia"[Mesh] OR "Slovenia"[Mesh] OR "Spain"[Mesh] OR "Sweden"[Mesh] OR "Switzerland"[Mesh] OR "Taiwan"[Mesh] OR "Thailand"[Mesh] OR "Trinidad and Tobago"[Mesh] OR "Trinidad Tobago"[tiab:~1] OR "Turks Caicos"[tiab:~1] OR "U K"[tw] OR "UK"[tw] OR "United Arab Emirates"[Mesh] OR "United Arab Emirates"[tw] OR "United Kingdom"[Mesh] OR "United Kingdom"[tw] OR "United States Virgin Islands"[Mesh] OR "Uruguay"[Mesh] OR "Virgin Island*"[tw] OR Andorra*[tw] OR Aruba*[tw] OR Australia*[tw] OR Austria*[tw] OR Bahama*[tw] OR Bahrain*[tw] OR Barbados*[tw] OR Belgian*[tw] OR Belgium[tw] OR Bermuda*[tw] OR Britain[tw] OR British[tw] OR Brunei*[tw] OR Canada[tw] OR Canadian*[tw] OR Chile*[tw] OR Croatia*[tw] OR Curaçao*[tw] OR Cyprus*[tw] OR Dane*[tw] OR Danish[tw] OR Denmark[tw] OR Dutch*[tw] OR England[tw] OR English[tw] OR Estonia*[tw] OR Finland[tw] OR Finn*[tw] OR France[tw] OR French*[tw] OR German*[tw] OR Gibraltar*[tw] OR Greece*[tw] OR Greenland*[tw] OR Guam*[tw] OR Guernsey*[tw] OR Hungary*[tw] OR Iceland*[tw] OR Ireland[tw] OR Irish*[tw] OR Israel*[tw] OR Italian*[tw] OR Italy[tw] OR Japan*[tw] OR Korea*[tw] OR Kuwait*[tw] OR Latvia*[tw] OR Liechtenstein*[tw] OR Lithuania*[tw] OR Luxembourg*[tw] OR Macao*[tw] OR Malta*[tw] OR Monaco*[tw] OR Nauru*[tw] OR Netherlands[tw] OR New Caledonia*[tw] OR Norway[tw] OR Norwegian*[tw] OR Oman*[tw] OR Panama*[tw] OR Poland[tw] OR Pole*[tw] OR Polish[tw] OR Portugal[tw] OR Portuguese[tw] OR Puerto Rico*[tw] OR Qatar*[tw] OR Romania*[tw] OR Scot*[tw] OR Scotland[tw] OR Seychelle*[tw] OR Singapore*[tw] OR Slovakia*[tw] OR Slovenia*[tw] OR Spain[tw] OR Spaniard*[tw] OR Spanish[tw] OR Sweden[tw] OR Swedish[tw] OR Swiss[tw] OR Switzerland[tw] OR Taiwan*[tw] OR Thai*[tw] OR Uruguay*[tw] | 5,394,188 |
| **UK-Filter** | 17 | "United Kingdom"[Mesh] | 390,209 |
|  | 18 | ("national health service*"[tiab] OR nhs[tiab] OR "national health service*"[ad] OR nhs[ad]) | 270,709 |
|  | 19 | (english[tiab] NOT ("published english"[tiab:~5] OR "publication english"[tiab:~5] OR "translate english"[tiab:~5] OR "translated english"[tiab:~5] OR "written english"[tiab:~5] OR "language english"[tiab:~5] OR "speak english"[tiab:~5] OR "literature english"[tiab:~5] OR "citation english"[tiab:~5])) | 64,080 |
|  | 20 | (gb[tiab] OR "g.b."[tiab] OR britain*[tiab] OR (british*[tiab] NOT "british columbia"[tiab]) OR uk[tiab] OR "u.k."[tiab] OR united kingdom*[tiab] OR (england*[tiab] NOT "new england"[tiab:~0]) OR northern ireland*[tiab] OR northern irish*[tiab] OR scotland*[tiab] OR scottish*[tiab] OR ((wales[tiab] OR "south wales"[tiab:~0]) NOT "new south wales"[tiab:~0]) OR welsh*[tiab]) | 326,654 |
|  | 21 | (bath[tiab] OR "bath's"[tiab] OR ((birmingham[tiab] NOT alabama*[tiab]) OR ("birmingham's"[tiab] NOT alabama*[tiab]) OR bradford[tiab] OR "bradford's"[tiab] OR brighton[tiab] OR "brighton's"[tiab] OR bristol[tiab] OR "bristol's"[tiab] OR carlisle*[tiab] OR "carlisle's"[tiab] OR (cambridge[tiab] NOT (massachusetts*[tiab] OR boston*[tiab] OR harvard*[tiab])) OR ("cambridge's"[tiab] NOT (massachusetts*[tiab] OR boston*[tiab] OR harvard*[tiab])) OR (canterbury[tiab] NOT zealand*[tiab]) OR ("canterbury's"[tiab] NOT zealand*[tiab]) OR chelmsford[tiab] OR "chelmsford's"[tiab] OR chester[tiab] OR "chester's"[tiab] OR chichester[tiab] OR "chichester's"[tiab] OR coventry[tiab] OR "coventry's"[tiab] OR derby[tiab] OR "derby's"[tiab] OR (durham[tiab] NOT (carolina*[tiab] OR nc[tiab])) OR ("durham's"[tiab] NOT (carolina*[tiab] OR nc[tiab])) OR ely[tiab] OR "ely's"[tiab] OR exeter[tiab] OR "exeter's"[tiab] OR gloucester[tiab] OR "gloucester's"[tiab] OR hereford[tiab] OR "hereford's"[tiab] OR hull[tiab] OR "hull's"[tiab] OR lancaster[tiab] OR "lancaster's"[tiab] OR leeds*[tiab] OR leicester[tiab] OR "leicester's"[tiab] OR (lincoln[tiab] NOT nebraska*[tiab]) OR ("lincoln's"[tiab] NOT nebraska*[tiab]) OR (liverpool[tiab] NOT (new south wales*[tiab] OR nsw[tiab])) OR ("liverpool's"[tiab] NOT (new south wales*[tiab] OR nsw[tiab])) OR ((london[tiab] NOT (ontario*[tiab] OR ont[tiab] OR toronto*[tiab])) OR ("london's"[tiab] NOT (ontario*[tiab] OR ont[tiab] OR toronto*[tiab])) OR manchester[tiab] OR "manchester's"[tiab] OR (newcastle[tiab] NOT (new south wales*[tiab] OR nsw[tiab])) OR ("newcastle's"[tiab] NOT (new south wales*[tiab] OR nsw[tiab])) OR norwich[tiab] OR "norwich's"[tiab] OR nottingham[tiab] OR "nottingham's"[tiab] OR oxford[tiab] OR "oxford's"[tiab] OR peterborough[tiab] OR "peterborough's"[tiab] OR plymouth[tiab] OR "plymouth's"[tiab] OR portsmouth[tiab] OR "portsmouth's"[tiab] OR preston[tiab] OR "preston's"[tiab] OR ripon[tiab] OR "ripon's"[tiab] OR salford[tiab] OR "salford's"[tiab] OR salisbury[tiab] OR "salisbury's"[tiab] OR sheffield[tiab] OR "sheffield's"[tiab] OR southampton[tiab] OR "southampton's"[tiab] OR st albans[tiab] OR stoke[tiab] OR "stoke's"[tiab] OR sunderland[tiab] OR "sunderland's"[tiab] OR truro[tiab] OR "truro's"[tiab] OR wakefield[tiab] OR "wakefield's"[tiab] OR wells[tiab] OR westminster[tiab] OR "westminster's"[tiab] OR winchester[tiab] OR "winchester's"[tiab] OR wolverhampton[tiab] OR "wolverhampton's"[tiab] OR (worcester[tiab] NOT (massachusetts*[tiab] OR boston*[tiab] OR harvard*[tiab])) OR ("worcester's"[tiab] NOT (massachusetts*[tiab] OR boston*[tiab] OR harvard*[tiab])) OR (york[tiab] NOT ("new york*"[tiab] OR ny[tiab] OR ontario*[tiab] OR ont[tiab] OR toronto*[tiab])) OR ("york's"[tiab] NOT ("new york*"[tiab] OR ny[tiab] OR ontario*[tiab] OR ont[tiab] OR toronto*[tiab]))))) | 195,029 |
|  | 22 | (bangor[tiab] OR "bangor's"[tiab] OR cardiff[tiab] OR "cardiff's"[tiab] OR newport[tiab] OR "newport's"[tiab] OR "st asaph"[tiab] OR "st asaph's"[tiab] OR st davids[tiab] OR swansea[tiab] OR "swansea's"[tiab]) | 3,417 |
|  | 23 | (aberdeen[tiab] OR "aberdeen's"[tiab] OR dundee[tiab] OR "dundee's"[tiab] OR edinburgh[tiab] OR "edinburgh's"[tiab] OR glasgow[tiab] OR "glasgow's"[tiab] OR inverness[tiab] OR (perth[tiab] NOT australia*[tiab]) OR ("perth's"[tiab] NOT australia*[tiab]) OR stirling[tiab] OR "stirling's"[tiab]) | 41,936 |
|  | 24 | (armagh[tiab] OR "armagh's"[tiab] OR belfast[tiab] OR "belfast's"[tiab] OR lisburn[tiab] OR "lisburn's"[tiab] OR londonderry[tiab] OR "londonderry's"[tiab] OR derry[tiab] OR "derry's"[tiab] OR newry[tiab] OR "newry's"[tiab]) | 1,559 |
|  | 25 | #17 OR #18 OR #19 OR #20 OR #21 OR #22 OR #23 OR #24 | 991,125 |
| **Canada-Filter** | 26 | Canada[Mesh] OR Canada[tw] OR Canad*[tw] | 253,141 |
|  | 27 | ("British Columbia"[tiab] OR "Colombie Britannique"[tiab] OR Alberta*[tiab] OR Saskatchewan[tiab] OR Manitoba*[tiab] OR Ontario[tiab] OR Quebec[tiab] OR "Nouveau Brunswick"[tiab] OR "New Brunswick"[tiab] OR "Nova Scotia"[tiab] OR "Nouvelle Ecosse"[tiab] OR "Prince Edward Island"[tiab] OR Newfoundland[tiab] OR Labrador[tiab] OR Nunavut[tiab] OR NWT[tiab] OR "Northwest Territories"[tiab] OR Yukon[tiab] OR Nunavik[tiab] OR Inuvialuit[tiab]) | 86,049 |
|  | 28 | Abbotsford[tiab] OR "Ajax"[tiab] OR "Aurora"[tiab] OR "Barrie"[tiab] OR "Belleville"[tiab] OR "Blainville"[tiab] OR "Brampton"[tiab] OR "Brantford"[tiab] OR "Brossard"[tiab] OR "Burlington"[tiab] OR "Burnaby"[tiab] OR "Caledon"[tiab] OR "Calgary"[tiab] OR "cape breton"[tiab] OR "chatham kent"[tiab] OR "Chilliwack"[tiab] OR "Clarington"[tiab] OR "Coquitlam"[tiab] OR "Drummondville"[tiab] OR "Edmonton"[tiab] OR "Fredericton"[tiab] OR "fort mcmurray"[tiab] OR "Gatineau"[tiab] OR "Granby"[tiab] OR "grande prairie"[tiab] OR "Guelph"[tiab] OR ("Halton Hills"[tiab]) OR "Iqaluit"[tiab] OR "Inuvik"[tiab] OR "Kamloops"[tiab] OR "kawartha lakes"[tiab] OR "Kelowna"[tiab] OR "Kingston"[tiab] OR "Kitchener"[tiab] OR "Langley"[tiab] OR "Laval"[tiab] OR "Lethbridge"[tiab] OR "Levis"[tiab] OR "Longueuil"[tiab] OR "maple ridge"[tiab] OR "Markham"[tiab] OR "medicine hat"[tiab] OR "Milton"[tiab] OR "Mirabel"[tiab] OR "Mississauga"[tiab] OR "Moncton"[tiab] OR "Montreal"[tiab] OR "Nanaimo"[tiab] OR "new westminster"[tiab] OR "Newmarket"[tiab] OR "niagara falls"[tiab] OR "norfolk county"[tiab] OR "north bay"[tiab] OR "north vancouver"[tiab] OR "north vancouver"[tiab] OR "Oakville"[tiab] OR "Oshawa"[tiab] OR "Ottawa"[tiab] OR "port coquitlam"[tiab] OR "prince george"[tiab] OR "quebec city"[tiab] OR "red deer"[tiab] OR "Regina"[tiab] OR "Repentigny"[tiab] OR "Saanich"[tiab] OR "Saguenay"[tiab] OR "saint john"[tiab] OR "Saint-Hyacinthe"[tiab] OR "Saint-Jean-sur-Richelieu"[tiab] OR "Saint-Jerome"[tiab] OR "Sarnia"[tiab] OR "Saskatoon"[tiab] OR "sault ste marie"[tiab] OR "Sherbrooke"[tiab] OR "st albert"[tiab] OR "st catharines"[tiab] OR "st john s"[tiab] OR ("Strathcona County"[tiab:~2]) OR "Terrebonne"[tiab] OR "thunder bay"[tiab] OR "Toronto"[tiab] OR "Trois-Rivieres"[tiab] OR "Vancouver"[tiab] OR "Vaughan"[tiab] OR (("Airdrie"[tiab] OR "Cambridge"[tiab] OR ("Halifax"[tiab] OR "Hamilton"[tiab] OR "London"[tiab] OR "Peterborough"[tiab] OR "Pickering"[tiab] OR "Richmond"[tiab] OR "richmond hill"[tiab] OR "Sudbury"[tiab] OR "Surrey"[tiab] OR "Victoria"[tiab] OR "Waterloo"[tiab] OR "Welland"[tiab] OR "Whitby"[tiab] OR "Windsor"[tiab])) NOT ("UK"[tw] OR "Britain"[tw] OR "united kingdom"[tw] OR "England"[tw] OR "Australia"[tw])) OR "Whitehorse"[tiab] OR "Winnipeg"[tiab] OR "wood buffalo"[tiab] OR "Yellowknife"[tiab] | 136,555 |
|  | 29 | #26 OR #27 OR #28 | 382,287 |
| **Spain-Filter** | 30 | ((spain OR espagne OR espana OR spagna) OR (spain[ad] OR espagne[ad] OR espana[ad] OR spanien[ad] OR spagna[ad]) OR (catalunya[ad] OR catalonia[ad] OR catalogne[ad] OR cataluna[ad] OR catala[ad] OR barcelon*[ad] OR tarragona[ad] OR lleida[ad] OR lerida[ad] OR girona[ad] OR gerona[ad] OR sabadell[ad] OR hospitalet[ad] OR l’hospitalet[ad]) OR (valencia*[ad] OR castello*[ad] OR alacant[ad] OR alicant*[ad]) OR (murcia*[ad] OR (cartagen*[ad] NOT indias[ad])) OR (andalu*[ad] OR sevill*[ad] OR granad*[ad] OR huelva[ad] OR almeria[ad] OR cadiz[ad] OR jaen[ad] OR malaga[ad] OR (cordoba[ad] NOT argentin*[ad])) OR (extremadura[ad] OR caceres[ad] OR badajoz[ad] OR madrid[ad]) OR (castilla[ad] OR salamanca[ad] OR zamora[ad] OR valladolid[ad] OR segovia[ad] OR soria[ad] OR palencia[ad] OR avila[ad] OR burgos[ad]) OR (leon[ad] NOT (france[ad] OR clermont[ad] OR rennes[ad] OR lyon[ad] OR USA[ad] OR mexic*[ad])) OR (galicia[ad] OR gallego[ad] OR compostela[ad] OR vigo[ad] OR corun*[ad] OR ferrol[ad] OR orense[ad] OR ourense[ad] OR pontevedra[ad] OR lugo[ad]) OR (oviedo[ad] OR gijon[ad] OR asturia*[ad]) OR (cantabr*[ad] OR santander[ad]) OR (vasco[ad] OR euskadi[ad] OR basque[ad] OR bilbao[ad] OR bilbo[ad] OR donosti*[ad] OR san sebastian[ad] OR vizcaya[ad] OR bizkaia[ad] OR guipuzcoa[ad] OR gipuzkoa[ad] OR alava[ad] OR araba[ad] OR vitoria[ad] OR gasteiz[AD]) OR (navarr*[ad] OR nafarroa[ad] OR pamplona[ad] OR iruna[ad] OR irunea[ad]) OR (logron*[ad] OR rioj*[ad]) OR (aragon*[ad] OR zaragoza[ad] OR teruel[ad] OR huesca[ad]) OR (mancha[ad] OR ciudad real[ad] OR albacete[ad] OR cuenca[ad]) OR (toledo[ad] NOT (ohio[ad] OR us[ad] OR usa[ad] OR OH[ad])) OR (guadalajara[ad] NOT mexic*[ad]) OR (balear*[ad] OR mallorca[ad] OR menorca[ad] OR ibiza[ad] OR eivissa[ad]) OR (palmas[ad] OR lanzarote[ad] OR canari*[ad] OR tenerif*[ad]) OR (ceuta[ad] OR melilla[ad])) OR (osasunbide*[ad] OR osakidetza[ad] OR insalud[ad] OR sergas[ad] OR catsalut[ad] OR sespa[ad] OR osasunbidea[ad] OR imsalud[ad] OR sescam[ad] OR ib-salut[ad]) | 803,004 |
| **Restrict to World Bank 'High-Income' Countries** | 31 | #15 AND (#16 OR #25 OR #29 OR #30) | 300 |
| **Restrict to Non-US /  Non-African countries** | 32 | #31 NOT ((africa[mesh] OR north america[mesh:noexp] OR united states[mesh]) NOT (asia[mesh] OR australia[mesh] OR canada[mesh] OR europe[mesh] OR south america[mesh])) | **277** |

# **Supplementary Table 3c. Search Terms for PrEP Effectiveness Sources (Cochrane Library)**

| **#** | **Search Terms** | **Number of Hits  (July 2023)** |
| --- | --- | --- |
| 1 | MeSH descriptor: [Pre-Exposure Prophylaxis] explode all trees | 395 |
| 2 | MeSH descriptor: [Chemoprevention] explode all trees | 2419 |
| 3 | pre‐exposure NEAR/2 prophylaxis OR preexposure NEAR/2 prophylaxis OR PREP OR anti‐retroviral NEAR/2 chemoprophylaxis OR antiretroviral NEAR/2 chemoprophylaxis OR chemoprevention OR HIV NEAR/2 prophylaxis | 4504 |
| 4 | MeSH descriptor: [Emtricitabine, Tenofovir Disoproxil Fumarate Drug Combination] explode all trees | 207 |
| 5 | 'emtricitabine tenofovir alafenamide' | 463 |
| 6 | fixed dose NEAR/1 emtricitabine tenofovir OR 'cabotegravir' NEAR/1 'extended-release' OR Truvada OR Descovy OR Apretude | 272 |
| 7 | #1 OR #2 OR #3 OR #4 OR #5 OR #6 | 7080 |
| 8 | MeSH descriptor: [HIV Infections] explode all trees and with qualifier(s): [prevention & control - PC] | 3149 |
| 9 | HIV NEAR/5 prevent OR HIV NEAR/5 prevents OR HIV NEAR/5 prevention OR human immunodeficiency virus NEAR/5 prevent OR human immunodeficiency virus NEAR/5 prevents OR human immunodeficiency virus NEAR/5 prevention | 4874 |
| 10 | #8 OR #9 | 6353 |
| 11 | MeSH descriptor: [Treatment Outcome] explode all trees | 181201 |
| 12 | effectiv* OR efficacious* OR efficacy OR efficiency OR outcome* OR response OR responsiveness | 1E+06 |
| 13 | #11 OR #12 | 1E+06 |
| 14 | #7 AND #10 AND #13 | 697 |
| 15 | #14 with Publication Year from 2017 to 2023, in Trials | 457 |
| 16 | MeSH descriptor: [Randomized Controlled Trial] explode all trees | 25732 |
| 17 | MeSH descriptor: [Randomized Controlled Trials as Topic] explode all trees | 47396 |
| 18 | MeSH descriptor: [Controlled Clinical Trial] explode all trees | 38477 |
| 19 | MeSH descriptor: [Controlled Clinical Trials as Topic] explode all trees | 47802 |
| 20 | MeSH descriptor: [Review] explode all trees | 1002 |
| 21 | MeSH descriptor: [Review Literature as Topic] explode all trees | 435 |
| 22 | #16 OR #17 OR #18 OR #19 OR #20 OR #21 | 86578 |
| 23 | #15 NOT #22 | 425 |
| 24 | ('animal cell' OR 'animal experiment' OR 'animal health' OR 'animal model' OR 'animal tissue' OR 'bird' OR 'cancer model' OR 'canine' OR 'dog' OR 'ex vivo study' OR 'goat' OR 'Hep-G2 cell line' OR 'in vitro study' OR 'knockout mouse' OR 'Kupffer cell' OR 'mammal' OR 'model' OR 'mouse' OR 'mouse model' OR 'murine' OR mice OR 'nonhuman' OR 'nude rat' OR 'rat' OR 'rat model' OR 'tumor cell' OR 'tumor model' OR 'tumor xenograft' OR 'veterinary') | 200247 |
| 25 | #23 NOT #24 | 261 |
| 26 | MeSH descriptor: [Africa] explode all trees | 11427 |
| 27 | MeSH descriptor: [North America] this term only | 715 |
| 28 | MeSH descriptor: [United States] explode all trees | 27438 |
| 29 | MeSH descriptor: [Asia] explode all trees | 33380 |
| 30 | MeSH descriptor: [Australia] explode all trees | 6637 |
| 31 | MeSH descriptor: [Canada] explode all trees | 5612 |
| 32 | MeSH descriptor: [Europe] explode all trees | 41960 |
| 33 | MeSH descriptor: [South America] explode all trees | 3876 |
| 34 | (#26 OR #27 OR #28) NOT (#29 OR #30 Or #31 OR 49 OR #33) | 34386 |
| 35 | #25 NOT #34 | **218** |

# **Supplementary Table 4a. Grey Literature Results for Hand-Searching Abstracts of Relevant Congresses**

| **Search Topic/Congress** | **Number of Hits** |
| --- | --- |
| HIV-1 Epidemiology (January 2017– July 2023) |  |
| AIDS | 9 |
| BHIVA | 17 |
| CROI | 2 |
| HIV Glasgow | 5 |
| IAS | 2 |
| Prevalence of PrEP Use (January 2018– July 2023) |  |
| AIDS | 4 |
| BASHH | 11 |
| BHIVA | 12 |
| CROI | 1 |
| IAS | 2 |
| PrEP Effectiveness (January 2018– July 2023) |  |
| AIDS | 6 |
| BASHH | 1 |
| BHIVA | 8 |
| CROI | 2 |
| HIV Glasgow | 3 |
| IAS | 6 |

AIDS, International AIDS Conference; BASHH, British Association for Sexual Health Annual Conference; BHIVA, British HIV Association Conference; CROI, Conference on Retroviruses and Opportunistic Infections; IAS, International AIDS Society Conference; HIV Glasgow, HIV Drug Therapy Glasgow Conference; PrEP, pre-exposure prophylaxis.

# **Supplementary Table 4b. Search Strategies for Local Grey Literature Searches**

| **Country** | **Link** | **Search Strategy** | **Search Terms** | **Number of Hits (November 2023)** |
| --- | --- | --- | --- | --- |
| **France** | <https://vih.org/> | Used search functionality to search associated search terms. | l’incidence du VIH | 90 |
|  |  |  | prévalence du VIH | 231 |
|  |  |  | PrEP | 445 |
|  | <https://www.santepubliquefrance.fr/> | Used search functionality to search associated search terms. | l’incidence du VIH | 3 |
|  |  |  | prévalence du VIH | 9 |
|  |  |  | PrEP | 17 |
|  | <https://actions-traitements.org> | Used search functionality to search associated search terms. | l’incidence du VIH | 50 |
|  |  |  | prévalence du VIH | 20 |
|  |  |  | prévalence de l’utilisation de la PrEP | 150 |
|  |  |  | nouvelle utilisation de la PrEP | 170 |
|  |  |  | La PrEP et l’acceptabilité | 10 |
|  |  |  | La PrEP et l’abordabilité | 0 |
|  |  |  | Efficacité de la PrEP | 110 |
|  |  |  | Risque de VIH et PrEP | 200 |
|  |  |  | Obstacles à la PrEP | 20 |
|  | <https://www.trt-5.org/enquetes-et-observatoires/> | Used search functionality to search associated search terms. | prévalence du VIH | 30 |
|  |  |  | prévalence de l’utilisation de la PrEP | 2 |
|  |  |  | nouvelle utilisation de la PrEP | 20 |
|  |  |  | La PrEP et l’acceptabilité | 0 |
|  |  |  | La PrEP et l’abordabilité | 0 |
|  |  |  | Efficacité de la PrEP | 30 |
|  |  |  | Risque de VIH et PrEP | 40 |
|  |  |  | Obstacles à la PrEP | 2 |
|  | <https://seronet.info/article/prep-en-france-ou-en-est-92647/> | Used search functionality to search associated search terms. | l’incidence du VIH | 150 |
|  |  |  | prévalence du VIH | 170 |
|  |  |  | PrEP | 480 |
|  | <https://www.epi-phare.fr/en/study-reports-and-publications/> | Used search functionality to search associated search terms. | HIV | 8 |
| **UK and Ireland** | <https://www.gov.uk/government/statistics/announcements/national-hiv-data-tables-data-up-until-december-2022/> | Included most recent data on this site | - | 19 |
|  | <https://www.hiv-lens.org/> | Single source | - | 1 |
|  | [www.hpsc.ie/](http://www.hpsc.ie/) | Used search functionality to search associated search terms. | incidence of HIV | 206 |
|  |  |  | prevalence of HIV | 171 |
|  |  |  | PrEP | 56 |
|  | www.publichealth.hscni.net | Used search functionality to search associated search terms. | incidence of HIV | 3 |
|  |  |  | prevalence of HIV | 10 |
|  |  |  | PrEP | 7 |
|  | <https://publichealthscotland.scot/> | Used search functionality to search associated search terms. | incidence of HIV | 0 |
|  |  |  | prevalence of HIV | 2 |
|  |  |  | prevalence of PrEP use | 0 |
|  |  |  | PrEP | 7 |
| **Canada** | <https://www.canada.ca/en/services/health.html> | Used search functionality to search associated search terms. | incidence of HIV | 100 |
|  |  |  | pre-exposure prophylaxis | 94 |
|  | <https://www.ohtn.on.ca/> | Used search functionality to search associated search terms. | PrEP | 110 |
|  | <https://www.ohesi.ca/reports> | No terms needed, search all 8 reports on the website | - | 8 |
|  | <https://www.bccfe.ca/centre-documents/prep-semi-annual-report-british-columbia-fourth-quarter-2022/> | Single source | - | 1 |
|  | [www.advance-avancer.ca/progress-cards/](http://www.advance-avancer.ca/progress-cards/) | All 10 progress cards searched | - | 10 |
| **Spain** | <https://www.sanidad.gob.es/ciudadanos/enfLesiones/enfTransmisibles/sida/PrEP.htm> | Used search functionality to search associated search terms. | PrEP | 176 |
|  | <https://siprep.isciii.es/documentos/> | All available sources searched on “documents” page | - | 9 |
| **Italy** | <https://www.epicentro.iss.it/aids/epidemiologia-italia> | Used search functionality to search associated search terms. | incidenza dell'HIV | 100 |
|  |  |  | prevalenza dell'HIV | 100 |
|  |  |  | PrEP | 47 |
|  | <https://www.iss.it/documents/20126/5278313/COA+2022.pdf/b518925d-7663-cc23-610f-5f98b23a565d?t=1669622871324> | Single source | - | 1 |
|  | <https://www.ecdc.europa.eu/en/publications-data/hiv-infection-prevention-pre-exposure-prophylaxis-monitoring-dublin/> | Single source | - | 1 |
| **Australia** | <https://www.kirby.unsw.edu.au/research/projects/ahod> | Used search functionality to search associated search terms. | HIV | 978 |
|  |  |  | PrEP | 196 |
|  | <https://www.kirby.unsw.edu.au/sites/default/files/documents/Monitoring-HIV-PrEP-uptake-in-Australia-newsletter_Issue8.pdf> | Single source | - | 1 |
| **Belgium** | <https://www.sciensano.be/en/biblio/epidemiologie-van-aids-en-hiv-infectie-belgie-toestand-op-31-december-2021> | Single source | - | 1 |
|  | <https://www.sciensano.be/nl/biblio/epidemiologie-van-aids-en-hiv-infectie-belgie-toestand-op-31-december-2021> | Used search functionality to search associated search terms. | HIV | 937 |
|  |  |  | PrEP | 196 |
|  |  |  | prevalentie van PrEP-gebruik | 18 |
|  |  |  | nieuw PrEP-gebruik | 20 |
|  |  |  | HIV-risico en PrEP | 33 |
|  |  |  | belemmeringen voor PrEP | 6 |
|  | ÉPIDÉMIOLOGIE DU SIDA ET DE L’INFECTION À VIH EN BELGIQUE, RAPPORT 2022, SITUATION AU 31 DÉCEMBRE 2021 (internal resource) | Single source | - | 1 |
| **Nordics** | <https://www.ssi.dk/sygdomme-beredskab-og-forskning/sygdomsovervaagning/h/hiv-2022> | Used search functionality to search associated search terms. | udbredelse af PrEP-brug | 2 |
|  |  |  | ny PrEP-brug | 10 |
|  |  |  | HIV-risiko og PrEP | 7 |
|  |  |  | barrierer for PrEP | 0 |
|  |  |  | HIV | 33 |
|  |  |  | PrEP | 13 |
|  |  |  | Single source | 1 |
|  | <https://www.ncbi.nlm.nih.gov/pmc/articles/PMC7045051/> | Single source | - | 1 |
|  | <https://www.rfsl.se/wp-content/uploads/2021/08/FINAL_PrEP_RAPPORT_WEBB_LQ.pdf> | Single source | - | 1 |
|  | [Hivinfektion – sjukdomsstatistik — Folkhälsomyndigheten (folkhalsomyndigheten.se)](https://www.folkhalsomyndigheten.se/folkhalsorapportering-statistik/statistik-a-o/sjukdomsstatistik/hivinfektion/) | Single source | - | 1 |
|  | <https://www.folkhalsomyndigheten.se/the-public-health-agency-of-sweden/> | Used search functionality to search associated search terms. | prevalens av PrEP-användning | 0 |
|  |  |  | ny användning av PrEP | 12 |
|  |  |  | HIV-risk och PrEP | 25 |
|  |  |  | hinder för PrEP | 13 |
|  |  |  | HIV | 360 |
|  |  |  | PrEP | 28 |
|  | <https://www.diva-portal.org/smash/get/diva2:1684908/ATTACHMENT01.pdf> | Single source | - | 1 |
| **Germany** | <https://www.rki.de/DE/Content/InfAZ/H/HIVAIDS/Abschlussbericht_EvE-PrEP.pdf?__blob=publicationFile/> | Single source | - | 1 |
|  | <https://pubmed.ncbi.nlm.nih.gov/31583823/> | Single source | - | 1 |
|  | <https://www.eurosurveillance.org/content/10.2807/1560-7917.ES.2019.24.7.1800398/> | Single source | - | 1 |
|  | <https://www.rki.de/DE/Content/Infekt/EpidBull/Archiv/2022/47/Art_01.html> | Single source | - | 1 |
| **Japan** | <https://api-net.jfap.or.jp/index.html> | Used search functionality to search associated search terms. | PrEP使用の有病率 | 46 |
|  |  |  | 新しいPrEPの使用 | 130 |
|  |  |  | HIVリスクとPrEP | 91 |
|  |  |  | PrEPに対する障壁 | 46 |
|  | <https://api-net.jfap.or.jp/status/japan/nenpo.html> | Single source | - | 1 |
|  | <https://prep.ptokyo.org/wp/wp-content/uploads/2022/04/prepinjapan_report_R3.pdf> | Single source | - | 1 |
|  | <https://jaids.jp/wpsystem/wp-content/uploads/2022/11/tebiki-1Pver.pdf> | Single source | - | 1 |
|  | <https://jaids.jp/wpsystem/wp-content/uploads/2022/11/uder-guide-matome-1Pver.pdf> | Single source | - | 1 |
| **Netherlands** | <https://www.hiv-monitoring.nl/en/resources/hiv-netherlands> | Used search functionality to search associated search terms. | HIV | 180 |
|  |  |  | PrEP | 20 |
|  | <https://www.hiv-monitoring.nl/en/resources/monitoring-report-2023> | Single source | - | 1 |
|  | <https://www.gezondheidsraad.nl/documenten/adviezen/2018/03/27/preventief-gebruik-van-hiv-remmers> | Single source | - | 1 |
|  | <https://www.gezondheidsraad.nl/> | Used search functionality to search associated search terms. | HIV | 95 |
|  |  |  | PrEP | 18 |
|  | <https://www.soaaids.nl/files/2022-07/20220711-PrEP-richtlijn-Nederland-versie-3-update-2022.pdf> | Single source | - | 1 |
|  | <https://wetten.overheid.nl/BWBR0042434/2023-01-01> | Single source | - | 1 |
|  | <https://www.rijksoverheid.nl/documenten/kamerstukken/2023/02/13/kamerbrief-over-voortgang-beleidsvisie-seksuele-gezondheid> | Single source | - | 1 |
|  | <https://www.rijksoverheid.nl/> | Used search functionality to search associated search terms. | HIV | 66 |
|  |  |  | PrEP | 26 |
|  | <https://www.rijksoverheid.nl/documenten/rapporten/2023/09/25/scenarioverkenning-prep> | Single source | - | 1 |
|  | <https://www.rijksoverheid.nl/documenten/rapporten/2023/07/27/cijfermatige-eindevaluatie-prep-pilot> | Single source | - | 1 |
|  | <https://richtlijnen.nhg.org/standaarden/het-soa-consult> | Single source | - | 1 |
| **Portugal** | <https://www.insa.min-saude.pt/?s=PrEP> | Used search functionality to search associated search terms. | PrEP | 350 |
|  |  |  | VIH | 80 |
|  | insa.min-saude.pt/relatorio-infecao-por-vih-em-portugal-2022/ | Single source | - | 1 |
| **Switzerland** | <https://www.bag.admin.ch/bag/de/home/zahlen-und-statistiken/zahlen-zu-infektionskrankheiten.exturl.htm>l | Single source | - | 1 |
|  | BAG Bulletin 45-2022 (internal resource) | Single source | - | 1 |
|  | <https://www.swissprepared.ch/en/> | Single source | - | 1 |
|  | Zahlen zu Infektionskrankheiten (internal resource) | Single source | - | 1 |
| **Croatia** | <https://bfm.hr/?s=HIV/> | Used search functionality to search associated search terms. | HIV | 50 |
|  |  | Used search functionality to search associated search terms. | PrEP | 50 |
|  | <https://www.hzjz.hr/> | Used search functionality to search associated search terms. | HIV | 180 |
|  |  |  | PrEP | 779 |
|  | <https://www.hzjz.hr/sluzba-epidemiologija-zarazne-bolesti/epidemiologija-hiv-infekcije-i-aids-a-u-hrvatskoj/> | Used search functionality to search associated search terms. | HIV | 180 |
|  |  |  | PrEP | 779 |
|  | <https://www.hzjz.hr/sluzba-epidemiologija-zarazne-bolesti/epidemiologija-hiv-infekcije-i-aids-a-u-hrvatskoj/> | Single source | - | 1 |
|  | <https://www.ecdc.europa.eu/sites/default/files/documents/HIV-pre-exposure-prophylaxis-evidence-2019.pdf> | Single source | - | 1 |
|  | <https://huhiv.hr/about-us/> | Used search functionality to search associated search terms. | HIV | 800 |
|  |  |  | PrEP | 500 |
|  | <https://region-hovedstaden-ekstern.23video.com/po3-3-bogdanicmp4mp4> | Single source | - | 1 |
| **Croatia, Estonia** | IQVIA Gilead EnDeMic HIV/HCV Market Landscape powerpoint (internal resource) | Single source | - | 1 |
| **Estonia** | <https://www.aidshealth.org/global/Estonia/> | Single source | - | 1 |
|  | <https://www.unaids.org/en/regionscountries/countries/estonia> | Single source | - | 1 |
|  | <https://www.hiv.ee/> | Used search functionality to search associated search terms. | HIV | 37 |
|  |  |  | PrEP | 7 |
|  | <https://balthiv.com/en/hivaids> | Single source | - | 1 |
|  | <https://www.sm.ee/en/news/estonian-government-adopted-action-plan-stop-hiv-epidemic> | Single source | - | 1 |

PrEP, pre-exposure prophylaxis.

# **Supplementary Table 4c. Search Strategies for International Grey Literature Searches**

| **Organization** | **Link** | **Search Strategy** | **Search Terms** | **Number of Reviewed Sources**  **(November 2023)** |
| --- | --- | --- | --- | --- |
| **European Centre for Disease Prevention and Control** | <https://www.ecdc.europa.eu/en> | Used search functionality to search associated search terms. | HIV incidence | 696 |
|  |  |  | HIV prevalence | 567 |
|  |  |  | PrEP | 190 |
|  |  |  | PrEP knowledge | 90 |
|  |  |  | PrEP acceptability | 92 |
| **Gov.uk** | <https://www.gov.uk/> | Used search functionality to search associated search terms. Searches were time limited for each term. The number of results reviewed is reported in the right-most column. | HIV incidence | 100 |
|  |  |  | HIV prevalence | 100 |
|  |  |  | PrEP | 100 |
|  |  |  | PrEP acceptability | 100 |
|  |  |  | PrEP knowledge | 100 |
| **Government of Canada** | <https://www.canada.ca/en.html> | Used search functionality to search associated search terms. Searches were time limited for each term. The number of results reviewed is reported in the right-most column. | HIV AND (incidence OR prevalence) | 50 |
|  |  |  | HIV incidence AND MSM | 154 |
|  |  |  | HIV incidence AND transgender | 267 |
|  |  |  | HIV incidence AND PWID | 21 |
|  |  |  | HIV incidence AND SW | 110 |
|  |  |  | HIV prevalence AND MSM | 100 |
|  |  |  | HIV prevalence AND transgender | 229 |
|  |  |  | HIV prevalence AND PWID | 27 |
|  |  |  | HIV prevalence AND SW | 50 |
|  |  |  | PrEP AND uptake | 50 |
|  |  |  | PrEP acceptability AND MSM | 11 |
|  |  |  | PrEP acceptability AND transgender | 38 |
|  |  |  | PrEP acceptability AND PWID | 4 |
|  |  |  | PrEP acceptability AND SW | 70 |
|  |  |  | PrEP knowledge AND MSM | 17 |
|  |  |  | PrEP knowledge AND transgender | 51 |
|  |  |  | PrEP knowledge AND PWID | 5 |
|  |  |  | PrEP knowledge AND SW | 50 |
|  |  |  | PrEP use AND MSM | 21 |
|  |  |  | PrEP use AND transgender | 50 |
|  |  |  | PrEP use AND PWID | 9 |
|  |  |  | PrEP use AND SW | 50 |
|  |  |  | PrEP incidence AND MSM | 50 |
|  |  |  | PrEP incidence AND transgender | 54 |
|  |  |  | PrEP incidence AND PWID | 7 |
|  |  |  | PrEP incidence AND SW | 50 |
| **Health Protection Surveillance Centre (Ireland)** | [Home - Health Protection Surveillance Centre (hpsc.ie)](https://www.hpsc.ie/) | Health topics A-Z > Human immunodeficiency virus (HIV) > HIV Data and Reports | - | 3 |
|  |  | Health topics A-Z > Human immunodeficiency virus (HIV) > Men who have sex with men (MSM) > epidemiology reports | - | 1 |
|  |  | Health topics A-Z > Human immunodeficiency virus (HIV) > Men who have sex with men (MSM) > people who inject drugs > Screened all included studies in Table 3 of the report "Drug-related bloodborne viruses in Ireland, 2018" (linked here) since this was the only report included on this webpage | - | 20 |
|  |  | Health topics A-Z > Human immunodeficiency virus (HIV) > HIV Treatment and PrEP | - | 1 |
| **UNAIDS** | https://aidsinfo.unaids.org/ | Main webpage (AIDSinfo) > Key Population | - | 1 |
|  |  | Main webpage (AIDSinfo) > Fact sheets > country fact sheets (searched fact sheets for each of the ~15 countries initially agreed upon with the study team) | - | 21 |
| **PrEPWatch** | <https://www.prepwatch.org/data-by-country/> | Navigated through all countries agreed upon with the study team | - | 21 |

MSM, men who have sex with men; PrEP, pre-exposure prophylaxis; PWID, people who inject drugs; SW, sex workers; UNAIDS, United Nations Political Declaration on HIV/AIDS.

# **Supplementary Table 5. Inclusion and Exclusion Criteria**

| **Domain** | **Inclusion** | **Exclusion** |
| --- | --- | --- |
| **Population** | Individuals who want or need PrEP (MSM, transgender people, PWID, people with high-risk sexual behavior [e.g., sex workers, HIV serodiscordant couples]) residing in high-income countries, as defined by the World Bank classification (other than the US and countries in Africa) | Individuals who do not want or need PrEP |
| **Intervention** | Oral PrEP regimens: DESCOVY^®^ (F/TAF), TRUVADA^®^ (F/TDF), or any generic brand of TRUVADA^®^ | Non-oral PrEP interventions |
| **Comparator** | Any or none | NA |
| **Outcomes** | Epidemiology outcomes:  Incidence of HIV-1 infection  Prevalence of HIV-1 infection  Prevalence of PrEP use outcomes:  Prevalence of PrEP use (proportion of PrEP users)  Incidence of PrEP use (proportion of new PrEP users)  PrEP effectiveness outcomes:  Incidence rate ratio  Measures of association (e.g., RR, OR, HR) comparing HIV-1 risk in those receiving PrEP vs. not receiving PrEP  Single arm studies reporting HIV-1 epidemiology outcomes in PrEP users | Any other outcome |
| **Study Design** | Studies reporting primary data, which are not RCTs, case reports, or case series  Studies which are not reporting single-site/clinic-level data | Studies without primary data  Studies that are RCTs, case reports, or case series  Studies that are single-site/clinic-level data |

HR, hazards ratio; MSM, men who have sex with men; NA, not applicable; OR, odds ratio; PrEP, pre-exposure prophylaxis; PWID, people who inject drugs; RCT, randomized controlled trial; RR, risk ratio; TAF, tenofovir alafenamide; TDF, tenofovir disoproxil fumarate.

# **Supplementary Table 6a. Criteria for Assessing Risk of Bias – HIV-1 Epidemiology**

| **DOMAIN 1: EXTERNAL VALIDITY  Is the sample representative of the population of interest?** | |
| --- | --- |
| **1.1 Was an appropriate sampling method used?** | |
| 1 | Probability-based sample (including: simple random, systematic, stratified, cluster, two-stage, and multi-stage sampling) Respondent-driven sampling or properly described time–location/venue sampling (if analyzed appropriately) |
| 0 | Non-random sample (including purposive, quota, convenience, and snowball), or sampling not described |
| **1.2 Were inclusion and exclusion criteria explicit and appropriate to the research question?** | |
| 1 | Yes, e.g. MSM, PWID, transgender people, etc |
| 0 | No |
| **DOMAIN 2: SELECTION (NON-RESPONSE) BIAS**  **Were the outcome data incomplete (due to non-response, refusal, or exclusion), and how did this affect the outcome?** | |
| **2.1 Was there bias in recruitment and enrollment of participants, selective participation, or differential follow-up?** | |
| 3 | Well-described methods of recruitment and enrollment; appropriate venue/location coverage; not relevant AND Participation rate is high and characteristics related to outcome measures do not significantly differ between participants and non-participants; low participation rate is managed appropriately; not relevant  AND Retention rate ≥80% and sociodemographic characteristics compared and not significantly different; not relevant |
| 2 | Meet any two of the criteria above |
| 1 | Meet any one of the criteria above |
| 0 | Meet none of the criteria above |
| **DOMAIN 3: MEASUREMENT BIAS** | |
| **3.1 Was a valid tool used for the identification of the outcome (i.e., HIV-1 infection)?** | |
| 1 | Serum test for HIV-1 diagnosis |
| 0 | Self-reported or observed by study personnel |
| **3.2 Was the outcome (i.e., HIV-1 infection) measured in a standard, reliable way?** | |
| 1 | Outcome measured systematically; data collectors appropriately trained |
| 0 | Unclear/inconsistent methods; non-systematic measurement or recording |
| **DOMAIN 4: INTERNAL VALIDITY  How likely could the result be due to chance? What is the level of precision?** | |
| **4.1 Was the sample size adequate?** | |
| 1 | ≥200 |
| 0 | <200 |
| **4.2 Was there appropriate statistical analysis?** | |
| 1 | Detailed statistical methods described Primarily consider the measure of risk that will be used in the meta-analysis – i.e., incidence rates, and/or incidence  For proportions (prevalence/cumulative incidence): denominator and numerator explicitly reported and appropriate/justified For incidence rates: calculation of person–years, explicitly reported and appropriate/justified (should not count HIV time towards total person–years) If calculated based on data from author: sufficient data provided for accurate calculation |
| 0 | Methods not sufficiently described; inappropriate technique |
| **DOMAIN 5: OTHER ISSUES** | |
| **5.1 Was HIV-1 incidence an objective of the study?** | |
| 1 | Yes (consider objectives of overall study, not sub-study/specific paper) |
| 0 | No (e.g., cohort may have been originally designed to measure STD incidence, but they also published a paper on HIV incidence) |
| **5.2 Were there any other issues that may have introduced bias or affected the validity of the estimates?** | |
| 1 | No issues |
| 0 | Study design issues, e.g., highly variable/skewed follow-up times in open-cohort study; very long follow-up period during which true incidence in the population likely to have changed Selective use or reporting of data (e.g., only reporting HIV-1 incidence in one subgroup or at one time point without justification) Intervention may impact on HIV-1 incidence e.g. ,PrEP use, or other prevention methods |

Quality was calculated as the total score for a source divided by the highest possible score (11 points).

HIV-1, human immunodeficiency virus-1; MSM, men who have sex with men; PrEP, pre-exposure prophylaxis; PWID, people who inject drugs; STD, sexually transmitted disease.

# **Supplementary Table 6b. Criteria for Assessing Risk of Bias – Prevalence of PrEP Use**

| **DOMAIN 1: EXTERNAL VALIDITY  Is the sample representative of the population of interest?** | |
| --- | --- |
| **1.1 Was an appropriate sampling method used?** | |
| 1 | Probability-based sample (including: simple random, systematic, stratified, cluster, two-stage, and multi-stage sampling) Respondent-driven sampling or properly described time–location/venue sampling (if analyzed appropriately) |
| 0 | Non-random sample (including purposive, quota, convenience, and snowball), or sampling not described |
| **1.2 Were inclusion and exclusion criteria explicit and appropriate to the research question?** | |
| 1 | Yes, e.g., MSM, PWID, transgender people, etc |
| 0 | No |
| **DOMAIN 2: SELECTION (NON-RESPONSE) BIAS**  **Was the outcome data incomplete (due to non-response, refusal, or exclusion), and how did this affect the outcome?** | |
| **2.1 Was there bias in recruitment and enrollment of participants, selective participation, or differential follow-up?** | |
| 3 | Well-described methods of recruitment and enrollment; appropriate venue/location coverage; not relevant AND Participation rate is high and characteristics related to outcome measures do not significantly differ between participants and non-participants; Low participation rate is managed appropriately; not relevant AND Retention rate ≥80% and sociodemographic characteristics compared and not significantly different; not relevant |
| 2 | Meet any two of the criteria above |
| 1 | Meet any one of the criteria above |
| 0 | Meet none of the criteria above |
| **DOMAIN 3: MEASUREMENT BIAS** | |
| **3.1 Was a valid tool used for the identification of the outcome (i.e., Prevalence of PrEP use)?** | |
| 1 | Prescription data or laboratory results used to identify PrEP use |
| 0 | Self-reported or observed by study personnel |
| **3.2 Was the outcome (i.e., Prevalence of PrEP use) measured in a standard, reliable way?** | |
| 1 | Outcome measured systematically; data collectors appropriately trained |
| 0 | Unclear/inconsistent methods; non-systematic measurement or recording |
| **DOMAIN 4: INTERNAL VALIDITY  How likely could the result be due to chance? What is the level of precision?** | |
| **4.1 Was the sample size adequate?** | |
| 1 | ≥200 |
| 0 | <200 |
| **4.2 Was there appropriate statistical analysis?** | |
| 1 | Detailed statistical methods described Primarily consider the measure of risk that will be used in the meta-analysis – i.e., incidence rates, and/or incidence proportion  For proportions (prevalence/cumulative incidence): denominator and numerator explicitly reported and appropriate/justified For incidence rates: calculation of person–years, explicitly reported and appropriate/justified (should not count PrEP time towards total person–years) If calculated based on data from author: sufficient data provided for accurate calculation |
| 0 | Methods not sufficiently described; inappropriate technique |
| **DOMAIN 5: OTHER ISSUES** | |
| **5.1 Was Prevalence of PrEP use an objective of the study?** | |
| 1 | Yes (consider objectives of overall study, not sub-study/specific paper) |
| 0 | No (e.g., cohort may have been originally designed to measure STD incidence, but they also published a paper on Prevalence of PrEP use) |
| **5.2 Were there any other issues that may have introduced bias or affected the validity of the estimates?** | |
| 1 | No issues |
| 0 | Study design issues, e.g., highly variable/skewed follow-up times in open-cohort study; very long follow-up period during which true incidence in the population likely to have changed Selective use or reporting of data (e.g., only reporting Prevalence of PrEP use in one subgroup or at one time point without justification) Access issues may impact on Prevalence of PrEP use e.g., not approved by the local government |

Quality was calculated as the total score for a source divided by the highest possible score (11 points).

MSM, men who have sex with men; PrEP, pre-exposure prophylaxis; PWID, people who inject drugs; STD, sexually transmitted disease.

# **Supplemental Table 7. Demographic Characteristics of Individuals Pooled Across Sources for all Three Outcomes**

|  | **All KP** | | **MSM** | | **PWID** | | **PrEP users** | |
| --- | --- | --- | --- | --- | --- | --- | --- | --- |
| **Demographic variable** | Study N | %  (95% CI) | Study N | %  (95% CI) | Study N | %  (95% CI) | Study N | %  (95% CI) |
| Male | 142 | 97.7  (97.0–98.3) | 130 | 100 | 16 | 74.2  (65.6–82.1) | 19 | 96.2  (88.5–100.0) |
| Age < 40 years old | 14 | 71.4  (60.2–81.5) | 10 | 70.2  (58.7–80.1) | 1 | 50.4* | 3 | 92.9  (38.9–100.0) |
| *Race/ethnicity* |  |  |  |  |  |  |  |  |
| White | 29 | 71.3  (66.6–75.7) | 21 | 71.2  (66.1–76.0) | 2 | 64.8* | 2 | 85.0* |
| Black | 15 | 8.7  (5.3–12.7) | 12 | 10.0  (5.4–15.8) | 0 | NA | 2 | 2.8* |
| Asian | 18 | 12.9  (6.8–20.6) | 18 | 16.3  (7.5–27.4) | 0 | NA | 2 | 2.7* |
| *Socioeconomic status* |  |  |  |  |  |  |  |  |
| Post-secondary degree and above | 46 | 60.4  (51.3–69.2) | 36 | 68.1  (64.7–71.4) | 3 | 8.8  (5.9–12.2) | 3 | 55.0  (6.4–98.4) |
| Employed (current or in the past) | 36 | 64.6  (59.9–69.1) | 29 | 67.9  (65.3–70.4) | 3 | 15.7  (5.0–30.3) | 3 | 39.8  (26.0–54.5) |
| Immigration status:  Born outside study region | 83 | 29.4  (24.6–34.3) | 75 | 22.1  (17.7–27.0) | 6 | 7.3  (1.7–15.7) | 7 | 30.9  (25.5–36.6) |

*Simple pooled estimates without random-effects model were calculated because of limited number of studies (<3).

CI, confidence interval; KP, key populations; MSM, men who have sex with men; NA, not available; PrEP, pre-exposure prophylaxis; PWID, people who inject drugs.

# **Supplementary Table 8a. Overview of Unique Sources Reporting HIV-1 Prevalence and/or PrEP Effectiveness Data, Including (n=145 sources)**

**Studies with ID numbers shaded grey were included in the assessment of PrEP effectiveness*

| **Author, Year** | **Data Year** | **Study Design** | **Country/**  **Region** | **Population Group** | **Individuals, N** | **HIV-1 Cases** | **Proportion of cases, %** | **Quality,^a^ %** |
| --- | --- | --- | --- | --- | --- | --- | --- | --- |
| Adu 2021^1^ | 1997-–2018 | Retrospective cohort | Canada | MSM | 1349 | 349 | 25.9 | 73 |
| Alain 2021^2^ | 2017–2018 | Cross-sectional | France | MSM | 9748 | 1142 | 11.7 | 45 |
| Altawalah 2019^3^ | 2017 | Cross-sectional | Kuwait | PWID | 521 | 4 | 0.8 | 73 |
| Barreiro Fernandez 2023^4^ | 2020–2022 | Retrospective cohort | Spain | PrEP users | 40 | 0 | 0 | 64 |
| Barrett 2019^5^ | 2015 | Cross-sectional | Ireland | MSM | 3090 | 152 | 4.9 | 45 |
| Blondeel 2021^6^ | 2011 | Cross-sectional | Portugal | MSM | 1046 | 95 | 9.1 | 73 |
| Bogdanic 2021^7^ | 2020–2021 | Cross-sectional | Croatia | MSM | 60 | 1 | 1.7 | 36 |
| Bowman 2021^8^ | 2016–2018 | Cross-sectional | Greece | MSM | 308 | 26 | 8.4 | 64 |
| Brogan 2019^9^ | 2017–2018 | Cross-sectional | Canada | MSM | 5165 | 464 | 9.0 | 45 |
| Brown 2023^10^ | 1988–2022 | National registry/database | UK | TGW & TGM | 6603 | 46 | 0.7 | 73 |
|  |  |  | UK | TGM | 3258 | 18 | 0.6 |  |
|  |  |  | UK | TGW | 3345 | 28 | 0.8 |  |
| Callander 2019^11^ | 2010–2017 | Cross-sectional | Australia | All KP | 79,368 | 5813 | 7.3 | 100 |
|  |  |  | Australia | MSM | 78,108 | 5756 | 7.4 |  |
|  |  |  | Australia | TGM | 404 | 14 | 3.5 |  |
|  |  |  | Australia | TGW | 492 | 28 | 5.7 |  |
| Callander 2023^12^ | 2010–2019 | Retrospective cohort | Australia | MSM | 101,772 | 11468 | 11.3 | 100 |
| Chan 2020^13^ | 2014–2018 | Cross-sectional | Australia | MSM | 7834 | 459 | 5.9 | 45 |
| Checkpoint Study^14^ | NR | Prospective cohort | Germany | PrEP users | 1756 | 1 | 0.1 | 73 |
| Chen 2021^15^ | 2013 | Cross-sectional | Taiwan | MSM | 2020 | 112 | 5.5 | 45 |
| Chiou 2022^16^ | 2017–2018 | Cross-sectional | Taiwan | MSM | 75 | 1 | 1.3 | 73 |
| Choi 2021^17^ | 2020 | Cross-sectional | Korea | PIP | 55,000 | 83 | 0.2 | 100 |
| Chuang 2021^18^ | 2017 | Cross-sectional | Taiwan | MSM | 1000 | 132 | 13.2 | 45 |
| Coyer 2022^19^ | 2008–2018 | Prospective cohort | Netherlands | All KP | 1115 | 55 | 5.6 | 55 |
|  |  |  | Netherlands | MSM | 976 | 55 | 5.6 |  |
|  |  |  | Netherlands | PrEP users | 139 | 0 | 0 |  |
| Coyer 2022^20^ | 2015–2019 | Prospective cohort | Netherlands | All KP | 398 | 6 | 1.5 | 82 |
|  |  |  | Netherlands | MSM | 199 | 6 | 3.0 |  |
|  |  |  | Netherlands | PrEP users | 199 | 0 | 0 |  |
| Croxford 2022^21^ | 1990–2019 | National registry/database | UK | PWID | 3139 | 26 | 0.8 | 100 |
| Daans 2023^22^ | 2000–2019 | Cross-sectional | Netherlands | TGW | 950 | 31 | 3.3 | 64 |
| Dagnä/NEPOS: Center Survey on PrEP Supply^14^ | NR | Prospective cohort | Germany | PrEP users | 4620 | 4 | 0.1 | 73 |
| Dagnä/NEPOS: PrEP Use in Priority Centers^14^ | NR | Prospective cohort | Germany | PrEP users | 22,366 | 20 | 0.1 | 73 |
| de la Court 2022^23^ | 2011 | Prospective cohort | Netherlands | MSM | 810 | 22 | 2.7 | 73 |
| de la Court 2023^24^ | 2015–2020 | Prospective cohort | Netherlands | PrEP users | 376 | 2 | 0.5 | 64 |
| de Sousa 2021^25^ | 2020 | Prospective cohort | Multi-country | All KP | 2361 | 249 | 10.5 | 45 |
|  |  |  | Portugal | MSM | 710 | 86 | 12.1 |  |
| Dharan 2023^26^ | 2016–2019 | Prospective cohort | Australia | PrEP users | 9596 | 30 | 0.3 | 73 |
| Drückler 2022^27^ | 2017–2018 | Retrospective cohort | Netherlands | All KP | 321 | 39 | 12.1 | 91 |
|  |  |  | Netherlands | TGW & SW | 137 | 20 | 14.6 |  |
|  |  |  | Netherlands | TGM | 54 | 0 | 0 |  |
|  |  |  | Netherlands | TGW | 267 | 39 | 14.6 |  |
| ECDC 2019^28^ | 2018 | Cross-sectional | Multi-country | MSM | 100,483 | 10,767 | 10.7 | 55 |
|  |  |  | Belgium | MSM | 3038 | 380 | 12.5 |  |
|  |  |  | Canada | MSM | 6059 | 551 | 9.1 |  |
|  |  |  | Croatia | MSM | 1015 | 51 | 5.0 |  |
|  |  |  | Denmark | MSM | 1698 | 229 | 13.5 |  |
|  |  |  | Estonia | MSM | 212 | 9 | 4.2 |  |
|  |  |  | Finland | MSM | 1409 | 86 | 6.1 |  |
|  |  |  | France | MSM | 10,996 | 1353 | 12.3 |  |
|  |  |  | Germany | MSM | 23,107 | 2472 | 10.7 |  |
|  |  |  | Iceland | MSM | 111 | 3 | 2.7 |  |
|  |  |  | Ireland | MSM | 2083 | 144 | 6.9 |  |
|  |  |  | Italy | MSM | 11,025 | 1114 | 10.1 |  |
|  |  |  | Netherlands | MSM | 3851 | 605 | 15.7 |  |
|  |  |  | Norway | MSM | 2957 | 145 | 4.9 |  |
|  |  |  | Portugal | MSM | 2555 | 365 | 14.3 |  |
|  |  |  | Spain | MSM | 10,652 | 1417 | 13.3 |  |
|  |  |  | Sweden | MSM | 4443 | 240 | 5.4 |  |
|  |  |  | Switzerland | MSM | 3383 | 355 | 10.5 |  |
|  |  |  | UK | MSM | 11,889 | 1248 | 10.5 |  |
| ECDC 2024^29^ | 2022 | NR | Bulgaria, Estonia | All KP | NR | NR | 0.3-13 | 18 |
| EMIS 2017 Ireland^30^ | 2017–2018 | National registry/database | Ireland | MSM | 2071 | 142 | 6.9 | 55 |
| EMIS 2017 Spain^31^ | 2017–2018 | Cross-sectional | Spain | MSM | 8533 | 1408 | 16.5 | 55 |
| Estcourt 2021^32^ | 2015–2019 | Retrospective cohort | UK | MSM | 18,608 | 89 | 0.5 | 82 |
|  |  |  | UK | MSM, no PrEP use | 9600 | 31 | 0.3 |  |
|  |  |  | UK | MSM & PrEP users | 2677 | 4 | 0.1 |  |
| Evers 2023^33^ | 2016–2021 | Retrospective cohort | Netherlands | MSM | 254,844 | 1235 | 0.5 | 82 |
| Fernandez-Lopez 2020^34^ | 1995–2019 | Cross-sectional | Spain | All KP | 71,979 | 2617 | 3.6 | 55 |
|  |  |  | Spain | MSM | 61,154 | 2050 | 3.4 |  |
|  |  |  | Spain | PWID | 2734 | 240 | 8.8 |  |
|  |  |  | Spain | SW | 8091 | 327 | 4.0 |  |
| Fernandez-Rollan 2019^35^ | 2016 | Cross-sectional | Chile | MSM | 246 | 42 | 17.1 | 64 |
| Ferrer 2022^36^ | 2017–2018 | Cross-sectional | Spain | SW | 146 | 37 | 25.3 | 64 |
| Fina 2019^37^ | 2017 | Retrospective cohort | UK | PrEP users | 296 | 0 | 0 | 100 |
| Fortier 2020^38^ | 2011–2016 | Cross-sectional | Canada | MSM | 386 | 28 | 7.3 | 64 |
| Gahrton 2019^39^ | 2017 | Cross-sectional | Sweden | PIP | 471 | 1 | 0.2 | 73 |
| Garcia-Pérez 2022^40^ | 2019–2020 | Cross-sectional | Spain | MSM | 497 | 123 | 24.7 | 64 |
| Garros 2021^41^ | 2019 | Cross-sectional | France | MSM | 21,762 | 1434 | 6.6 | 45 |
| Gasbarrini 2021^42^ | 2017–2020 | Prospective cohort | Croatia | MSM | 341 | 2 | 0.6 | 55 |
|  |  |  | Italy | All KP | 314 | 0 | 0 |  |
|  |  |  | Poland | PWID | 95 | 6 | 6.3 |  |
| Goddard 2020^43^ | 2010–2015 | Cross-sectional | Australia | MSM | 616 | 221 | 35.9 | 45 |
| Government of the Netherlands 2023^44^ | 2019–2022 | Prospective cohort | Netherlands | PrEP users | 12,195 | 44 | 0.4 | 73 |
| Gratrix 2019^45^ | 2012–2015 | Prospective cohort | Canada | PIP | 5053 | 14 | 0.3 | 73 |
| Greenwald 2019^46^ | 2013–2018 | Prospective cohort | Canada | PrEP users | 1551 | 0 | 0 | 73 |
| Grimshaw 2021^47^ | 2018–2020 | Retrospective cohort | UK | PrEP users | 32 | 0 | 0 | 73 |
| Grulich 2018^48^ | 2016 | Prospective cohort | Australia | PrEP users | 3638 | 2 | 0.1 | 91 |
| Grulich 2019^49^ | 2016–2018 | Prospective cohort | Australia | PrEP users | 9708 | 16 | 0.2 | 91 |
| Grulich 2021^50^ | 2016–2019 | Prospective cohort | Australia | PrEP users | 9566 | 30 | 0.3 | 82 |
| Guerras 2022^51^ | 2020 | Cross-sectional | Spain | MSM | 5492 | 768 | 14.0 | 55 |
| Hadjikou 2021^52^ | 2013–2015 | Retrospective cohort | Greece | PWID | 320 | 142 | 44.4 | 64 |
| Hanum 2019^53^ | 2015–2018 | Prospective cohort | UK | MSM | 622 | 13 | 2.1 | 45 |
| Hanum 2021^54^ | 2013–2018 | Prospective cohort | UK | MSM | 1162 | 33 | 2.8 | 64 |
|  |  |  | UK | PrEP users | 58 | 3 | 5.2 |  |
| Harney 2019^55^ | 2007–2014 | Cross-sectional | Australia | MSM | 8491 | 227 | 2.7 | 73 |
| Hart 2021^56^ | 2017–2019 | Cross-sectional | Canada | MSM | 2449 | 436 | 17.8 | 82 |
| HIV in Ireland: Latest trends to end 2022^57^ | 2019–2022 | National registry/database | Ireland | All KP | NR | 173 | NR | 100 |
| Holt 2019^58^ | 2011–2017 | Cross-sectional | Australia | MSM | 1121 | 83 | 7.4 | 45 |
| Holt 2022^59^ | 2021 | Cross-sectional | Australia | MSM | 1280 | 95 | 7.4 | 55 |
| Hoornenborg 2019^60^ | 2015–2018 | Prospective cohort | Netherlands | PrEP users | NR | NR | NR | 73 |
| Howarth 2022^61^ | 2020 | Cross-sectional | UK | MSM | 2018 | 203 | 10.1 | 45 |
| Hoyos-Mallecot 2022^62^ | 2016–2019 | Cross-sectional | Spain | All KP | 5361 | 43 | 0.8 | 45 |
|  |  |  | Spain | MSM | 5305 | 41 | 0.8 |  |
| Ikushima 2022^63^ | 2017–2021 | Cross-sectional | Japan | MSM | 4463 | 540 | 12.1 | 36 |
| Iniesta 2021^64^ | 2017–2019 | Prospective cohort | Spain | PrEP users | 321 | 0 | 0 | 73 |
| Izquierdo 2019^65^ | 2017 | Prospective cohort | France | PIP | 1093 | 14 | 1.3 | 55 |
| Jourdain 2022^66^ | 2016–2020 | Nested case control | France | PrEP users | NR | 256 | NR (adjusted odds ratio=0.40) | 64 |
| Kåberg 2020^67^ | 2013–2018 | Prospective cohort | Sweden | PWID | 2814 | 140 | 5.0 | 82 |
| Karlsson 2021^68^ | 2013 | Prospective cohort | Sweden | PWID | 2909 | 142 | 4.9 | 73 |
| Keane 2022^69^ | 2018–2020 | Cross-sectional | Ireland | All KP | NR | 111 | 2.9–3.8 | 91 |
| Keen 2020^70^ | 2010–2018 | Cross-sectional | Australia | All KP | NR | NR | 0.9–1.7 | 45 |
| King 2022^71^ | 2019–2021 | National registry/database | Australia | All KP | NR | NR | 0 | 100 |
| Kirby Institute 2022^72^ | NR | Cross-sectional | Australia | All KP | NR | NR | 1.5–9.2 | 27 |
| Kirby Institute 2023^73^ | 2018–2022 | Cross-sectional | Australia | PWID | 1955 | 65 | 3.3 | 64 |
|  |  |  | Australia | PWID & MSM | 119 | 24 | 20.2 |  |
|  |  |  | Australia | PWID & SW | 100 | 5 | 5.0 |  |
| Ku 2022 ^74^ | 2021 | Cross-sectional | Taiwan | MSM | 1880 | 152 | 8.1 | 36 |
| Kwan 2023^75^ | 2021 | Cross-sectional | Hong Kong | MSM | 337 | 2 | 0.6 | 64 |
| Lambert 2022^76^ | 2017–2021 | Prospective cohort | Canada | MSM | 2008 | 31 | 1.5 | 82 |
| Lapadula 2023^77^ | 2017 | Cross-sectional | Italy | TGW & SW | 78 | 25 | 32.1 | 91 |
| Lopez-Corbeto 2022^78^ | 2018 | Cross-sectional | Spain | SW | 400 | 12 | 3.0 | 64 |
| MacGibbon 2023^79^ | 2021 | Cross-sectional | Australia | MSM | 1280 | 95 | 7.4 | 55 |
| Marco 2019^80^ | 2002–2016 | Retrospective cohort | Spain | PIP | 602 | 173 | 28.7 | 55 |
| McAuley 2019^81^ | 2011–2018 | Cross-sectional | UK | PWID | 3528 | 73 | 2.1 | 73 |
| Meulbroek 2020^82^ | 2009–2017 | Prospective cohort | Spain | MSM | 25,916 | 809 | 3.1 | 64 |
| Mitchell 2019^83^ | 2014 | Cross-sectional | UK | MSM | 37,228 | 429 | 1.2 | 82 |
| Molina 2017^84^ | 2014–2016 | Prospective cohort | France, Canada | On-demand PrEP users | 361 | 1 | 0.3 | 82 |
| Molina 2019^85^ | 2017–2020 | Prospective cohort | France | On-demand & daily PrEP users | 3057 | 2 | 0.1 | 64 |
| Molina 2022^86^ | 2017–2020 | Prospective cohort | France | PrEP users | 3056 | 6 | 0.2 | 64 |
| Nelson 2019^87^ | 2011–2013 | Cross-sectional | Canada | MSM | 86 | 33 | 38.4 | 55 |
| Ogaz 2022^88^ | 2019 | Cross-sectional | UK | MSM | 1408 | 120 | 8.5 | 64 |
|  |  |  | UK | PrEP users | 153 | 3 | 2.0 |  |
| OHESI 2021^89^ | 2019 | National registry/database | Canada | MSM | 217,922 | 10,909 | 5.0 | 73 |
| Patel 2021^90^ | 2010–2018 | Retrospective cohort | Australia | All KP | NR | NR | 0.9-1.7 | 82 |
| Pavlopoulou 2020^91^ | 2012–2013 | Cross-sectional | Greece | PWID | 3320 | 506 | 15.2 | 64 |
| Pečavar 2021^92^ | 2018 | Prospective cohort | Slovenia | On-demand PrEP users | 69 | 0 | 0 | 64 |
| PHAC 2022^93^ | 2020 | National registry/database | Canada | All KP | NR | NR | NR | 100 |
| PHAC 2020^94^ | 2020 | National registry/database | Canada | All KP | NR | NR | NR | 100 |
| PHAS 2020^95^ | 2017–2018 | Cross-sectional | Sweden | MSM | 2913 | 210 | 7.2 | 64 |
| Prinsenberg 2022^96^ | 2018–2020 | cross-sectional | Netherlands | MSM | 50 | 15 | 30.0 | 27 |
| Rahib 2022^97^ | 2018 | Cross-sectional | France | MSM | 1556 | 15 | 1.0 | 73 |
| Richardson 2019^98^ | 2005–2015 | Cross-sectional | Canada | PWID | 1222 | 35 | 2.9 | 91 |
| Roussos 2022^99^ | 2014–2020 | Prospective cohort | Greece | PWID | 699 | 57 | 8.2 | 82 |
| Ryan 2019^100^ | 2016–2018 | Prospective cohort | Australia | People discontinued on PrEP | 130 | 4 | 3.1 | 46 |
| Ryan 2021^101^ | 2017–2018 | Prospective cohort | Spain | PWID | 297 | 36 | 12.2 | 82 |
|  |  |  | Spain | PWID & MSM | 9 | 2 | 22.2 |  |
|  |  |  | Spain | PWID & SW | 34 | 3 | 8.8 |  |
| Samji 2022^102^ | 2004–2017 | Prospective cohort | Canada | MSM | 9038 | 257 | 2.8 | 82 |
| Sang 2021^103^ | 2012–2015 | Prospective cohort | Canada | MSM | 762 | 213 | 28.0 | 64 |
| Santos 2021^104^ | 2020 | Cross-sectional | Multi-country | All KP | 2732 | 473 | 17.3 | 36 |
| Saxton 2020^105^ | 2011 | Cross-sectional | New Zealand | MSM | 3136 | 131 | 4.2 | 82 |
| Saxton 2022^106^ | 2019 | Cross-sectional | New Zealand | MSM | 36,635 | 58 | 0.2 | 91 |
| Scheim 2019^107^ | 2005–2014 | Prospective cohort | Canada | PWID | 1663 | 518 | 31.1 | 64 |
|  |  |  | Canada | PWID & MSM | 225 | 93 | 41.3 |  |
| Schmidt 2020^108^ | 2016–2017 | Prospective cohort | Switzerland | MSM | 779 | 28 | 3.6 | 73 |
| Schmidt 2023^109^ | 2019–2020 | Retrospective cohort | Germany | PrEP users | 4620 | 4 | 0.1 | 82 |
| Sekera 2022^110^ | 2003 | Cross-sectional | Czechia | PWID | 384 | 1 | 0.3 | 82 |
| Seviy 2022^111^ | 2022 | Cross-sectional | Croatia | PWID | 430 | 3 | 0.7 | 73 |
| Silvestri 2021^112^ | 2017–2018 | Cross-sectional | Italy | PIP | 474 | 9 | 1.9 | 100 |
| SIPrEP 2023^113^ | 2019–2023 | Prospective cohort | Spain | All KP | 2852 | 3 | 0.1 | 82 |
| SIPrEP 2021^114^ | 2020 | Prospective cohort | Spain | All KP | 832 | 3 | 0.4 | 82 |
| Skaletz-Rorowski 2022^115^ | 2017–2020 | Cross-sectional | Germany | MSM | 1317 | 12 | 0.9 | 55 |
| Slurink 2021^116^ | 2014–2017 | Retrospective cohort | Netherlands | MSM | 127,578 | 1057 | 0.8 | 82 |
| Steedman 2019^117^ | 2017–2018 | National registry/database | Scotland | PrEP users | 1846 | 2 | 0.1 | 100 |
| Stevens 2020^118^ | 2012–2018 | Cross-sectional | UK | MSM | 1682 | 788 | 46.8 | 55 |
| Stojanovski 2022^119^ | 2017–2018 | Cross-sectional | Multi-country | All KP | 98,600 | 13,059 | 13.2 | 55 |
| Streeck 2019^120^ | NR | Prospective cohort | Germany | MSM | 1000 | 5 | 0.5 | 45 |
| StuardoÁvila 2020^121^ | 2010 | Cross-sectional | Chile | MSM | 375 | 66 | 17.6 | 73 |
| Tarasuk 2020^122^ | 2019 | Cross-sectional | Canada | PWID | 879 | 135 | 15.4 | 64 |
| Tarasuk 2020^123^ | 2017–2020 | Cross-sectional | Canada | PWID | 2162 | 222 | 10.3 | 64 |
| Tarasuk 2020^124^ | 2019 | Cross-sectional | Canada | All KP | 1736 | 27 | 1.6 | 64 |
| Tarasuk 2021^125^ | 2019 | Cross-sectional | Canada | All KP | NR | NR | 10.3–15.4 | 64 |
| Tarasuk 2020^126^ | 2019 | Cross-sectional | Canada | All KP | NR | NR | 10.3–15.4 | 64 |
| Tassi 2021^127^ | 2016–2018 | Retrospective cohort | France | PrEP users | 9893 | 29 | 0.3 | 82 |
| Trayner 2020^128^ | 2017–2018 | Cross-sectional | UK | PWID | 1469 | 42 | 2.9 | 73 |
| UK HSA 2023^129^ | 2022 | Cross-sectional | UK | All KP | NR | NR | 1.2 | 64 |
| UNAIDS 2022^130^ | 2022 | National registry/database | Estonia | All KP | NR | NR | 5.5–51.4 | 36 |
| UNAIDS 2022^131^ | 2022 | NR | Multi-country | All KP | 523,700 | 6449 | 18.2 | 18 |
|  |  |  | Multi-country | PIP | 91,700 | 2993 | 3.3 |  |
|  |  |  | Multi-country | MSM | 432,000 | 3456 | 0.8 |  |
|  |  |  | Canada | PIP | 12,400 | 114 | 0.9 |  |
|  |  |  | Canada | MSM | 432,000 | 3456 | 0.8 |  |
|  |  |  | Estonia | PIP | 2300 | 9 | 0.4 |  |
|  |  |  | Portugal | PIP | 21,000 | 630 | 3.0 |  |
|  |  |  | Spain | PIP | 56,000 | 2240 | 4.0 |  |
| UNAIDS Atlas 2023^132^ | 2019–2022 | NR | Multi-country | All KP | NR | NR | 0–32.2 | 18 |
| vanBilsen 2020^133^ | 2009–2017 | Cross-sectional | Netherlands | MSM | 905 | 42 | 4.6 | 73 |
|  |  |  | Netherlands | PrEP users | 69 | 0 | 0 |  |
| Van Meer 2019^134^ | 2010 | Retrospective cohort | Canada | PIP | 52,313 | 363 | 0.7 | 100 |
| van Santen 2021^135^ | 1985–2014 | Prospective cohort | Netherlands | PWID | 640 | 59 | 9.2 | 91 |
| Velter 2017^136^ | 2015 | Cross-sectional | France | MSM | 2646 | 433 | 16.4 | 73 |
| Velter 2017^137^ | 2015 | Cross-sectional | France | MSM | 2646 | 366 | 13.8 | 73 |
| Velter 2019^138^ | 2017–2019 | Cross-sectional | France | MSM | 36,002 | 2497 | 6.9 | 36 |
| Velter 2022^139^ | 2019 | Cross-sectional | France | MSM | 7303 | 707 | 9.7 | 45 |
| Vernazza 2020^140^ | 2016–2017 | Prospective cohort | Switzerland | Female SW | 490 | 2 | 0.4 | 73 |
|  |  |  | Switzerland | TGW & SW | 17 | 1 | 5.9 |  |
| Vu 2020^141^ | 2015–2016 | Cross-sectional | Switzerland | SW | 96 | 0 | 0 | 64 |
| Vuylsteke 2019^142^ | 2017–2018 | Prospective cohort | Belgium | PrEP users | 200 | 0 | 0 | 64 |
|  |  |  | Belgium | Daily PrEP users | 153 | 0 | 0 |  |
| Wayal 2019^143^ | 2017 | Cross-sectional | UK | MSM | 3663 | 489 | 13.3 | 55 |
| Wiessing 2021^144^ | 2006–2020 | Cross-sectional | Multi-country | PWID | 52,368 | 2853 | 5.4 | 55 |
|  |  |  | Austria | PWID | 608 | 3 | 0.5 |  |
|  |  |  | Belgium | PWID | 363 | 14 | 3.9 |  |
|  |  |  | Croatia | PWID | 397 | 0 | 0 |  |
|  |  |  | Cyprus | PWID | 888 | 3 | 0.3 |  |
|  |  |  | Estonia | PWID | 1277 | 729 | 57.1 |  |
|  |  |  | France | PWID | 898 | 122 | 13.6 |  |
|  |  |  | Germany | PWID | 2069 | 101 | 4.9 |  |
|  |  |  | Greece | PWID | 934 | 29 | 3.1 |  |
|  |  |  | Hungary | PWID | 1180 | 3 | 0.3 |  |
|  |  |  | Latvia | PWID | 3047 | 680 | 22.3 |  |
|  |  |  | Lithuania | PWID | 530 | 57 | 10.8 |  |
|  |  |  | Poland | PWID | 181 | 34 | 18.8 |  |
|  |  |  | Portugal | PWID | 1901 | 365 | 19.2 |  |
|  |  |  | Romania | PWID | 522 | 151 | 28.9 |  |
|  |  |  | Sweden | PWID | 8512 | 214 | 2.5 |  |
|  |  |  | UK | PWID | 29,061 | 348 | 1.2 |  |
| Wong 2020^145^ | 2017 | Cross-sectional | Hong Kong | MSM | 3044 | 173 | 5.7 | 64 |

*Studies with ID numbers shaded grey were included in the assessment of PrEP effectiveness.

^a^ Quality was assessed using the Joanna Briggs Institute critical appraisal tool.

KP, key populations; MSM, men who have sex with men; NR, not reported; PIP, people in prison; PrEP, pre-exposure prophylaxis; PWID, people who inject drugs; PY, person-years; SW, sex workers; TGM, transgender men; TGW, transgender women.

# **Supplementary Table 8b. Overview of Included Sources With HIV-1 Incidence Data^a^ (n=26 sources)**

**Studies with ID numbers shaded grey were included in the assessment of PrEP effectiveness*

| **Author, Year** | **Data Year** | **Study Design** | **Country/ Region** | **Population Group** | **Total Person Time, Years** | **HIV-1 Cases** | **Incidence Rate, per 100 PY** | **Quality,^a^ %** |
| --- | --- | --- | --- | --- | --- | --- | --- | --- |
| Callander 2023^12^ | 2010–2019 | Retrospective cohort | Australia | MSM | 301,313 | 1201 | 0.4 | 100 |
| Checkpoint Study^14^ | NR | Prospective cohort | Germany | PrEP users | NR | NR | 0.06 | 73 |
| Dagnä/NEPOS: Center Survey on PrEP Supply^14^ | NR | Prospective cohort | Germany | PrEP users | 5128 | 4 | 0.1 | 73 |
| Estcourt 2021^32^ | 2015–2019 | Retrospective cohort | UK | MSM | 21,296 | 89 | 0.4 | 82 |
|  |  |  | UK | MSM, no PrEP use | 10,524 | 54 | 0.5 |  |
|  |  |  | UK | MSM & PrEP users | 10,772 | 35 | 0.3 |  |
| Grulich 2018^48^ | 2016 | Prospective cohort | Australia | PrEP users | 4100 | 2 | 0.05 | 91 |
| Grulich 2019^49^ | 2016–2018 | Prospective cohort | Australia | PrEP users | 17,747 | 16 | 0.1 | 91 |
| Grulich 2021^50^ | 2016–2019 | Prospective cohort | Australia | PrEP users | 18,634 | 30 | 0.2 | 82 |
| Hanum 2019^53^ | 2015–2018 | Prospective cohort | UK | MSM | 1044 | 13 | 1.2 | 45 |
| Hanum 2021^54^ | 2013–2018 | Prospective cohort | UK | MSM | 4618.86 | 33 | 0.7 | 64 |
| Hoornenborg 2019^60^ | 2015–2018 | Prospective cohort | Netherlands | PrEP users | 673.4 | 2 | 0.3 | 73 |
| Kirby Institute 2022^72^ | NR | Cross-sectional | Australia | All KP | NR | NR | 0.6 | 27 |
| Lambert 2022^76^ | 2017–2021 | Prospective cohort | Canada | MSM | 7373 | 29 | 0.4 | 82 |
| Meulbroek 2020^82^ | 2009–2017 | Prospective cohort | Spain | MSM | 23,980 | 506 | 2.1 | 64 |
| Mitchell 2019^83^ | 2014 | Cross-sectional | UK | MSM | 24,709 | 445 | 1.8 | 82 |
| Molina 2022^86^ | 2017–2020 | Prospective cohort | France | PrEP users | 5623 | 6 | 0.1 | 64 |
| PHAC 2022^93^ | 2020 | National registry/database | Canada | All KP | NR | NR | 0.17–0.24 | 100 |
| PHAC 2020^94^ | 2020 | National registry/database | Canada | All KP | NR | NR | 0.17–0.24 | 100 |
| Roussos 2022^99^ | 2014–2020 | Prospective cohort | Greece | PWID | 2932 | 57 | 1.9 | 82 |
| Samji 2022^102^ | 2004–2017 | Prospective cohort | Canada | MSM | 30,241.8 | 257 | 0.8 | 82 |
| Schmidt 2020^108^ | 2016–2017 | Prospective cohort | Switzerland | MSM | 333.2 | 1 | 0.3 | 73 |
| Schmidt 2023^109^ | 2019–2020 | Retrospective cohort | Germany | PrEP users | 5128 | 4 | 0.1 | 82 |
| SIPrEP 2023^113^ | 2019–2023 | Prospective cohort | Spain | All KP | 1800 | 3 | 0.2 | 82 |
| SIPrEP 2021^114^ | 2020 | Prospective cohort | Spain | All KP | 214 | 3 | 1.4 | 82 |
| Steedman 2019 ^117^ | 2017–2018 | National registry/database | Scotland | PrEP users | 71,667 | 215 | 0.3 | 100 |
| Tassi 2021 ^127^ | 2016–2018 | Retrospective cohort | France | PrEP users | 15,263 | 29 | 0.2 | 82 |
| van Bilsen 2020 ^133^ | 2009–2017 | Cross-sectional | Netherlands | MSM | 4940 | 42 | 0.9 | 73 |
| Vuylsteke 2019 ^142^ | 2017–2018 | Prospective cohort | Belgium | PrEP users | 318 | 0 | 0 | 64 |

*Studies with ID numbers shaded grey were included in the assessment of PrEP effectiveness.

^a^ Quality was assessed using the Joanna Briggs Institute critical appraisal tool.

KP, key populations; MSM, men who have sex with men; NR, not reported; PIP, people in prison; PrEP, pre-exposure prophylaxis; PWID, people who inject drugs.

# **Supplementary Table 8c. Overview of Included Sources With PrEP Use Data (n=88 sources)**

| **Author, Year** | **Data Years** | **Study Design** | **Country/ Region** | **Population Group** | **Individuals, N** | **PrEP users, N** | **Proportion of PrEP users, %** | **Quality,^a^ %** |
| --- | --- | --- | --- | --- | --- | --- | --- | --- |
| Alain 20212^2^ | 2017–2018 | Cross-sectional | France | MSM | 8527 | 779 | 9.1 | 45 |
| Annequin 2020^146^ | 2017–2018 | Cross-sectional | France | MSM | 7965 | 734 | 9.2 | 55 |
| Basten 2021^147^ | 2017–2018 | Prospective cohort | Netherlands | MSM | 754 | 77 | 10.2 | 45 |
| Bavinton 2019^148^ | 2012–2016 | Prospective cohort | Australia | MSM & serodiscordant couples | 153 | 41 | 26.8 | 27 |
| Bavinton 2021^149^ | 2014–2017 | Prospective cohort | Australia | MSM | 1386 | 347 | 25.0 | 36 |
| Bayley 2018^150^ | 2018 | Cross-sectional | UK | MSM | 201 | NR | 34.7 | 55 |
| Berghe 2021^151^ | 2017–2018 | Cross-sectional | Belgium | MSM | 2722 | 101 | 3.7 | 55 |
| Bogdanic 2021^7^ | 2020–2021 | Cross-sectional | Croatia | MSM | 81 | 0 | 0 | 36 |
| Bourne 2019^152^ | 2017 | Cross-sectional | France | MSM | 2544 | 281 | 11.0 | 55 |
|  |  |  | Russia | MSM | 2393 | 195 | 8.1 |  |
|  |  |  | UK | MSM | 1414 | 146 | 10.3 |  |
|  |  |  | Italy | MSM | 797 | 50 | 6.3 |  |
|  |  |  | Ukraine | MSM | 374 | 62 | 16.6 |  |
|  |  |  | Germany | MSM | 309 | 30 | 9.7 |  |
|  |  |  | Belgium | MSM | 209 | 19 | 9.1 |  |
|  |  |  | Spain | MSM | 183 | 13 | 7.1 |  |
|  |  |  | Netherlands | MSM | 158 | 21 | 13.3 |  |
|  |  |  | Denmark | MSM | 145 | 14 | 9.7 |  |
|  |  |  | Switzerland | MSM | 134 | 8 | 6.0 |  |
|  |  |  | Portugal | MSM | 131 | 6 | 4.6 |  |
|  |  |  | Sweden | MSM | 117 | 18 | 15.4 |  |
| BRAHMS Study^14^ | NR | Prospective cohort | Germany | MSM | 1017 | NR | 0.5 | 73 |
| Brogan 2019^9^ | 2017–2018 | Cross-sectional | Canada | MSM | 5165 | 431 | 8.3 | 45 |
| Buffel 2022^153^ | 2017–2018 | Cross-sectional | Belgium | MSM | 2376 | 164 | 6.9 | 55 |
| Bull 2018^154^ | 2015–2016 | Cross-sectional | UK | MSM | 724 | 59 | 8.1 | 45 |
| Callander 2019^155^ | 2016 | Cross-sectional | France | MSM | 444 | NR | 10.4 | 55 |
| Callander 2019^156^ | 2018 | Cross-sectional | Australia | TGM & TGW | 1613 | 36 | 2.2 | 45 |
| Callander 2023^12^ | 2016–2019 | Prospective cohort | Australia | MSM | 34,744 | 5951 | 17.1 | 73 |
| Chan 2020^157^ | 2019–2020 | Cross-sectional | Australia | MSM | 951 | 276 | 29.0 | 55 |
| Chan 2023^158^ | 2022 | Cross-sectional | Multi-country | TGW | 980 | 461 | 47.0 | 64 |
|  |  |  | Multi-country | MSM | 4120 | 790 | 19.2 |  |
|  |  |  | Australia | MSM | 1617 | 751 | 46.4 |  |
| Checkpoint Study^14^ | 2021 | Cross-sectional | Germany | Individuals seeking PrEP | 47,186 | 5285 | 11.2 | 73 |
| Colyer 2021^159^ | 2017–2018 | Cross-sectional | Canada | MSM | 4926 | 429 | 8.7 | 55 |
| Coyer 2018^160^ | 2015–2017 | Cross-sectional | Netherlands | MSM | 687 | 66 | 9.6 | 45 |
| de Sousa 2021^25^ | 2020 | Cross-sectional | Portugal | MSM | 710 | 110 | 15.5 | 45 |
| Di Ciaccio 2022^161^ | 2020 | Cross-sectional | France | MSM | 556 | 427 | 76.8 | 27 |
| Duchesne 2020^162^ | 2019 | Cross-sectional | France | MSM | 7334 | NR | 14.0 | 45 |
| ECDC 2019^28^ | 2017–2018 | Cross-sectional | Finland | MSM | NR | NR | 0.5 | 55 |
|  |  |  | Iceland | MSM | NR | NR | 4.6 |  |
|  |  |  | Norway | MSM | NR | NR | 5.4 |  |
|  |  |  | Belgium | MSM | NR | NR | 6.8 |  |
|  |  |  | Croatia | MSM | NR | NR | 0.3 |  |
|  |  |  | Denmark | MSM | NR | NR | 3.9 |  |
|  |  |  | Estonia | MSM | NR | NR | 0.5 |  |
|  |  |  | France | MSM | NR | NR | 8.4 |  |
|  |  |  | Germany | MSM | NR | NR | 2.1 |  |
|  |  |  | Ireland | MSM | NR | NR | 4.0 |  |
|  |  |  | Italy | MSM | NR | NR | 0.4 |  |
|  |  |  | Netherlands | MSM | NR | NR | 4.5 |  |
|  |  |  | Portugal | MSM | NR | NR | 1.5 |  |
|  |  |  | Spain | MSM | NR | NR | 1.8 |  |
|  |  |  | Sweden | MSM | NR | NR | 0.8 |  |
|  |  |  | UK | MSM | NR | NR | 8.6 |  |
|  |  |  | Switzerland | MSM | NR | NR | 4.0 |  |
|  |  |  | Canada | MSM | NR | NR | 7.8 |  |
| ECDC 2024^163^ | 2022 | NR | France | MSM | NR | 47071 | 22.0 | 27 |
| EMIS 2017 Ireland^30^ | 2017–2018 | National registry/database | Ireland | MSM | 1917 | 11 | 0.6 | 55 |
| EMIS 2017 Spain^31^ | 2017–2018 | Cross-sectional | Spain | MSM | NR | 167 | 1.8 | 55 |
| Estcourt 2021^32^ | 2017–2019 | Retrospective cohort | UK | MSM | 16,723 | 3256 | 19.5 | 64 |
| Evers 2023^33^ | 2019–2021 | Retrospective cohort | Netherlands | MSM | 21,665 | 7800 | 36 | 73 |
| Fina 2019^37^ | 2017 | Prospective cohort | UK | Eligible for PrEP | 516 | 296 | 57.4 | 91 |
| Fraser 2020^164^ | 2018–2019 | Cross-sectional | Australia | Individuals offered PrEP | 2344 | 2051 | 87.5 | 64 |
| Gaskins 2021^165^ | 2017–2018 | Cross-sectional | Germany | MSM | 228 | 65 | 28.5 | 45 |
| Government of the Netherlands 2023^44^ | 2019–2022 | Prospective cohort | Netherlands | MSM | 12,195 | 4678 | 38.4 | 73 |
| Greenwald 2019^46^ | 2013–2018 | Prospective cohort | Canada | Eligible for PrEP | 2156 | 1551 | 71.9 | 36 |
| Grimshaw 2021^47^ | 2018–2020 | Retrospective cohort | UK | Eligible for PrEP | 41 | 32 | 78.0 | 73 |
| Hambrick 2018^166^ | 2016 | Cross-sectional | France | MSM | 444 | 14 | 3.2 | 55 |
| Hammoud 2019^167^ | 2014–2015 | Prospective cohort | Australia | MSM | 1257 | 226 | 18.0 | 45 |
| Hampel 2017^168^ | 2017 | Cross-sectional | Switzerland | MSM | 1893 | 82 | 4.3 | 55 |
| Hanum 2020^169^ | 2013–2018 | Prospective cohort | UK | MSM | NR | NR | 0-43.4 | 36 |
| Hanum 2020^170^ | 2013–2018 | Prospective cohort | UK | MSM | 1159 | 58 | 5.0 | 36 |
| Hanum 2021^54^ | 2013–2019 | Prospective cohort | UK | MSM | 1159 | 58 | 5.0 | 45 |
| Hart 2021^56^ | 2017–2018 | Cross-sectional | Canada | MSM | NR | NR | 9.6–18.9 | 55 |
| HIV Monitoring Report 2023^171^ | 2018–2023 | National registry/database | Netherlands | MSM | 676 | 97 | 14.3 | 73 |
| HIV testing, PrEP, new HIV diagnoses and care outcomes for people accessing HIV services: 2023 report^129^ | 2022 | National registry/database | UK | Eligible for PrEP | 121,547 | 86324 | 71.0 | 82 |
| HIVLens 2024^172^ | 2015–2022 | National registry/database | UK | Eligible for PrEP | 88,216 | 61510 | 69.7 | 45 |
| Holt 2017^173^ | 2015 | Cross-sectional | Australia | MSM | 1251 | 38 | 3.0 | 55 |
| Holt 2018^174^ | 2016 | Cross-sectional | Australia | MSM | 771 | 97 | 12.6 | 55 |
| Holt 2019^58^ | 2017 | Cross-sectional | Australia | MSM | 1121 | 265 | 23.6 | 55 |
| Holt 2020^175^ | 2018 | Cross-sectional | Australia | MSM | 7878 | 1155 | 14.7 | 64 |
| Hovaguimian 2022^176^ | 2019–2020 | Prospective cohort | Switzerland | Individuals seeking PrEP | 910 | 528 | 58.0 | 27 |
| HPSC 2022^57^ | NR | NR | Ireland | Individuals registered in the national PrEP programme | 2024 | 1763 | 87.1 | 36 |
| Ikushima 2022^63^ | 2021 | Cross-sectional | Japan | MSM | 5690 | 159 | 2.8 | 36 |
| King 2022^71^ | 2017–2021 | National registry/database | Australia | General population | NR | NR | 0.3–0.6 | 36 |
| Kirby Institute 2022^72^ | NR | Cross-sectional | Australia | General population | NR | NR | 10.6–21.3 | 27 |
| Krist 2022^177^ | 2017–2019 | Cross-sectional | Netherlands | MSM | 757 | 144 | 19.0 | 45 |
| Lachowsky 2019^178^ | 2017 | Cross-sectional | Canada | MSM | 232 | 41 | 17.7 | 36 |
| Leobon 2022^179^ | NR | Cross-sectional | France | MSM | 3251 | 445 | 13.7 | 45 |
| Logan 2019^180^ | 2016 | Cross-sectional | UK | MSM | 744 | 46 | 6.2 | 55 |
| MacGibbon 2021^181^ | 2019 | Cross-sectional | Australia | MSM | 1141 | 439 | 38.5 | 55 |
| Meireles 2021^182^ | 2014–2019 | Cross-sectional | Portugal | MSM | 6164 | 198 | 3.2 | 55 |
| Molina 2022^86^ | 2017–2019 | Prospective cohort | France | Eligible for PrEP | 3056 | 1712 | 56.0 | 45 |
| Mosley 2018^183^ | 2012–2016 | Prospective cohort | Canada | MSM | 528 | 8 | 1.5 | 55 |
| Murray 2020^184^ | 2015–2016 | Cross-sectional | Australia | MSM | 567 | 23 | 4.1 | 55 |
| Nguyen 2020^185^ | 2022 | Cross-sectional | Sweden | MSM | 46 | NR | 0.2 | 45 |
| O'Byrne 2021^186^ | 2018–2020 | Prospective cohort | Canada | Eligible for PrEP | 347 | 129 | 37.2 | 55 |
| Ogaz 2022^88^ | 2019 | Cross-sectional | UK | MSM | 1224 | 270 | 22.1 | 55 |
| O'Halloran 2019^187^ | 2019 | Cross-sectional | UK | Individuals seeking PrEP | 2275 | 1742 | 76.6 | 45 |
| PHAS 2020^95^ | 2017–2018 | Cross-sectional | Sweden | MSM | 4008 | NR | 0.03 | 64 |
| Pico-Espinosa 2023^188^ | 2019–2020 | Cross-sectional | Canada | MSM | 1527 | 933 | 61.1 | 36 |
| Sang 2022^189^ | 2017–2020 | Prospective cohort | Canada | MSM | 2008 | 398 | 19.8 | 45 |
| Sauvage 2018^190^ | 2015 | Cross-sectional | France | MSM | NR | 67 | 2.3 | 55 |
| Saxby 2022^191^ | 2015–2019 | Cross-sectional | Australia | MSM | 43,781 | NR | 16.2 | 55 |
| Spinner 2018^192^ | 2016 | Cross-sectional | Germany | MSM | 866 | 65 | 7.5 | 55 |
| Streeck 2022^193^ | 2018–2019 | Prospective cohort | Germany | MSM | 1043 | 553 | 53.0 | 64 |
| Suen 2022^194^ | 2019–2020 | Cross-sectional | Hong Kong | MSM | 279 | NR | 2.4 | 55 |
| Tan 2018^195^ | 2014–2018 | Cross-sectional | Singapore | MSM | 1025 | 154 | 15.0 | 55 |
| CCDR 2020^124^  Tarasuk 2020^126^ | 2017–2020 | Cross-sectional | Canada | PWID | NR | NR | 0.3 | 45 |
| Tooley 2021^196^ | NR | Cross-sectional | Canada | MSM | 3290 | NR | 13.0 | 55 |
| UK HSA 2023^197^ | 2021–2022 | National registry/database | UK | MSM | 98,565 | 72,457 | 73.5 | 82 |
| van Dijk 2020^198^ | 2016 | Cross-sectional | Netherlands | MSM | 426 | 29 | 6.8 | 55 |
| van Dijk 2021^199^ | 2017–2019 | Cross-sectional | Netherlands | MSM | 767 | 144 | 18.8 | 45 |
| Velter 2019^138^ | 2019 | Cross-sectional | France | MSM | 19,022 | 1433 | 7.5 | 36 |
| Velter 2022^200^ | 2021 | Cross-sectional | France | MSM | 3259 | NR | 28.2 | 55 |
| Walmsley 2019^201^ | 2017–2018 | Prospective cohort | Canada | MSM | 141 | 37 | 26.2 | 36 |
| Wang 2018^202^ | 2018 | Cross-sectional | Hong Kong | MSM | 403 | 4 | 1.0 | 64 |
| Wang 2020^203^ | 2017–2018 | Cross-sectional | Canada | MSM | 831 | 112 | 13.5 | 55 |
| Wang 2022^204^ | 2017–2018 | Cross-sectional | Netherlands | MSM | 3232 | NR | 3.1–7.9 | 55 |

^a^ Quality was assessed using the Joanna Briggs Institute critical appraisal tool.

KP, key populations; MSM, men who have sex with men; NR, not reported; PrEP, pre-exposure prophylaxis; PWID, people who inject drugs; TGM, transgender men; TGW, transgender women.

# **Supplemental Table 9. I^2^ Statistics for Heterogeneity Among Included Sources for HIV-1 Epidemiology, PrEP Effectiveness, and PrEP Use, by Country/Region and Key Population**

| **Country/ region** | **HIV-1 prevalence** | | | | | | | | **HIV-1 IR** | | **PrEP effectiveness** | | **PrEP Use** | | | |
| --- | --- | --- | --- | --- | --- | --- | --- | --- | --- | --- | --- | --- | --- | --- | --- | --- |
|  | All KP | MSM | PWID | PIP | SW | TGW | TGM | MKP | All KP | MSM | Prev., all KP | IR,  all KP | All KP | MSM | TGW | TGM |
| Overall | 99.88 | 99.90 | 99.20 | 99.81 | 95.42 | 98.23 | 89.53 | 92.33 | 98.85 | 99.39 | 71.91 | 65.34 | 99.97 | 99.97 | 99.87 | 99.75 |
| Australia | 99.87 | 99.59 | NA |  | NA | NA | NA | NA | 95.51 | NA | 78.88 | 62.93 | 99.84 | 99.33 | NA | NA |
| Austria | NA |  | NA |  |  |  |  |  |  |  |  |  |  |  |  |  |
| Belgium | 97.73 | NA | NA |  |  |  |  |  | NA |  | NA | NA | 93.68 | 93.68 |  |  |
| Canada | 99.90 | 99.93 | 99.52 | 92.35 |  |  |  | NA | 93.53 | 93.53 | NA |  | 99.81 | 99.62 |  |  |
| Croatia | 93.36 | 90.43 | 71.31 |  |  |  |  |  |  |  |  |  | NA | NA |  |  |
| Cyprus | NA |  | NA |  |  |  |  |  |  |  |  |  |  |  |  |  |
| Czechia | NA |  | NA |  |  |  |  |  |  |  |  |  |  |  |  |  |
| Denmark | NA | NA |  |  |  |  |  |  |  |  |  |  | NA | NA |  |  |
| Estonia | 99.90 | NA | NA | NA |  |  |  |  |  |  |  |  |  |  |  |  |
| Finland | NA | NA |  |  |  |  |  |  |  |  |  |  |  |  |  |  |
| France | 99.71 | 99.30 | NA | NA |  |  |  |  | 39.58 |  | 0.00 | 39.58 | 99.87 | 99.85 |  |  |
| Germany | 99.86 | 99.61 | NA |  |  |  |  |  | 0.00 |  | 0.00 | 0.00 | 99.61 | 99.60 | NA | NA |
| Greece | 98.82 | NA | 99.10 |  |  |  |  |  | NA |  |  |  |  |  |  |  |
| Hong Kong | 96.59 | 96.59 |  |  |  |  |  |  |  |  |  |  | 56.12 | 56.12 |  |  |
| Hungary | NA |  | NA |  |  |  |  |  |  |  |  |  |  |  |  |  |
| Iceland | NA | NA |  |  |  |  |  |  |  |  |  |  |  |  |  |  |
| Ireland | 83.95 | 83.95 |  |  |  |  |  |  |  |  |  |  | NA | NA |  |  |
| Italy | 98.41 | NA |  | NA | NA | NA |  | NA |  |  |  |  | NA | NA |  |  |
| Japan | NA | NA |  |  |  |  |  |  |  |  |  |  | NA | NA |  |  |
| Korea | NA |  |  | NA |  |  |  |  |  |  |  |  |  |  |  |  |
| Kuwait | NA |  | NA |  |  |  |  |  |  |  |  |  |  |  |  |  |
| Latvia | NA |  | NA |  |  |  |  |  |  |  |  |  |  |  |  |  |
| Lithuania | NA |  | NA |  |  |  |  |  |  |  |  |  |  |  |  |  |
| Netherlands | 99.50 | 99.66 | NA |  | NA | 98.01 | NA | NA | 52.65 | NA | 0.00 | NA | 99.71 | 99.71 |  |  |
| New Zealand | 99.69 | 99.69 |  |  |  |  |  |  |  |  |  |  |  |  |  |  |
| Norway | NA | NA |  |  |  |  |  |  |  |  |  |  |  |  |  |  |
| Poland | 88.62 |  | 88.62 |  |  |  |  |  |  |  |  |  |  |  |  |  |
| Portugal | 99.58 | 89.81 | NA | NA |  |  |  |  |  |  |  |  | 98.45 | 98.45 |  |  |
| Romania | NA |  | NA |  |  |  |  |  |  |  |  |  |  |  |  |  |
| Russia |  |  |  |  |  |  |  |  |  |  |  |  | NA | NA |  |  |
| Singapore |  |  |  |  |  |  |  |  |  |  |  |  | NA | NA |  |  |
| Spain | 99.69 | 99.82 | 70.78 | 99.68 | 95.37 |  |  | NA | 89.84 | NA | 0.00 |  | 92.86 | 92.86 |  |  |
| Sweden | 97.36 | 89.81 | 96.52 | NA |  |  |  |  |  |  |  |  | 97.11 | 97.11 |  |  |
| Switzerland | 98.09 | 97.92 |  |  | 0.00 |  |  | NA | NA | NA |  |  | 99.81 | 0.00 |  |  |
| Taiwan | 94.52 | 94.52 |  |  |  |  |  |  |  |  |  |  |  |  |  |  |
| Ukraine |  |  |  |  |  |  |  |  |  |  |  |  | NA | NA |  |  |
| UK | 99.74 | 99.85 | 92.61 |  |  | NA | NA |  | 99.36 | 99.35 | 71.47 | 86.85 | 99.96 | 99.97 |  |  |

IR, incidence rate; KP, key populations; MKP, individuals in multiple key populations; MSM, men who have sex with men; NA, not applicable; PIP, people in prison; PrEP, pre-exposure prophylaxis; Prev., prevalence; PWID, people who inject drugs; SW, sex workers; TGM, transgender men; TGW, transgender women.

# **References**

1. Adu PA, Rossi C, Binka M, et al. HCV reinfection rates after cure or spontaneous clearance among HIV-infected and uninfected men who have sex with men. *Liver Int.* 2021;41(3):482-493.

2. Alain T, Villes V, Morel S, et al. European MSM Internet Survey (EMIS-2017). France national report. 2022; https://www.santepubliquefrance.fr/maladies-et-traumatismes/infections-sexuellement-transmissibles/vih-sida/documents/enquetes-etudes/european-msm-internet-survey-emis-2017-.-rapport-national-de-la-france. Accessed 11 September 2024.

3. Altawalah H, Essa S, Ezzikouri S, Al-Nakib W. Hepatitis B virus, hepatitis C virus and human immunodeficiency virus infections among people who inject drugs in Kuwait: A cross-sectional study. *Sci Rep.* 2019;9(1):6292.

4. Barreiro Fernandez EM, Dominguez Santana CM, Rios Sanchez E, Blanco Castaño MA, Salmeron Navas FJ. Preexposure Prophylaxis in Men at High Risk For Hiv-1 Infection [Abstract 4CPS-125]. *Eur J Hosp Pharm.* 2023;30:A1–A239.

5. Barrett P, O'Donnell K, Fitzgerald M, et al. Drug use among men who have sex with men in Ireland: Prevalence and associated factors from a national online survey. *Int J Drug Policy.* 2019;64:5-12.

6. Blondeel K, Dias S, Furegato M, et al. Sexual behaviour patterns and STI risk: results of a cluster analysis among men who have sex with men in Portugal. *BMJ Open.* 2021;11(1):e033290.

7. Bogdanic N, Dubravic D, Kresovic J, et al. PO3/03 Preliminary results of a pilot project on HIV self-testing among men who have sex with men in the city of Zagreb, Croatia. 2021; https://region-hovedstaden-ekstern.23video.com/po3-3-bogdanicmp4mp4. Accessed 11 September 2024.

8. Bowman B, Psichogyiou M, Papadopoulou M, et al. Sexual Mixing and HIV Transmission Potential Among Greek Men Who have Sex with Men: Results from SOPHOCLES. *AIDS Behav.* 2021;25(6):1935-1945.

9. Brogan N, Paquette DM, Lachowsky NJ, et al. Canadian results from the European Men-who-have-sex-with-men Internet survey (EMIS-2017). *Can Commun Dis Rep.* 2019;45(11):271-282.

10. Brown J, Pfeiffer RM, Shrewsbury D, et al. Prevalence of cancer risk factors among transgender and gender diverse individuals: a cross-sectional analysis using UK primary care data. *Br J Gen Pract.* 2023;73(732):e486-e492.

11. Callander D, Cook T, Read P, et al. Sexually transmissible infections among transgender men and women attending Australian sexual health clinics. *Med J Aust.* 2019;211(9):406-411.

12. Callander D, McManus H, Gray RT, et al. HIV treatment-as-prevention and its effect on incidence of HIV among cisgender gay, bisexual, and other men who have sex with men in Australia: a 10-year longitudinal cohort study. *Lancet HIV.* 2023;10(6):e385-e393.

13. Chan C, Broady TR, Bavinton BR, Mao L, Prestage GP, Holt M. Assessing the HIV Prevention Needs of Young Gay and Bisexual Men in the PrEP Era: An Analysis of Trends in Australian Behavioural Surveillance, 2014-2018. *AIDS Behav.* 2020;24(8):2382-2386.

14. Robert Koch Institut. Evaluation of the introduction of HIV pre-exposure prophylaxis as a statutory health insurance benefit (EvE-PrEP). 2023; https://www.rki.de/DE/Content/InfAZ/H/HIVAIDS/Abschlussbericht_EvE-PrEP.pdf?__blob=publicationFile. Accessed 11 September 2024.

15. Chen LY, Wu SC, Ko NY. Moderating effect of illicit drug use on the relationship between sexual behaviours and prevalence of HIV or sexually transmitted infections. *Drug Alcohol Rev.* 2021;40(2):287-295.

16. Chiou PY, Hung CC, Chen CY. Sexual Partner Referral for HIV Testing Through Social Networking Platforms: Cross-sectional Study. *JMIR Public Health Surveill.* 2022;8(4):e32156.

17. Choi S, Lee E, Bang JH. High Prevalence of Human Immunodeficiency Virus Infection among Inmates in Korean Correctional Facilities. *J Korean Med Sci.* 2021;36(14):e92.

18. Chuang DM, Newman PA, Fang L, Lai MC. Syndemic Conditions, Sexual Risk Behavior, and HIV Infection Among Men Who Have Sex with Men in Taiwan. *AIDS Behav.* 2021;25(11):3503-3518.

19. Coyer L, Boyd A, Davidovich U, van Bilsen WPH, Prins M, Matser A. Increase in recreational drug use between 2008 and 2018: results from a prospective cohort study among HIV-negative men who have sex with men. *Addiction.* 2022;117(3):656-665.

20. Coyer L, Prins M, Davidovich U, et al. Trends in Sexual Behavior and Sexually Transmitted Infections After Initiating Human Immunodeficiency Virus Pre-Exposure Prophylaxis in Men Who Have Sex with Men from Amsterdam, the Netherlands: A Longitudinal Exposure-Matched Study. *AIDS Patient Care STDS.* 2022;36(6):208-218.

21. Croxford S, Emanuel E, Shah A, et al. Epidemiology of HIV infection and associated behaviours among people who inject drugs in England, Wales, and Northern Ireland: Nearly 40 years on. *HIV Med.* 2022;23(9):978-989.

22. Daans CG, Hoornenborg E, de Haseth KB, et al. HIV Prevalence and High-Risk Subgroup Identification in Transgender Women Who Undergo Primary Vaginoplasty in the Netherlands. *Transgend Health.* 2023;8(3):226-230.

23. de la Court F, Boyd A, Davidovich U, et al. Eligibility criteria vs. need for pre-exposure prophylaxis: a reappraisal among men who have sex with men in Amsterdam, the Netherlands. *Epidemiol Infect.* 2022;150:e190.

24. de la Court F, Boyd A, Coyer L, et al. The impact of COVID-19-related restrictions in 2020 on sexual healthcare use, pre-exposure prophylaxis use, and sexually transmitted infection incidence among men who have sex with men in Amsterdam, the Netherlands. *HIV Med.* 2023;24(2):212-223.

25. de Sousa AFL, de Oliveira LB, Queiroz A, et al. Casual Sex among Men Who Have Sex with Men (MSM) during the Period of Sheltering in Place to Prevent the Spread of COVID-19. *Int J Environ Res Public Health.* 2021;18(6).

26. Dharan NJ, Jin F, Vaccher S, et al. Characteristics of Human Immunodeficiency Virus (HIV) Seroconversions in a Large Prospective Implementation Cohort Study of Oral HIV Preexposure Prophylaxis in Men Who Have Sex with Men (EPIC-NSW). *Clin Infect Dis.* 2023;76(3):e622-e628.

27. Drückler S, Daans C, Hoornenborg E, et al. HIV and STI positivity rates among transgender people attending two large STI clinics in the Netherlands. *Sex Transm Infect.* 2022;98(3):188-196.

28. European Centre for Disease Prevention and Control (ECDC). EMIS-2017: The European men-who-have-sex-with-men internet survey: Key findings from 50 countries. 2019; https://www.ecdc.europa.eu/sites/default/files/documents/European-MSM-internet-survey-2017-findings.pdf. Accessed 11 September 2024.

29. European Centre for Disease Prevention and Control (ECDC). HIV and sex workers: Monitoring the implementation of the Dublin Declaration on partnership to fight HIV/AIDS in Europe and Central Asia: 2022 progress report. 2024; https://www.ecdc.europa.eu/sites/default/files/documents/HIV-and-sex-workers-2022.pdf. Accessed 11 September 2024.

30. Casey C, O'Donnell K, Brady M, Igoe D. EMIS-2017 Ireland findings from the European men who have sex with men internet survey (Ireland). Dublin, Ireland. 2019; https://www.hpsc.ie/a-z/specificpopulations/menwhohavesexwithmenmsm/msminternetsurveys/emis-2017/emis-2017reportandinfographics/EMIS%20FINAL.pdf. Accessed 11 September 2024.

31. Schmidt AJ, Hickson F, Reid D, Weatherburn P. European online survey for men who have sex with men (EMIS-2017): Results in Spain. 2020; https://www.sanidad.gob.es/ciudadanos/enfLesiones/enfTransmisibles/sida/docs/EMIS_Report_07052020.pdf. Accessed 11 September 2024.

32. Estcourt C, Yeung A, Nandwani R, et al. Population-level effectiveness of a national HIV preexposure prophylaxis programme in MSM. *Aids.* 2021;35(4):665-673.

33. Evers YJ, Goense CJD, Hoebe C, Dukers-Muijrers N. Newly diagnosed HIV and use of HIV-PrEP among non-western born MSM attending STI clinics in the Netherlands: a large retrospective cohort study. *Front Public Health.* 2023;11:1196958.

34. Fernàndez-López L, Reyes-Urueña J, Conway A, et al. The contribution of HIV point-of-care tests in early HIV diagnosis: community-based HIV testing monitoring in Catalonia, 1995 to 2018. *Euro Surveill.* 2020;25(43).

35. Fernandez-Rollan L, Stuardo AV, Strömdahl S. Correlates of condomless anal intercourse among men who have sex with men in Santiago de Chile. *Int J STD AIDS.* 2019;30(3):231-240.

36. Ferrer L, González V, Martró E, et al. High HIV/STI prevalence among cisgender men and transgender women sex workers attending community-based centres in Barcelona, Spain: The Sweetie Project. *Int J STD AIDS.* 2022;33(12):1045-1053.

37. Fina L, Phillips AL, Jones AT, et al. Early experience of implementing a national HIV pre-exposure prophylaxis service in Wales, United Kingdom 2017. *Sex Health.* 2019;16(1):56-62.

38. Fortier E, Sylvestre MP, Artenie AA, et al. Associations between housing stability and injecting frequency fluctuations: findings from a cohort of people who inject drugs in Montréal, Canada. *Drug Alcohol Depend.* 2020;206:107744.

39. Gahrton C, Westman G, Lindahl K, et al. Prevalence of Viremic hepatitis C, hepatitis B, and HIV infection, and vaccination status among prisoners in Stockholm County. *BMC Infect Dis.* 2019;19(1):955.

40. García-Pérez JN, Cañas-Ruano E, Navarro J, et al. Sexual behavior and drug use impact in gay, bisexual, and other men who have sex with men. *Med Clin (Barc).* 2022;159(12):563-568.

41. Garros A, Bourrely M, Sagaon-Teyssier L, et al. Risk of Fecal Incontinence Following Receptive Anal Intercourse: Survey of 21,762 Men Who Have Sex With Men. *J Sex Med.* 2021;18(11):1880-1890.

42. Gasbarrini N, Dubravić D, Combs L, et al. Increasing integrated testing in community settings through interventions for change, including the Spring European Testing Week. *BMC Infect Dis.* 2021;21(Suppl 2):874.

43. Goddard SL, Templeton DJ, Petoumenos K, et al. Prevalence and Association of Perianal and Intra-Anal Warts with Composite High-Grade Squamous Intraepithelial Lesions Among Gay and Bisexual Men: Baseline Data from the Study of the Prevention of Anal Cancer. *AIDS Patient Care STDS.* 2020;34(10):436-443.

44. Government of the Netherlands. Numerical final evaluation PrEP pilot. 2023; <https://www.rijksoverheid.nl/documenten/rapporten/2023/07/27/cijfermatige-eindevaluatie-prep-pilot>. Accessed 11 September 2024.

45. Gratrix J, Smyczek P, Bertholet L, et al. A cross-sectional evaluation of opt-in testing for sexually transmitted and blood-borne infections in three Canadian provincial correctional facilities: a missed opportunity for public health? *Int J Prison Health.* 2019;15(3):273-281.

46. Greenwald ZR, Maheu-Giroux M, Szabo J, et al. Cohort profile: l'Actuel Pre-Exposure Prophylaxis (PrEP) Cohort study in Montreal, Canada. *BMJ Open.* 2019;9(6):e028768.

47. Grimshaw C, Boyd L, Smith M, Estcourt CS, Metcalfe R. Evaluation of an inner city HIV pre-exposure prophylaxis service tailored to the needs of people who inject drugs. *HIV Med.* 2021;22(10):965-970.

48. Grulich AE, Guy R, Amin J, et al. Population-level effectiveness of rapid, targeted, high-coverage roll-out of HIV pre-exposure prophylaxis in men who have sex with men: the EPIC-NSW prospective cohort study. *Lancet HIV.* 2018;5(11):e629-e637.

49. Grulich A, Jin F, Vaccher S, et al. Continuing low HIV incidence in the expanded pre-exposure prophylaxis (PrEP) implementation in communities - New South Wales study (EPIC-NSW) [Abstract TUAC0201]. Presented at: 10th IAS Conference on HIV Science. 2019.

50. Grulich AE, Jin F, Bavinton BR, et al. Long-term protection from HIV infection with oral HIV pre-exposure prophylaxis in gay and bisexual men: findings from the expanded and extended EPIC-NSW prospective implementation study. *Lancet HIV.* 2021;8(8):e486-e494.

51. Guerras JM, Hoyos J, de la Fuente L, et al. Awareness and Use of HIV Self-Testing Among Men Who Have Sex With Men Remains Low in Spain 2 Years After Its Authorization. *Front Public Health.* 2022;10:888059.

52. Hadjikou A, Pantavou K, Pavlitina E, et al. Sociometric Risk Network Structure, HIV Prevalence, and Drug Injection-Related Norms among People Who Inject Drugs (PWID) in Athens, Greece. *Subst Use Misuse.* 2021;56(8):1190-1201.

53. Hanum N, Cambiano V, Sewell J, et al. Substantial decline in HIV incidence between 2015 – 2018 among a prospective cohort of men who have sex with men in England [Meeting abstract P106]. *HIV Medicine.* 2019:1.

54. Hanum N, Cambiano V, Sewell J, et al. Trends in HIV incidence between 2013-2019 and association of baseline factors with subsequent incident HIV among gay, bisexual, and other men who have sex with men attending sexual health clinics in England: A prospective cohort study. *PLoS Med.* 2021;18(6):e1003677.

55. Harney BL, Agius PA, El-Hayek C, et al. Risk of Subsequent HIV Infection Following Sexually Transmissible Infections Among Men Who Have Sex With Men. *Open Forum Infect Dis.* 2019;6(10):ofz376.

56. Hart TA, Moore DM, Noor SW, et al. Prevalence of HIV and sexually transmitted and blood-borne infections, and related preventive and risk behaviours, among gay, bisexual and other men who have sex with men in Montreal, Toronto and Vancouver: results from the Engage Study. *Can J Public Health.* 2021;112(6):1020-1029.

57. Health Protection Surveillance Centre (HPSC). World AIDS Day: Update on epidemiology of HIV in Ireland, November 2022. 2022; <https://www.lenus.ie/bitstream/handle/10147/634954/World%20AIDS%20Day%20-%20Update%20on%20Epidemiology%20of%20HIV%20in%20Ireland.pdf?sequence=1>. Accessed 11 September 2024.

58. Holt M, Lea T, Bear B, et al. Trends in Attitudes to and the Use of HIV Pre-exposure Prophylaxis by Australian Gay and Bisexual Men, 2011-2017: Implications for Further Implementation from a Diffusion of Innovations Perspective. *AIDS Behav.* 2019;23(7):1939-1950.

59. Holt M, MacGibbon J, Bavinton B, et al. COVID-19 Vaccination Uptake and Hesitancy in a National Sample of Australian Gay and Bisexual Men. *AIDS Behav.* 2022;26(8):2531-2538.

60. Hoornenborg E, Coyer L, Achterbergh RCA, et al. Sexual behaviour and incidence of HIV and sexually transmitted infections among men who have sex with men using daily and event-driven pre-exposure prophylaxis in AMPrEP: 2 year results from a demonstration study. *Lancet HIV.* 2019;6(7):e447-e455.

61. Howarth AR, Saunders J, Reid D, et al. 'Stay at home …': exploring the impact of the COVID-19 public health response on sexual behaviour and health service use among men who have sex with men: findings from a large online survey in the UK. *Sex Transm Infect.* 2022;98(5):346-352.

62. Hoyos-Mallecot Y, Garcia JN, Sulleiro E, et al. Drassanes Exprés: a public and confidential testing service for asymptomatic STIs with same-day result notification. *Sex Transm Infect.* 2022;98(3):166-172.

63. Ikushima T, Yamaguchi M, Miwa T, Otsuki T. Survey on sex life and PrEP. 2022; <https://prep.ptokyo.org/wp/wp-content/uploads/2022/04/prepinjapan_report_R3.pdf>. Accessed 11 September 2024.

64. Iniesta C, Coll P, Barberá MJ, et al. Implementation of pre-exposure prophylaxis programme in Spain. Feasibility of four different delivery models. *PLoS One.* 2021;16(2):e0246129.

65. Izquierdo L, Mellon G, Buchaillet C, et al. Prevalence of hepatitis E virus and reassessment of HIV and other hepatitis virus seroprevalences among French prison inmates. *PLoS One.* 2019;14(6):e0218482.

66. Jourdain H, de Gage SB, Desplas D, Dray-Spira R. Real-world effectiveness of pre-exposure prophylaxis in men at high risk of HIV infection in France: a nested case-control study. *Lancet Public Health.* 2022;7(6):e529-e536.

67. Kåberg M, Karlsson N, Discacciati A, et al. Significant decrease in injection risk behaviours among participants in a needle exchange programme. *Infect Dis (Lond).* 2020;52(5):336-346.

68. Karlsson N, Kåberg M, Berglund T, Hammarberg A, Widman L, Ekström AM. A prospective cohort study of risk behaviours, retention and loss to follow-up over 5 years among women and men in a needle exchange program in Stockholm, Sweden. *Int J Drug Policy.* 2021;90:103059.

69. Keane A, Regan SO, Quinn L, et al. Evaluation of the impact of human immunodeficiency virus pre-exposure prophylaxis on new human immunodeficiency virus diagnoses during the COVID-19 pandemic. *Int J STD AIDS.* 2022;33(1):99-102.

70. Keen P, Patel P, McManus H, et al. Increased targeted HIV testing and reduced undiagnosed HIV infections among gay and bisexual men in New South Wales, Australia 2010 to 2018 [Oral Abstract OAC0205]. *Journal of the International AIDS Society.* 2020;23(S4):e25547.

71. King J, McManus H, Kwon A, Gray R, McGregor S. HIV, viral hepatitis and sexually transmissible infections in Australia. Annual surveillance report 2022. 2022; <https://www.kirby.unsw.edu.au/sites/default/files/documents/Annual-Surveillance-Report-2022_HIV.pdf>. Accessed 11 September 2024.

72. Kirby Institute. National HIV strategy indicators. 2022; <https://public.tableau.com/views/NationalHIVStrategy2022/1b1cGayandbisexualmentesting?%3AshowVizHome=no>. Accessed 11 September 2024.

73. Kirby Institute. Australian NSP survey national data report 2018-2022: Prevalence of HIV, HCV and injecting and sexual behaviour among NSP attendees. 2023; <https://www.kirby.unsw.edu.au/research/reports/australian-nsp-survey-national-data-report-2018-2022>. Accessed 11 September 2024.

74. Ku SW, Chen TW, Li CW, et al. Preferences for potential long-acting pre-exposure prophylaxis (PrEP) dosing regimens among gay, bisexual and other men who have sex with men (GBMSM) in Taiwan: 2021 HEART Survey [Abstract # PESUC30]. The 24th International AIDS Conference; July 29- 2 August, 2022; Montreal, Canada.

75. Kwan TH, Chan DPC, Wong SY, Lee SS. Implementation Cascade of a Social Network-Based HIV Self-testing Approach for Men Who Have Sex With Men: Cross-sectional Study. *J Med Internet Res.* 2023;25:e46514.

76. Lambert G, Cox J, Fourmigue A, et al. HIV incidence and related risks among gay, bisexual, and other men who have sex with men in Montreal, Toronto, and Vancouver: Informing blood donor selection criteria in Canada. *Transfusion.* 2022;62(12):2555-2567.

77. Lapadula G, Soria A, Modesti M, et al. Behavioural survey and street-based HIV and HCV rapid testing programme among transgender sex workers. *Sex Transm Infect.* 2023;99(1):41-46.

78. Lopez-Corbeto E, Sanclemente C, González V, Mansilla R, Casabona J, Folch C. HIV, Chlamydia and gonorrhoea vulnerability depending to sex work site. *Enferm Infecc Microbiol Clin (Engl Ed).* 2022;40(4):166-171.

79. MacGibbon J, Bavinton BR, Drysdale K, et al. Explicit Relationship Agreements and HIV Pre-exposure Prophylaxis Use by Gay and Bisexual Men in Relationships. *Arch Sex Behav.* 2023;52(2):761-771.

80. Marco A, Guerrero RA, Vergara M, et al. Reinfection in a large cohort of prison inmates with sustained virological response after treatment of chronic hepatitis C in Catalonia (Spain), 2002-2016. *Int J Drug Policy.* 2019;72:189-194.

81. McAuley A, Palmateer NE, Goldberg DJ, et al. Re-emergence of HIV related to injecting drug use despite a comprehensive harm reduction environment: a cross-sectional analysis. *Lancet HIV.* 2019;6(5):e315-e324.

82. Meulbroek M, Dalmau-Bueno A, Saz J, et al. Falling HIV incidence in a community clinic cohort of men who have sex with men and transgender women in Barcelona, Spain. *Int J STD AIDS.* 2020;31(9):841-848.

83. Mitchell HD, Desai S, Mohammed H, et al. Preparing for PrEP: estimating the size of the population eligible for HIV pre-exposure prophylaxis among men who have sex with men in England. *Sex Transm Infect.* 2019;95(7):484-487.

84. Molina JM, Charreau I, Spire B, et al. Efficacy, safety, and effect on sexual behaviour of on-demand pre-exposure prophylaxis for HIV in men who have sex with men: an observational cohort study. *Lancet HIV.* 2017;4(9):e402-e410.

85. Molina J-M, Ghosn J, Algarte-Génin M, et al. Incidence of HIV-Infection with Daily or On demand PrEP with TDF/FTC in the Paris Area: An update of the Prevenir Study. Presented at: 10th IAS Conference on HIV Science. 2019.

86. Molina JM, Ghosn J, Assoumou L, et al. Daily and on-demand HIV pre-exposure prophylaxis with emtricitabine and tenofovir disoproxil (ANRS PREVENIR): a prospective observational cohort study. *Lancet HIV.* 2022;9(8):e554-e562.

87. Nelson LE, Tharao W, Husbands W, et al. The epidemiology of HIV and other sexually transmitted infections in African, Caribbean and Black men in Toronto, Canada. *BMC Infect Dis.* 2019;19(1):294.

88. Ogaz D, Logan L, Curtis TJ, et al. PrEP use and unmet PrEP-need among men who have sex with men in London prior to the implementation of a national PrEP programme, a cross-sectional study from June to August 2019. *BMC Public Health.* 2022;22(1):1105.

89. Ontario HIV Epidemiology and Surveillance Initiative. HIV epidemiology update for gay, bisexual, and other men who have sex with men (GBMSM) in Ontario, 2019. 2021; <https://www.ohesi.ca/wp-content/uploads/2022/05/GBMSM-Factsheet-ENGLISH-2022MAY04.pdf>. Accessed 11 September 2024.

90. Patel PG, Keen P, McManus H, et al. Increased targeted HIV testing and reduced undiagnosed HIV infections among gay and bisexual men. *HIV Med.* 2021;22(7):605-616.

91. Pavlopoulou ID, Dikalioti SK, Gountas I, et al. High-risk behaviors and their association with awareness of HIV status among participants of a large-scale prevention intervention in Athens, Greece. *BMC Public Health.* 2020;20(1):105.

92. Pečavar B, Kokošar Ulčar B, Kordiš M, et al. Pre-exposure prophylaxis for HIV with oral tenofovir disoproxil fumarate/emtricitabine in men who have sex with men: Slovenian national demonstration project. *Int J STD AIDS.* 2021;32(11):1060-1065.

93. Public Health Agency of Canada. Estimates of HIV incidence, prevalence and Canada’s progress on meeting the 90-90-90 HIV targets, 2020. 2022; <https://www.canada.ca/content/dam/phac-aspc/documents/services/publications/diseases-conditions/estimates-hiv-incidence-prevalence-canada-meeting-90-90-90-targets-2020/estimates-hiv-incidence-prevalence-canada-meeting-90-90-90-targets-2020.pdf>. Accessed 11 September 2024.

94. Public Health Agency of Canada. HIV in Canada - People living with HIV and new HIV infections, 2020. 2020; <https://www.canada.ca/en/public-health/services/publications/diseases-conditions/hiv-canada-people-living-with-hiv-new-infections-2020.html>. Accessed 11 September 2024.

95. Public Health Agency of Sweden (Folkhalsomyndigheten). Swedish results from the European Internet survey EMIS-2017 – A study of men who have sex with men (MSM). 2020; <https://www.folkhalsomyndigheten.se/publikationer-och-material/publikationsarkiv/e/emis-2017/?pub=82518>. Accessed 11 September 2024.

96. Prinsenberg T, Schinkel J, Zantkuijl P, Davidovich U, Prins M, van der Valk M. Internet-guided HCV-RNA testing: A promising tool to achieve hepatitis C micro-elimination among men who have sex with men. *J Viral Hepat.* 2022;29(8):677-684.

97. Rahib D, Bercot B, Delagreverie H, et al. Online self-sampling kits for human immunodeficiency virus and other sexually transmitted infections: Feasibility, positivity rates, and factors associated with infections in France. *Int J STD AIDS.* 2022;33(4):355-362.

98. Richardson L, Mammel M, Milloy MJ, Hayashi K. Employment Cessation, Long Term Labour Market Engagement and HIV Infection Risk Among People Who Inject Drugs in an Urban Canadian Setting. *AIDS Behav.* 2019;23(12):3267-3276.

99. Roussos S, Paraskevis D, Psichogiou M, et al. Ongoing HIV transmission following a large outbreak among people who inject drugs in Athens, Greece (2014-20). *Addiction.* 2022;117(6):1670-1682.

100. Ryan K, Asselin J, Fairley C, et al. Results from a Large Australian PrEP Demonstration Study: Discontinuation and Subsequent HIV and Other Sexually Transmitted Infection Risk [Abstract MOAD0303]. Presented at: IAS. . 2019.

101. Ryan P, Valencia J, Cuevas G, et al. HIV screening and retention in care in people who use drugs in Madrid, Spain: a prospective study. *Infect Dis Poverty.* 2021;10(1):111.

102. Samji H, Hu J, Otterstatter M, et al. Gay, bisexual, and other men who have sex with men accessing STI clinics: Optimizing HIV PrEP implementation. *PLoS One.* 2022;17(1):e0261705.

103. Sang JM, Cui Z, Sereda P, et al. Longitudinal Event-Level Sexual Risk and Substance Use among Gay, Bisexual, and Other Men Who Have Sex with Men. *Int J Environ Res Public Health.* 2021;18(6).

104. Santos GM, Ackerman B, Rao A, et al. Economic, Mental Health, HIV Prevention and HIV Treatment Impacts of COVID-19 and the COVID-19 Response on a Global Sample of Cisgender Gay Men and Other Men Who Have Sex with Men. *AIDS Behav.* 2021;25(2):311-321.

105. Saxton PJW, McAllister SM, Noller GE, Newcombe DAL, Leafe KA. Injecting drug use among gay and bisexual men in New Zealand: Findings from national human immunodeficiency virus epidemiological and behavioural surveillance. *Drug Alcohol Rev.* 2020;39(4):365-374.

106. Saxton PJW, McAllister SM, Thirkell CE, et al. Population rates of HIV, gonorrhoea and syphilis diagnoses by sexual orientation in New Zealand. *Sex Transm Infect.* 2022;98(5):376-379.

107. Scheim A, Knight R, Shulha H, et al. Characterizing Men Who Have Sex with Men and Use Injection Drugs in Vancouver, Canada. *AIDS Behav.* 2019;23(12):3324-3330.

108. Schmidt AJ, Rasi M, Esson C, et al. The Swiss STAR trial - an evaluation of target groups for sexually transmitted infection screening in the sub-sample of men. *Swiss Med Wkly.* 2020;150:w20392.

109. Schmidt D, Kollan C, Bartmeyer B, et al. Low incidence of HIV infection and decreasing incidence of sexually transmitted infections among PrEP users in 2020 in Germany. *Infection.* 2023;51(3):665-678.

110. Sekera JC, Frýbert J. Analysis of drug-related infectious diseases in people who inject drugs - Pilsen Region, 2003-2018. *Cent Eur J Public Health.* 2022;30(1):13-19.

111. Seviy S, Koletiy G. Research on the prevalence of HIV, hepatitis C and risky behaviors in people who inject drugs in Croatia – Bio-behavioral research using using the method of sampling controlled respondents in Zagreb, Split and Rijeka. 2022; <https://www.hzjz.hr/wp-content/uploads/2023/01/ISTRAZ%CC%8CIVANJE-PREVALENCIJE-web.pdf>. Accessed 11 September 2024.

112. Silvestri C, Stasi C, Lazzeretti M, Voller F. Substance Abuse Disorder and Viral Infections (Hepatitis, HIV): A Multicenter Study in Tuscan Prisons. *J Correct Health Care.* 2021;27(3):161-166.

113. Hernando V, Cobos M, Nuno N, SIPrEP Gdtd. Information system for HIV pre-exposure prophylaxis programs in Spain (SIPrEP): Results report November 2019-May 2023. 2023; <https://www.sanidad.gob.es/ciudadanos/enfLesiones/enfTransmisibles/sida/PrEP/Informe_SIPrEP_nov_19_mayo_23.pdf>. Accessed 11 September 2024.

114. División de Control de VIH ITS Hepatitis virales y Tuberculosis DGdSP, Sanidad,. Implementation of prophylaxis preexposure (PrEP) in the National Health System. 2021; <https://siprep.isciii.es/documents/20125/36963/Informe+situaci%C3%B3n+implementaci%C3%B3n+de+la+profilaxis+Preexposici%C3%B3n+%28PrEP%29+en+el+Sistema+Nacional+de+Salud.pdf/bca1d176-a022-3c9c-22ce-0bb37c1ccb84?t=1641972552890&download=true>. Accessed 11 September 2024.

115. Skaletz-Rorowski A, Potthoff A, Nambiar S, et al. Online HIV/STI Risk Test (ORT): A prospective cross-sectional study among sexually active individuals in Germany. *J Dtsch Dermatol Ges.* 2022;20(3):306-314.

116. Slurink I, van Aar F, Parkkali S, et al. Recently acquired HIV infections and associated factors among men who have sex with men diagnosed at Dutch sexual health centres. *Int J STD AIDS.* 2021;32(10):946-956.

117. Steedman N, Wallace L, Estcourt C, et al. Uptake and STI epidemiology from the first year of the NHS HIV PrEP programme in Scotland. 2019; <https://www.bhiva.org/file/5ca756f28e49f/NicolaSteedman.pdf>. Accessed 11 September 2024.

118. Stevens O, Moncrieff M, Gafos M. Chemsex-related drug use and its association with health outcomes in men who have sex with men: a cross-sectional analysis of Antidote clinic service data. *Sex Transm Infect.* 2020;96(2):124-130.

119. Stojanovski K, King EJ, Amico KR, et al. Stigmatizing Policies Interact with Mental Health and Sexual Behaviours to Structurally Induce HIV Diagnoses Among European Men Who Have Sex with Men. *AIDS Behav.* 2022;26(10):3400-3410.

120. Streeck H, Janssen K, Crowell TA, et al. Prospective, multicenter study to assess point prevalence, incidence and recurrence of sexually transmitted infections in men who have sex with men in Germany: BRAHMS study [Oral Abstract TUPDC0106]. Presented at: 10th International AIDS Society (IAS). 2019.

121. Stuardo Ávila V, Fuentes Alburquenque M, Muñoz R, et al. Prevalence and Risk Factors for HIV Infection in a Population of Homosexual, Bisexual, and Other Men Who Have Sex with Men in the Metropolitan Region of Chile: A Re-emerging Health Problem. *AIDS Behav.* 2020;24(3):827-838.

122. Public Health Agency of Canada. HIV and hepatitis C care and treatment services: Survey report among Indigenous participants who inject drugs in Canada, 2017-2019: Infographic. 2021; <https://www.canada.ca/en/public-health/services/publications/diseases-conditions/hiv-hepatitis-c-care-treatment-services-survey-report-indigenous-inject-drugs-2017-2019.html>. Accessed 11 September 2024.

123. Lydon-Hassen K, Jonah L, Mayotte L, et al. Summary findings from Tracks surveys implemented by First Nations in Saskatchewan and Alberta, Canada, 2018-2020. *Can Commun Dis Rep.* 2022;48(4):146-156.

124. Canada Communicable Disease Report (CCDR). Determinants of HIV and hepatitis C among people who inject drugs in Canada, 2017-2019. 2020; <https://www.canada.ca/en/public-health/services/reports-publications/canada-communicable-disease-report-ccdr/monthly-issue/2020-46/issue-5-may-7-2020/infographic-tracks-survey-canada.html>. Accessed 11 September 2024.

125. Tarasuk J, Sullivan M, Bush D, et al. Findings among Indigenous participants of the Tracks survey of people who inject drugs in Canada, Phase 4, 2017-2019. *Can Commun Dis Rep.* 2021;47(1):37-46.

126. Tarasuk J, Zhang J, Lemyre A, Cholette F, Bryson M, Paquette D. National findings from the Tracks survey of people who inject drugs in Canada, Phase 4, 2017-2019. *Can Commun Dis Rep.* 2020;46(5):138-148.

127. Tassi MF, Laurent E, Gras G, et al. PrEP monitoring and HIV incidence after PrEP initiation in France: 2016-18 nationwide cohort study. *J Antimicrob Chemother.* 2021;76(11):3002-3008.

128. Trayner KMA, McAuley A, Palmateer NE, et al. Increased risk of HIV and other drug-related harms associated with injecting in public places: national bio-behavioural survey of people who inject drugs. *Int J Drug Policy.* 2020;77:102663.

129. UK Health Security Agency. Official Statistics. HIV testing, PrEP, new HIV Diagnoses and care outcomes for people accessing HIV services: 2023 report. 2023; <https://www.gov.uk/government/statistics/hiv-annual-data-tables/hiv-testing-prep-new-hiv-diagnoses-and-care-outcomes-for-people-accessing-hiv-services-2023-report>. Accessed 11 September 2024.

130. UNAIDS. Country factsheets: Estonia 2022. 2022; <https://www.unaids.org/en/regionscountries/countries/estonia>. Accessed 11 September 2024.

131. UNAIDS. Country Factsheets. 2022; <https://aidsinfo.unaids.org/>. Accessed 11 September 2024.

132. UNAIDS. UNAIDS Key Population Atlas. 2023; <https://kpatlas.unaids.org/dashboard>. Accessed 11 September 2024.

133. van Bilsen WPH, Boyd A, van der Loeff MFS, et al. Diverging trends in incidence of HIV versus other sexually transmitted infections in HIV-negative MSM in Amsterdam. *Aids.* 2020;34(2):301-309.

134. Van Meer R, Antoniou T, McCormack D, et al. A comparison of the observed and expected prevalence of HIV in persons released from Ontario provincial prisons in 2010. *Can J Public Health.* 2019;110(5):675-682.

135. van Santen DK, Boyd A, Matser A, et al. The effect of needle and syringe program and opioid agonist therapy on the risk of HIV, hepatitis B and C virus infection for people who inject drugs in Amsterdam, the Netherlands: findings from an emulated target trial. *Addiction.* 2021;116(11):3115-3126.

136. Velter A, Sauvage C, Saboni L, et al. Estimation of HIV prevalence among men who have sex with men frequenting gay social venues in five French cities - PREVAGAY 2015. *Bulletin epidemiologique hebdomadaire.* 2017;18:347-354.

137. Sauvage C, Saboni L, Trouiller-Gerfaux P, Sommen C, Alexandre A, Lydie N. PREVAGAY Report 2015: HIV seroprevalence survey carried out among men who have sexual relations with men frequenting gay friendly places. France. 2017.

138. Velter A, Duchesne L, Lydié N. Evolution of prevention behaviors in men having sex with men in France- 2017 and 2019 gender report surveys. 2019; <https://www.santepubliquefrance.fr/maladies-et-traumatismes/infections-sexuellement-transmissibles/vih-sida/documents/communication-congres/evolution-des-comportements-de-prevention-chez-les-hommes-ayant-des-rapports-sexuels-avec-des-hommes-en-france-enquetes-rapport-au-sexe-2017-et-2019>. Accessed 11 September 2024.

139. Velter A, Ousseine YM, Duchesne L, Lydie N. Non-use of combination HIV prevention tools and its determinants among men who have sex with men living in France. *Infect Dis Now.* 2022;52(6):341-348.

140. Vernazza PL, Rasi M, Ritzler M, et al. The Swiss STAR trial - an evaluation of target groups for sexually transmitted infection screening in the sub-sample of women. *Swiss Med Wkly.* 2020;150:w20393.

141. Vu F, Cavassini M, D'Acremont V, et al. Epidemiology of sexually transmitted infections among female sex workers in Switzerland: a local, exploratory, cross-sectional study. *Swiss Med Wkly.* 2020;150:w20357.

142. Vuylsteke B, Reyniers T, De Baetselier I, et al. Daily and event-driven pre-exposure prophylaxis for men who have sex with men in Belgium: results of a prospective cohort measuring adherence, sexual behaviour and STI incidence. *J Int AIDS Soc.* 2019;22(10):e25407.

143. Wayal S, Reid D, Weatherburn P, et al. Association between knowledge, risk behaviours, and testing for sexually transmitted infections among men who have sex with men: findings from a large online survey in the United Kingdom. *HIV Med.* 2019;20(8):523-533.

144. Wiessing L, Kalamara E, Stone J, et al. Univariable associations between a history of incarceration and HIV and HCV prevalence among people who inject drugs across 17 countries in Europe 2006 to 2020 - is the precautionary principle applicable? *Euro Surveill.* 2021;26(49).

145. Wong NS, Kwan TH, Lee KCK, Lau JYC, Lee SS. Delineation of chemsex patterns of men who have sex with men in association with their sexual networks and linkage to HIV prevention. *Int J Drug Policy.* 2020;75:102591.

146. Annequin M, Villes V, Delabre RM, et al. Are PrEP services in France reaching all those exposed to HIV who want to take PrEP? MSM respondents who are eligible but not using PrEP (EMIS 2017). *AIDS Care.* 2020;32(sup2):47-56.

147. Basten M, den Daas C, Heijne JCM, et al. The Rhythm of Risk: Sexual Behaviour, PrEP Use and HIV Risk Perception Between 1999 and 2018 Among Men Who Have Sex with Men in Amsterdam, The Netherlands. *AIDS Behav.* 2021;25(6):1800-1809.

148. Bavinton BR, Prestage GP, Jin F, et al. Strategies used by gay male HIV serodiscordant couples to reduce the risk of HIV transmission from anal intercourse in three countries. *J Int AIDS Soc.* 2019;22(4):e25277.

149. Bavinton BR, Hammoud MA, Holt M, et al. Changes in Sexual Behaviour Following PrEP Initiation Among Australian Gay and Bisexual Men in Relationships: Results from a Prospective Observational Study. *AIDS Behav.* 2021;25(11):3704-3711.

150. Bayley J, Nadarzynski T, Portman M, Owen G, Pasvol T, Nutland W. The trends in PrEP awareness, acceptability and uptake amongst MSM in London between 2016 and 2018 [Poster Abstract P133]. *HIV Medicine.* 2018;19(Suppl 2):s21–s152.

151. Vanden Berghe W, Deblonde J, Detandt S, Pezeril C, Sergeant M, Barris S. The European men who have sex with men internet survey (Issued) 2017: Results for Belgium. 2021; <https://www.sciensano.be/sites/default/files/emis_nl_2017_belgium.pdf>. Accessed 11 September 2024.

152. Bourne A, Alba B, Garner A, Spiteri G, Pharris A, Noori T. Use of, and likelihood of using, HIV pre-exposure prophylaxis among men who have sex with men in Europe and Central Asia: findings from a 2017 large geosocial networking application survey. *Sex Transm Infect.* 2019;95(3):187-192.

153. Buffel V, Reyniers T, Masquillier C, et al. Awareness of, Willingness to Take PrEP and Its Actual Use Among Belgian MSM at High Risk of HIV Infection: Secondary Analysis of the Belgian European MSM Internet Survey. *AIDS Behav.* 2022;26(6):1793-1807.

154. Bull L, Dimitrijevic P, Beverley S, et al. Perceived need of, and interest in, HIV pre-exposure prophylaxis amongst men who have sex with men attending three sexual health clinics in London, UK. *Int J STD AIDS.* 2018;29(5):435-442.

155. Callander D, Park SH, Al-Ajlouni YA, et al. Condomless Group Sex Is Associated With HIV Pre-Exposure Prophylaxis Knowledge and Interest Uptake: A Cross-Sectional Study of Gay and Bisexual Men in Paris, France. *AIDS Educ Prev.* 2019;31(2):127-135.

156. Callander D, Wiggins J, Rosenberg S, et al. The 2018 Australian Trans and Gender Diverse Sexual Health Survey: Report of Findings. 2019; <https://www.kirby.unsw.edu.au/sites/default/files/documents/ATGD-Sexual-Health-Survey-Report_2018.pdf>. Accessed 11 September 2024.

157. Chan C, Bavinton B, Hammoud M, et al. The INTO? study: Report of results. 2020; <https://www.kirby.unsw.edu.au/sites/default/files/documents/The_INTO_Study_Report_of_results_2020.pdf>. Accessed 11 September 202.

158. Chan C, Fraser D, Schmidt HMA, et al. PrEP product awareness, preferences, and past experiences among transgender women and men who have sex with men in Asia and Australia: The PrEP APPEAL study report. 2023; <https://www.kirby.unsw.edu.au/sites/default/files/documents/PrEP-Preferences-in-Asia-and-Australia-Report-Final_2023_1.pdf>. Accessed 11 September 2024.

159. Colyer S, Lachowsky NJ, Schmidt AJ, et al. Measures of HIV Pre-exposure Prophylaxis Uptake Among Gay, Bisexual, and Other Men Who Have Sex with Men in Canada and Demographic Disparities Among Those at Elevated Likelihood for HIV Acquisition. *AIDS Behav.* 2021;25(11):3638-3650.

160. Coyer L, van Bilsen W, Bil J, et al. Pre-exposure prophylaxis among men who have sex with men in the Amsterdam Cohort Studies: Use, eligibility, and intention to use. *PLoS One.* 2018;13(10):e0205663.

161. Di Ciaccio M, Villes V, Michels D, et al. Impact of the early 2020 COVID-19 crisis and lockdown on PrEP use among men who have sex with men (MSM) in France. *Sex Transm Infect.* 2022;98(7):510-517.

162. Duchesne L, Lydié N, Velter A. Increase in the overall level of protected anal sex in men who have sex with men in France: results from the repeated cross-sectional survey Rapport au Sexe, France, 2017-2019. *AIDS Care.* 2020;32(sup2):162-169.

163. European Centre for Disease Prevention and Control (ECDC). Evidence brief: Progress towards reaching the Sustainable Development Goals related to HIV in the EU/EEA. 2024; <https://www.ecdc.europa.eu/en/publications-data/evidence-brief-progress-towards-reaching-sustainable-development-goals-related>. Accessed 11 September 2024.

164. Fraser D, Chan C, Vaccher S, et al. Report on the PrEP in NSW transition study, 2018-2020. 2020; <https://www.kirby.unsw.edu.au/sites/default/files/documents/PrEP-in-NSW-Transition-Study-Report_Kirby-Institute_2020.pdf>. Accessed 11 September 2024.

165. Gaskins M, Sammons MK, Kutscha F, Nast A, Werner RN. Factors that motivate men who have sex with men in Berlin, Germany, to use or consider using HIV pre-exposure prophylaxis-A multi-methods analysis of data from a multicentre survey. *PLoS One.* 2021;16(11):e0260168.

166. Hambrick HR, Park SH, Schneider JA, et al. Poppers and PrEP: Use of Pre-exposure Prophylaxis Among Men Who Have Sex with Men Who Use Inhaled Nitrites. *AIDS Behav.* 2018;22(11):3658-3672.

167. Hammoud MA, Vaccher S, Jin F, et al. HIV Pre-exposure Prophylaxis (PrEP) Uptake Among Gay and Bisexual Men in Australia and Factors Associated With the Nonuse of PrEP Among Eligible Men: Results From a Prospective Cohort Study. *J Acquir Immune Defic Syndr.* 2019;81(3):e73-e84.

168. Hampel B, Kusejko K, Braun DL, Harrison-Quintana J, Kouyos R, Fehr J. Assessing the need for a pre-exposure prophylaxis programme using the social media app Grindr®. *HIV Med.* 2017;18(10):772-776.

169. Hanum N, Cambiano V, Sewell J, et al. Use of HIV pre-exposure prophylaxis among gay, bisexual, and other men who have sex with men (GBMSM) in England: data from the AURAH2 study [Abstract 020]. Paper presented at: 26th Annual Conference of the British HIV Association (BHIVA); November 22-24, 2020; Virtual.

170. Hanum N, Cambiano V, Sewell J, et al. Use of HIV pre-exposure prophylaxis among men who have sex with men in England: data from the AURAH2 prospective study. *Lancet Public Health.* 2020;5(9):e501-e511.

171. Van Sighem A, Wit F, Boyd A, Smit C, Jongen V, Koole J. HIV Monitoring Report 2023. Human Immunodeficiency Virus (HIV) Infection in the Netherlands. 2023; <https://www.hiv-monitoring.nl/en/resources/monitoring-report-2023>. Accessed 11 September 2024.

172. HIV Lens. HIV Lens. 2024; <https://www.hiv-lens.org/>. Accessed 11 September 2024.

173. Holt M, Lea T, Schmidt HM, et al. Willingness to use and have sex with men taking HIV pre-exposure prophylaxis (PrEP): results of online surveys of Australian gay and bisexual men, 2011-2015. *Sex Transm Infect.* 2017;93(6):438-444.

174. Holt M, Draper BL, Pedrana AE, Wilkinson AL, Stoové M. Comfort Relying on HIV Pre-exposure Prophylaxis and Treatment as Prevention for Condomless Sex: Results of an Online Survey of Australian Gay and Bisexual Men. *AIDS Behav.* 2018;22(11):3617-3626.

175. Holt M, Lee E, Lea T, et al. HIV Preexposure Prophylaxis Cascades to Assess Implementation in Australia: Results From Repeated, National Behavioral Surveillance of Gay and Bisexual Men, 2014-2018. *J Acquir Immune Defic Syndr.* 2020;83(3):e16-e22.

176. Hovaguimian F, Martin E, Reinacher M, et al. Participation, retention and uptake in a multicentre pre-exposure prophylaxis cohort using online, smartphone-compatible data collection. *HIV Med.* 2022;23(2):146-158.

177. Krist LC, Zimmermann HML, van Dijk M, Stutterheim SE, Jonas KJ. PrEP Use in Times of COVID-19 in the Netherlands: Men Who Have Sex With Men (MSM) on PrEP Test Less for HIV and Renal Functioning During a COVID-19 Related Lockdown. *AIDS Behav.* 2022;26(11):3656-3666.

178. Lachowsky NJ, Lawson Tattersall T, Sereda P, Wang C, Edwards J, Hull M. Community awareness of, use of and attitudes towards HIV pre-exposure prophylaxis (PrEP) among men who have sex with men in Vancouver, Canada: preparing health promotion for a publicly funded PrEP program. *Sex Health.* 2019;16(2):180-186.

179. Leobon A, Samson-Daoust E. Predictors Associated With Pre-Exposure Prophylaxis (PrEP) Efficacy Perception And Use Among Men Who Have Sex With Men And Transgender People Eligible For Prep In 2018, According To The Net Gay Barometer. *Bulletin epidemiologique hebdomadaire.* 2022;2:26-35.

180. Logan L, Fakoya I, Howarth A, et al. Combination prevention and HIV: a cross-sectional community survey of gay and bisexual men in London, October to December 2016. *Euro Surveill.* 2019;24(25).

181. MacGibbon J, Lea T, Ellard J, et al. Access to Subsidized Health Care Affects HIV Pre-Exposure Prophylaxis (PrEP) Uptake Among Gay and Bisexual Men in Australia: Results of National Surveys 2013-2019. *J Acquir Immune Defic Syndr.* 2021;86(4):430-435.

182. Meireles P, Fernandes F, Rocha M, Plankey M, Barros H. Provision of Preexposure Prophylaxis at the Portuguese National Health Service and Uptake in the Lisbon Cohort of Men Who Have Sex with Men. *AIDS Behav.* 2021;25(6):1975-1983.

183. Mosley T, Khaketla M, Armstrong HL, et al. Trends in Awareness and Use of HIV PrEP Among Gay, Bisexual, and Other Men who have Sex with Men in Vancouver, Canada 2012-2016. *AIDS Behav.* 2018;22(11):3550-3565.

184. Murray D, Mao L, Wong TH, et al. High levels of engagement with testing for HIV and sexually transmissible infection among gay Asian men in Sydney and Melbourne: an observational study. *Sex Health.* 2020;17(2):121-128.

185. Nguyen SN. Awareness and willingness of HIV Pre-Exposure Prophylaxis (PrEP) among young people in Sweden: An online survey. 2020; <https://www.diva-portal.org/smash/get/diva2:1684908/ATTACHMENT01.pdf>. Accessed 11 September 2024.

186. O'Byrne P, Vandyk A, Orser L, Haines M. Nurse-led PrEP-RN clinic: a prospective cohort study exploring task-Shifting HIV prevention to public health nurses. *BMJ Open.* 2021;11(1):e040817.

187. O'Halloran C, Owen G, Croxford S, et al. Current experiences of accessing and using HIV pre-exposure prophylaxis (PrEP) in the United Kingdom: a cross-sectional online survey, May to July 2019. *Euro Surveill.* 2019;24(48).

188. Pico-Espinosa OJ, Hull M, MacPherson P, et al. Reasons for not using pre-exposure prophylaxis for HIV and strategies that may facilitate uptake in Ontario and British Columbia among gay, bisexual and other men who have sex with men: a cross-sectional survey. *CMAJ Open.* 2023;11(3):E560-e568.

189. Sang JM, McAllister K, Wang L, et al. Examining provincial PrEP coverage and characterizing PrEP awareness and use among gay, bisexual and other men who have sex with men in Vancouver, Toronto and Montreal, 2017-2020. *J Int AIDS Soc.* 2022;25(10):e26017.

190. Sauvage C, Saboni L, Sommen C, et al. Pre-Exposure Prophylaxis Awareness And Use Among Men Who Have Sex With Men Attending Gay Venues In Five French Cities. PREVAGAY 2015. *Bulletin epidemiologique hebdomadaire.* 2018;29:602-610.

191. Saxby K, Chan C, Bavinton BR. Structural Stigma and Sexual Health Disparities Among Gay, Bisexual, and Other Men Who Have Sex With Men in Australia. *J Acquir Immune Defic Syndr.* 2022;89(3):241-250.

192. Spinner CD, Hanhoff N, Krznaric I, et al. 2016 PREP attitudes in Germany: high awareness and acceptance in MSM at risk of HIV. *Infection.* 2018;46(3):405-408.

193. Streeck H, Jansen K, Crowell TA, et al. HIV pre-exposure prophylaxis was associated with no impact on sexually transmitted infection prevalence in a high-prevalence population of predominantly men who have sex with men, Germany, 2018 to 2019. *Euro Surveill.* 2022;27(14).

194. Suen YT, Chan RCH, Wong EMY. Sex Conservatism, Internalized Homonegativity, PrEP Stigma, and Intention to Use PrEP: A Study of Chinese-Speaking Gay and Bisexual Male Youth in Hong Kong. *AIDS Educ Prev.* 2022;34(6):467-480.

195. Tan RKJ, Teo AKJ, Kaur N, Harrison-Quintana J, M ICC, Wong CS. Cost and anonymity as factors for the effective implementation of pre-exposure prophylaxis: an observational study among gay, bisexual and other men who have sex with men in Singapore. *Sex Health.* 2018;15(6):533-541.

196. Tooley L, Bachari AK. Advance national progress card. 2021; <https://www.advance-avancer.ca/publications/2020/12/8/advance-national-progress-cards-2020>. Accessed 11 September 2024.

197. UK Health Security Agency. Pre-exposure prophylaxis (PrEP) need and use at specialist sexual health services, by demographic group: England, 2021 to 2022. 2023; <https://view.officeapps.live.com/op/view.aspx?src=https%3A%2F%2Fassets.publishing.service.gov.uk%2Fmedia%2F6512df31f6746b0012a4ba5d%2F2022-PrEP-need-and-use-by-demographic-group.ods&wdOrigin=BROWSELINK>. Accessed 11 September 2024.

198. van Dijk M, Duken SB, Delabre RM, et al. PrEP Interest Among Men Who Have Sex with Men in the Netherlands: Covariates and Differences Across Samples. *Arch Sex Behav.* 2020;49(6):2155-2164.

199. van Dijk M, de Wit JBF, Guadamuz TE, Martinez JE, Jonas KJ. Slow Uptake of PrEP: Behavioral Predictors and the Influence of Price on PrEP Uptake Among MSM with a High Interest in PrEP. *AIDS Behav.* 2021;25(8):2382-2390.

200. Velter V, Ousseine Y, Dupire P, Roux P, Mercier A. Evolution of the level of protection against HIV among men who have sex with HIV-negative men – Results of the 2017-2019-2021 Sex Ratio survey. *Bulletin Epidemiologique Hebdomadaire.* 2022;24-25:430-438.

201. Walmsley B, Gallant D, Naccarato M, Hull M, Smith A, Tan DH. The PrEP You Want: A Web-Based Survey of Online Cross-Border Shopping for HIV Prophylaxis Medications. *J Med Internet Res.* 2019;21(7):e12076.

202. Wang Z, Lau JTF, Fang Y, Ip M, Gross DL. Prevalence of actual uptake and willingness to use pre-exposure prophylaxis to prevent HIV acquisition among men who have sex with men in Hong Kong, China. *PLoS One.* 2018;13(2):e0191671.

203. Wang L, Moqueet N, Lambert G, et al. Population-Level Sexual Mixing According to HIV Status and Preexposure Prophylaxis Use Among Men Who Have Sex With Men in Montreal, Canada: Implications for HIV Prevention. *Am J Epidemiol.* 2020;189(1):44-54.

204. Wang H, Shobowale O, den Daas C, et al. Determinants of PrEP Uptake, Intention and Awareness in the Netherlands: A Socio-Spatial Analysis. *Int J Environ Res Public Health.* 2022;19(14).
